# Pyridinic Nanographenes by Novel Precursor Design

**DOI:** 10.1002/chem.202004983

**Published:** 2021-01-12

**Authors:** David Reger, Kilian Schöll, Frank Hampel, Harald Maid, Norbert Jux

**Affiliations:** ^1^ Department of Chemistry and Pharmacy &, Interdisciplinary Center for Molecular Materials (ICMM) Friedrich-Alexander-University Erlangen-Nuremberg Nikolaus-Fiebiger-Strasse 10 91058 Erlangen Germany

**Keywords:** heterocycles, nanographene, polycyclic aromatic hydrocarbons, pyridine, Scholl oxidation

## Abstract

In this work we present the solution‐synthesis of pyridine analogues to hexa‐*peri*‐hexabenzocoronene (HBC)—which might be called superpyridines—via a novel precursor design. The key step in our strategy was the pre‐formation of the C−C bonds between the 3/3’ positions of the pyridine and the adjacent phenyl rings—bonds that are otherwise unreactive and difficult to close under Scholl‐conditions. Apart from the synthesis of the parent compound we show that classical pyridine chemistry, namely oxidation, *N*‐alkylation and metal‐coordination is applicable to the π‐extended analogue. Furthermore, we present basic physical chemical characterizations of the newly synthesized molecules. With this novel synthetic strategy, we hope to unlock the pyridine chemistry of nanographenes.

## Graphical abstract

**An efficient Scholl oxidation** to obtain pyridine HBCs is reported in this work. The concept is based on the pre‐formation of bonds at the otherwise unreactive 3/3’ positions of the pyridine. Post‐functionalization at the nitrogen atom by alkylation, oxidation, and coordination is demonstrated together with photophysical and electrochemical characterization of the obtained products.

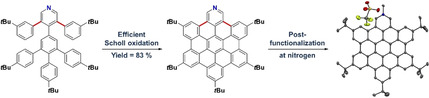

Planar polycyclic aromatic hydrocarbons (PAHs) can be considered as small fragments of graphene. They play a vital role in the world of organic electronics and future materials.^[1]^ Hence the tunability of these nanographenes is of utmost importance to match properties with desired applications. An effective way to achieve this tuning is the incorporation of heteroatoms (mainly: B, N, O, P, S) into the sp^2^ carbon lattice.^[2]^ Arguably the most prominent heteroatom for this purpose is nitrogen as it fits very well into the benzene substructures of PAHs replacing one C−H position. Up to date there are many smaller nitrogen‐containing PAHs known in the literature,^[2a]^ but larger solution‐synthesized ones, which can be considered as real nanographenes (≥1 nm^2^)^[3]^ are fairly rare.^[2]^

One of the most famous and best studied nanographenes, HBC, also called superbenzene and its alkylated, soluble derivatives are known for more than half a century.^[4]^ Pyridine analogues of HBC however, proved to be difficult to synthesise (Figure 1).^[5]^ Most defined, solution‐synthesized nanographenes are made from polyphenylene precursors that are planarized via oxidative cyclodehydrogenation or Scholl‐type reactions.[^1a^, ^2b^, ^6^] This procedure often fails for electron poor aromatics such as pyridines.^[7]^ Pyrimidine containing HBCs can be made nonetheless^[8]^ but for pyridinic ones this chemistry fails, giving only partially closed products. Only one example was published by Draper et al. in which a fully closed terpyridine‐HBC was synthesized. However this compound was only obtained in trace amounts with partially closed species being the major products (Figure 1).^[6]^ The introduction of nitrogen at any desired position into the framework of nanographenes and ultimately carbon allotropes is, however, an appealing target. Such compounds have a variety of potential applications, i.e., the replacement of expensive metal catalysts^[9]^ for oxygen reduction in fuel cells,^[10]^ as electrodes in solar cells,^[11]^ or as active compounds for sensing.^[12]^

**Figure 1:**
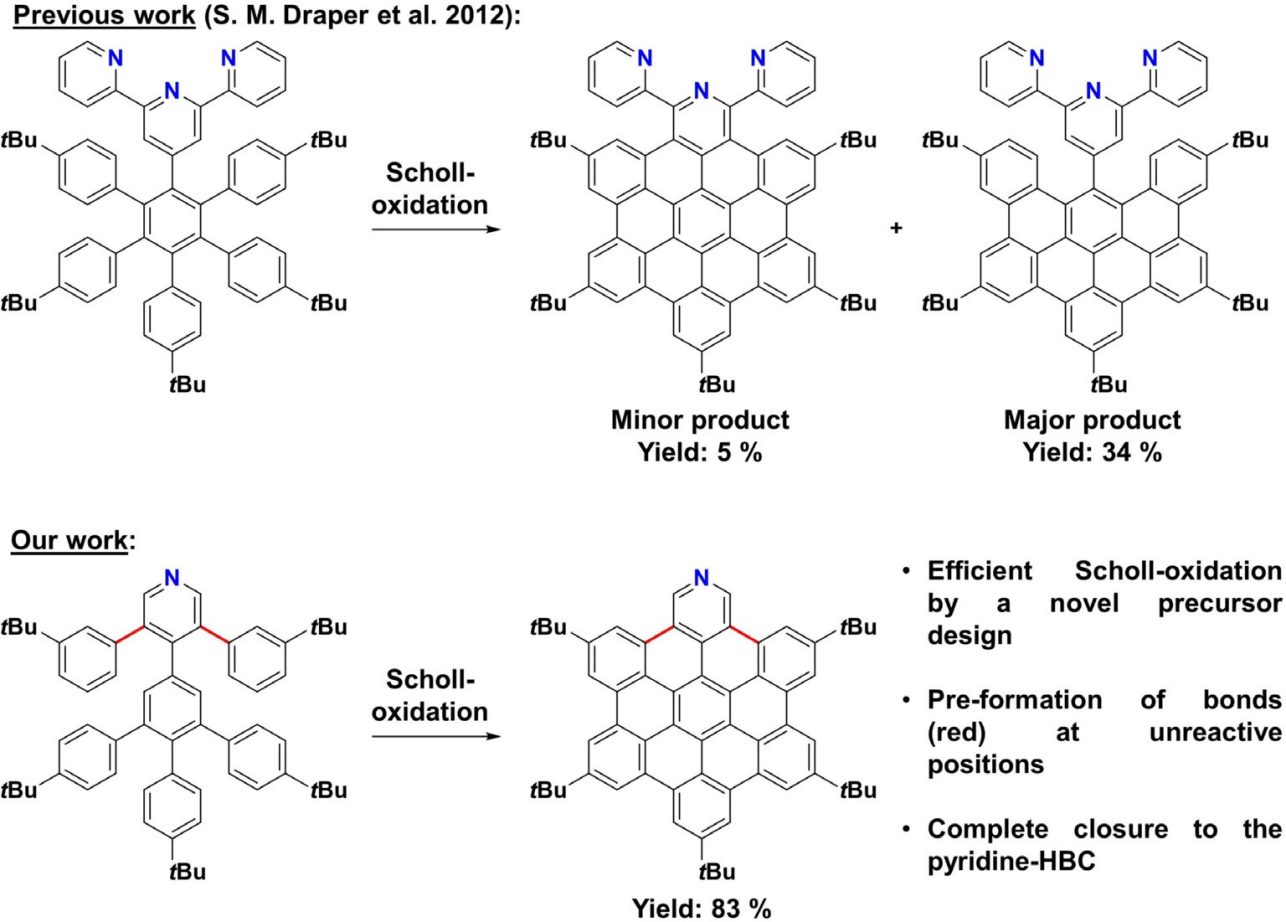
The old and the new. Previous work by Draper et al. for the synthesis of pyridinic HBCs (top)^[5]^ compared to our method via a novel precursor design (bottom).

In this publication, we describe the bottom‐up synthesis of pyridine‐HBCs as nitrogen‐containing nanographenes. This is achieved by a novel precursor design based on the pre‐formation of the bonds at the otherwise unreactive 3/3’ positions of the pyridine. With the introduction of a pyridinic nitrogen, the properties of these nanographenes are altered. Additionally, the nitrogen gives access to protonation, oxidation, substitution and coordination chemistry. *N*‐Substitution and ‐coordination offer a vast variety of possibilities as 2,6‐unsubstituted pyridines allow the interaction and reaction even with sterically demanding partners (e.g. in our case a zinc‐porphyrin). The synthetic protocol (Figure 2) represents a combination of an adaption of our method for the synthesis of highly functionalized hexaarylbenzenes (HABs),^[13]^ the pre‐formation of the C−C bond to non‐activated 3/5‐positions of the pyridine, the formation of a “pseudo‐HAB” precursor and a final Scholl oxidation. In a first attempt, we synthesized pyridine‐HBC **10 a** with three *tert*‐butyl groups in the backbone. However, the solubility of **10 a** was rather low in common organic solvents. Therefore, we decided to attach two additional *tert*‐butyl groups to provide higher solubility. This enables easier synthesis, purification, investigation and further modification of the compound.

**Figure 2:**
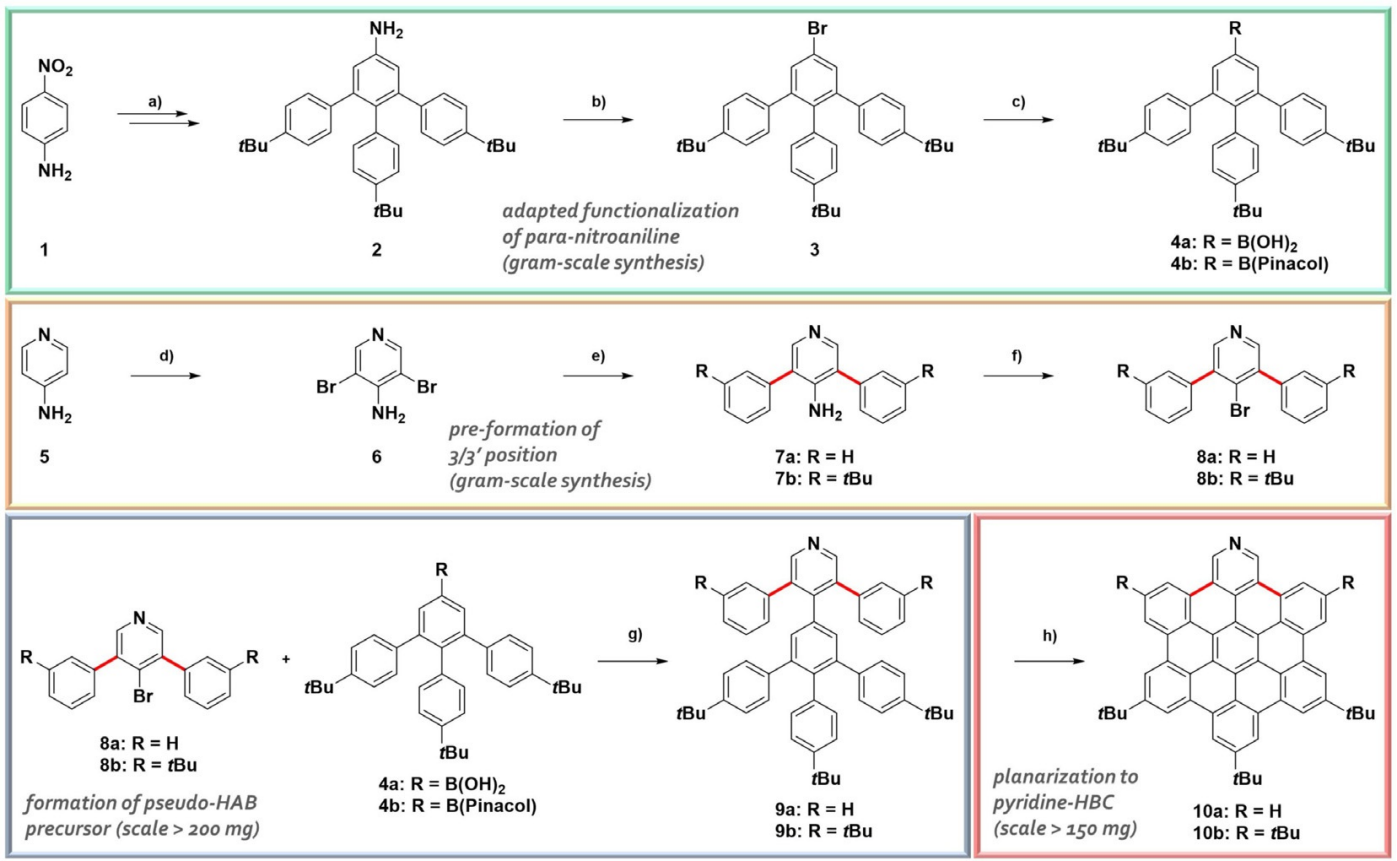
Synthesis of π‐extended pyridines. Green box: Synthesis of “lower‐half” precursors **4 a/b** via functionalization of *para*‐nitroaniline: a) See ref. [13]; b) Isoamyl nitrite (2 equiv.), CHBr_3_, 20 min, 80 °C, N_2_, 47 %; c) **4 a**: 1. *n*BuLi (2.5 m in hexanes, 1.2 equiv.), THF, 1.5 h, −72 °C, N_2_, 2. B(OEt)_3_ (1.5 equiv.) at −72 °C, 24 h, −72 °C to r.t., N_2_, 50 %; **4 b**: KOAc (3 equiv.), bis(pinacolato)diboron (1.1 equiv.), 4.5 mol % Pd(dppf)Cl_2_xCH_2_Cl_2_, 1,4‐dioxane, 20 h, 80 °C, N_2_, 70 %; Orange box: Synthesis of “top‐half” precursors **8 a/b** with pre‐formation of the crucial 3/5 positions (highlighted in red): d) See literature [15]; e) **7 a**: Phenylboronic acid (2.5 equiv.), Na_2_CO_3_ (8 equiv.), 8 mol % Pd(PPh_3_)_3_, toluene:EtOH:H_2_O (10:2:3), 24 h, reflux, N_2_, 98 %; **7 b**: (3‐*tert*‐butylphenyl)boronic acid (2.5 equiv.), Na_2_CO_3_ (8 equiv.), 8 mol % Pd(PPh_3_)_3_, toluene:EtOH:H_2_O (10:2:3), 16 h, reflux, N_2_, 91 %; f) **8 a**: CuBr_2_ (1.2 equiv.), isoamyl nitrite (6 equiv.), CH_3_CN, 24 h, 65 °C, N_2_, 86 %; **8 b**: CuBr_2_ (3 equiv.), isoamyl nitrite (9 equiv.), CH_3_CN, 5 h, 65 °C, 95 %; Blue box: Synthesis of the pseudo‐HAB precursors **9 a/b**: g) **9 a**: **8 a** (1 equiv.)+**4 a** (1.1 equiv.), Cs_2_CO_3_ (2 equiv.), 10 mol % Pd(PPh_3_)_4_, THF:H_2_O (4:1), 17 h, 80 °C, N_2_, 80 %; **9 b**: Method 1: **8 b** (1 equiv.)+**4 a** (1.2 equiv.), Cs_2_CO_3_ (2 equiv.), 10 mol % Pd(PPh_3_)_4_, THF:H_2_O (4:1), 24 h, 80 °C, N_2_, 95 %; Method 2: **8 b** (1 equiv.)+**4 b** (1.2 equiv.), Cs_2_CO_3_ (2 equiv.), 10 mol % Pd(PPh_3_)_4_, THF:H_2_O (4:1), 24 h, 80 °C, N_2_, 79 %; Red box: Synthesis of the final pyridine‐HBCs **10 a/b**: h) **10 a**: DDQ (7 equiv.), triflic acid (14 equiv.), CH_2_Cl_2_, 1 h, 0 °C, N_2_, 81 %, **10 b**: DDQ (7 equiv.), triflic acid (14 equiv.), CH_2_Cl_2_, 4 h, −50 °C to −20 °C, N_2_, 83 %.

The route started with the synthesis of **2** via the procedure for “functionalization of *para*‐nitroaniline”.^[13]^ We continued with a Sandmeyer‐like reaction using isoamyl‐nitrite and bromoform for the exchange of the amine to bromide.^[14]^ The boronic acid derivative **4 a** was obtained via halogen‐lithium exchange followed by addition of triethyl borate and acidic work‐up. The boronic acid pinacol ester derivative **4 b** was prepared via Pd‐coupling chemistry. Overall, the synthesis and purification of **4 b** was easier compared to **4 a**. The pyridinic half of the molecule with the pre‐formed bonds at the 3/5 positions was prepared starting from 4‐aminopyridine which was brominated with *N*‐bromosuccinimide (NBS) via standard procedures.^[15]^ The obtained di‐brominated derivative **6** was then subjected to Suzuki couplings either with phenylboronic acid for **7 a** or with 3‐*tert*‐butylphenylboronic acid for **7 b**. A literature known procedure for the synthesis of **7 a** ^[16]^ was adapted and optimized mainly in terms of work‐up; it was possible to decrease the catalyst loading and to increase the yield from published 47 % to 98 % here. For **7 b** it was possible to achieve an excellent yield of 91 % as well. To finalize the pyridine building block, the amine was converted into the bromide necessary for the following cross‐coupling reaction. Classical Sandmeyer conditions did not give satisfying results and an alternative procedure was necessary. The best results were achieved when the precursors **7 a/b** were heated in acetonitrile in the presence of isoamyl nitrite and CuBr_2_.^[17]^ With the precursors for the top‐ **8 a/b** and the lower‐part **4 a/b** at hand, the formation of the pseudo‐HAB precursor could be carried out via a Suzuki reaction. The products were obtained in excellent yields of up to 80 % for **9 a** and 95 % for **9 b**. The final step to planarize the pyridine‐HBC was achieved under oxidative cyclodehydrogenation conditions with DDQ and triflic acid in CH_2_Cl_2_. Also this last reaction gave highly satisfying yields of 81 % for **10 a** and 83 % for **10 b**. The solubility of the pentakis‐*tert*‐butyl product **10 b** was significantly better which allowed easy handling and purification. In the oxidative cyclodehydrogenation to **10 b** we found that the reaction works best when the triflic acid is added at −50 °C to the reaction mixture containing **9 b** and DDQ in CH_2_Cl_2_. For purification, a simple filtration over a pad of silica gel was usually sufficient. In rare cases small amounts of a not identified side‐product, maybe a partially closed derivative, were formed which could be removed via column chromatography. **10 b** was fully characterized and its formation unambiguously confirmed (for details see Supporting Information). Photophysical characterizations were performed including absorption and emission spectroscopy.

With the solution processable product **10 b** at hand we were able to achieve post‐functionalization at the nitrogen atom (Figure 3). We focused on three different types of reactions: a) coordination; b) alkylation/cationization; c) oxidation. Methylation at the nitrogen atom yielded a pyridinium ion, which was isolated as its triflate salt. The oxidation of **10 b** to its *N*‐oxide alters the properties compared to the pyridine itself and the product could potentially serve as a novel π‐extended pyridine *N*‐oxide ligand. Regarding the coordination chemistry, we demonstrated the interaction with tetrakis‐(4‐*tert*‐butylphenyl)‐zinc‐porphyrin. A ^1^H NMR (Figure 3) of a 1:1 mixture of the zinc‐porphyrin and **10 b** in C_6_D_6_ was measured showing the complex formation with an impressive shift of the 2/6 pyridine protons from 10.54 ppm to 4.60 ppm as they experience the shielding effect of the aromatic ring current in the porphyrin centre. Other protons of **10 b** show this up‐field shift as well, however not that pronounced. In contrast, the signals of the porphyrin are down‐field shifted due to the deshielding effect at the edge of **10 b** (for more details see Supporting Information). This example shows that the extended pyridine derivative **10 b** is able to act as a chromophoric ligand and due to being 2/6 unsubstituted it can even interact with sterically quite shielded metal centres like the Zn‐atom embedded in the porphyrin core. Complex formation could not be observed in UV/Vis or fluorescence spectroscopy as there was no significant change of signals in the mixture compared to the individual components. We assume that the complex formation in high dilutions (10^−6^ mol L^−1^) is not favourable and therefore not observed in photophysical experiments. It was possible to follow this assumption by NMR dilution experiments, showing that the complex is favoured at concentrations greater ≈5×10^−4^ mol L^−1^ (Pyridine 2/6 protons visible at around 4.60 ppm). At lower concentrations (<≈5×10^−4^ mol L^−1^), the signal for the respective protons broadens and finally disappears in the baseline. This indicates a fast exchange instead of an equilibrium favouring the complex (for details see Supporting Information). Still this novel complex is the first example of a supramolecular porphyrin‐HBC conjugate. Covalently connected porphyrin‐nanographene structures became very popular[^18^, ^19^] as model compounds for porphyrin‐graphene hybrids.^[20]^ This novel example could serve as a benchmark compound in further studies to investigate the non‐covalent interactions in such systems.

**Figure 3:**
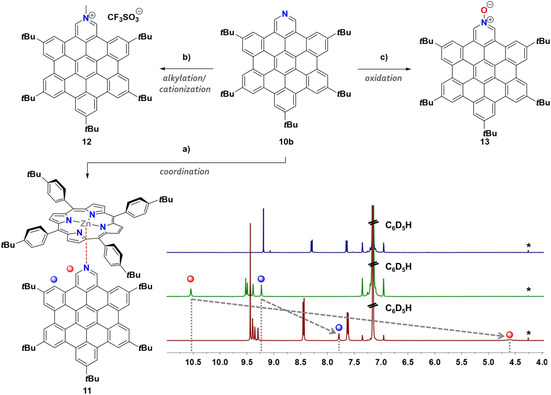
Post‐functionalization of *N*‐HBC **10 b**. a) Coordination to the metal‐center of a zinc‐porphyrin to give the corresponding pyridine complex **11**: tetrakis(4‐*tert*‐butylphenyl)‐zinc‐porphyrin (1 equiv.), C_6_D_6_, r.t. b) formation of the pyridinium salts achieved either by protonation or alkylation (**12** as an example for methylation): 1. MeI (excess), CH_3_CN, 2 h, r.t. N_2_, 2. Ag(OTf) (2.1 equiv.), 15 min, r.t., N_2_, 94 %. c) oxidation to the corresponding pyridine *N*‐oxide **13**: *m*CPBA (1 equiv.), CHCl_3_, 24 h, 0 °C to r.t., 93 %. Bottom right: aromatic signal region of the ^1^H NMR spectra (400 MHz, C_6_D_6_, r.t.) of the zinc‐porphyrin (blue), **10 b** (green) and zinc‐porphyrin‐pyridine complex **11** (dark red). The two most significant shifts and the corresponding hydrogen atoms are marked with red and blue dots. *CH_2_Cl_2_.

Large crystals of **12** suitable for *x*‐ray analysis grew from a saturated CHCl_3_ solution overnight (Figure 4). The dominant motif in the packing of **12** is the formation of π–π aggregated dimers with the two pyridinium moieties on opposite sides. To enable close packing, the two molecules of the dimer are shifted slightly, likely increasing dispersion interactions. Furthermore, the π‐extended cores of the molecules bend inwards resulting in a π–π distance of 3.36 Å which is smaller than the interplanar distance for hexa‐*tert*‐butyl‐HBC with 3.44 Å but very close to the one in graphite with 3.35 Å.^[21]^ This example shows how polarization in the π‐system increases the attractive forces and leads to a closer aggregation. In a larger cutout of the packing (Figure 4 c) the dimers “on top of each other” are well separated with a distance around 9 Å and solvent molecules as well as the respective counterions in between. The dimers “next to each other” are in proximity with their *tert*‐butyl substituents suggesting an aggregation via attractive van der Waals forces. Photophysical data of **10 b**, **12**, **13** and pentakis *tert*‐butyl HBC **14** as a reference substance were measured. Reference **14** is perfectly suited for our purpose as it has the same general structure and symmetry as **10 b**, just replacing the pyridinic nitrogen by C−H. The UV/Vis data (Figure 5 a) shows that the spectra of **10 b** and **14** are very similar. A major difference is observed only when one takes a closer look at the usually symmetry‐forbidden α‐bands^[22]^ at around 420–500 nm. The peaks of oxidized derivative **13** are red shifted compared to **10 b** and overall broadened. For the methylated, cationic pyridinium salt **12** the UV/Vis absorption changes drastically and the typical HBC fine structure of **10 b**, **13** and **14** is lost. Instead an extreme broadening together with a drop in the extinction coefficient in the area between 340–390 nm is observed while on the other hand a significant absorption up to 520 nm is now present. In the fluorescence emission (Figure 5 b) a similar trend is observed. **10 b**, **13** and **14** possess a fine structure with the individual peaks at almost identical wavelengths but with varying relative intensities compared to each other. **12** shows again a different behaviour and exhibits just one very broad emission peak between 480 and 700 nm without a distinct fine structure. As expected, upon protonation with an excess of trifluoroacetic acid (TFA), **10 b** behaves very similar to **12** as observed in UV/Vis and fluorescence experiments (Figure 5 c/d). Now **10 b** shows almost the same broadened and red shifted spectra as **12**. This was also observed when measuring **13** with an excess of TFA. Here we assume that the negatively charged oxygen of the N‐O functionality is protonated leaving the nitrogen positively charged and therefore giving the molecule the pyridinium behaviour as observed before. As expected, reference compound **14** does not respond to an excess of TFA and maintains its absorption and emission features.

**Figure 4:**
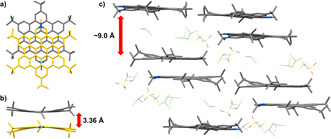
X‐ray structure analysis of **12**. a) top view of a dimer of **12**; b) side view of the dimer showing the π‐planes bending towards each other demonstrating the strong π–π interaction; c) larger cut‐out of the packing showing the dimers separated from each other with the space in between filled with solvent molecules (CHCl_3_) and counterions (CF_3_SO_3_^−^). H atoms were omitted for clarity. For a) and b) solvent molecules and counterions were omitted as well. Deposition number 2021645 contains the supplementary crystallographic data for this paper. These data are provided free of charge by the joint Cambridge Crystallographic Data Centre and Fachinformationszentrum Karlsruhe Access Structures service www.ccdc.cam.ac.uk/structures

**Figure 5:**
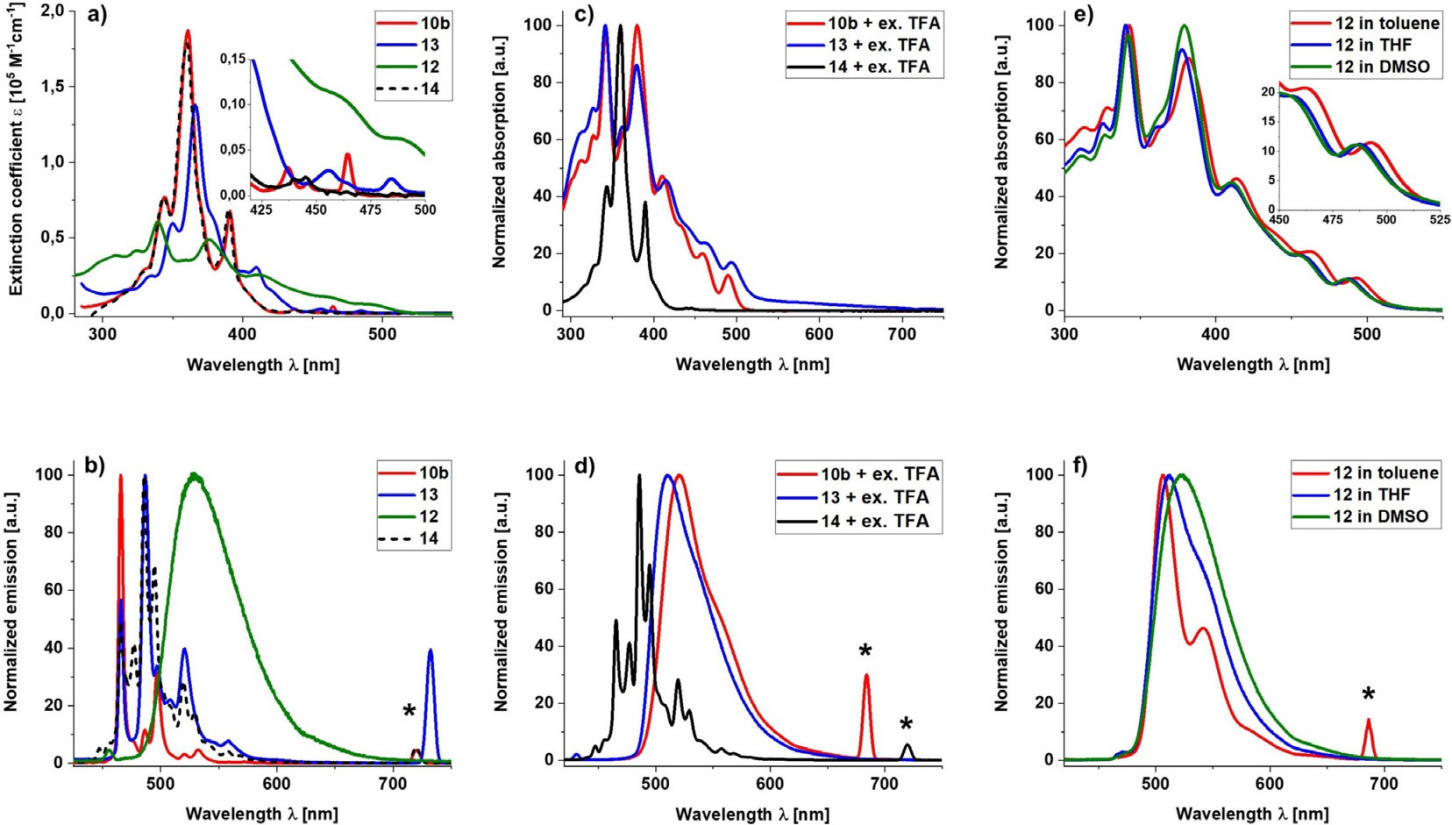
a) Quantitative UV/Vis spectra of **10 b**, **13**, **14** (all in toluene) and **12** (in MeOH). The insert shows an enlargement of the α**‐**bands; b) fluorescence spectra of **10 b** (λ_ex_=360 nm), **13** (λ_ex_=366 nm), **14** (λ_ex_=360 nm) (all in toluene) and **12** (λ_ex_=228 nm) (in MeOH); c) qualitative UV/Vis spectra of **10 b**, **12** and **14** (all in toluene) with an excess of TFA; d) fluorescence spectra of **10 b** (λ_ex_=342 nm), **13** (λ_ex_=380 nm) and **14** (λ_ex_=360 nm) (all in toluene) with an excess of TFA; e) qualitative UV/Vis spectra of **12** in solvents of different polarity. The insert shows an enlargement of the most red**‐**shifted bands; f) fluorescence spectra of **12** in solvents of different polarity: toluene (λ_ex_=343 nm), THF (λ_ex_=340 nm), DMSO (λ_ex_=341 nm). *Artifacts of 2nd order scattering at the double excitation wavelength.

Finally we observed a solvatochromic behaviour of **12** which is demonstrated here with 3 different solvents (Figure 5 e/f) namely toluene, THF and DMSO [solvent polarities: toluene E_T_(30)=33.9 kcal mol^−1^); THF (E_T_(30)=37.4 kcal mol^−1^); DMSO (E_T_(30)=45.1 kcal mol^−1^]^[23]^ (for a broader range of solvents see the Supporting Information). The most bathochromic absorption is detected at 492.5 nm in toluene, 487.5 nm in THF and 485.5 nm in DMSO, respectively. For the emission, the solvatochromic behaviour is more pronounced. Here the lowest energy emission is observed for toluene at 506 nm, for THF at 511.5 nm and for DMSO at 522 nm, respectively. Additionally, in toluene the broad emission peak splits up with a shoulder at 541 nm. For a complete summary of peaks and extinction coefficients see Supporting Information. This solvatochromic behaviour hints to an intramolecular charge transfer character for **12**.

The redox‐features characteristics of **10 b**, **12**, **13** and **14** were determined by CV and DPV measurements. The results are summarized in Table 1. As expected form photophysical characterizations the band gaps for **10 b**, **14** and **13** are very similar (≈3 eV). However, energies for reduction and oxidation are shifted, indicating that **13** is least electron rich and therefore exhibits the highest electron affinity (E_red1_: −1.66 eV) followed by **10 b** (E_red1_: −1.81 eV) and **14** (E_red1_: −1.97 eV). **12** shows by far the smallest band gap (2.54 eV) and highest electron affinity (lowest E_red1_ with −1.07 eV) among all compounds. For details see Supporting Information.

**Table 1:** Electrochemical oxidation and reduction potentials and respective band gaps (V vs. Ag/AgCl) for **10b**, **12**, **13**, **14**. Values were determined in CH_2_Cl_2_ with 0.1 m TBA(PF_6_).

	*E*_red2_ [V]	*E*_red1_ [V]	*E*_ox1_ [V]	*E*_ox2_ [V]	*E*_ox3_ [V]	*E*_gap_ [V]
**14**	–	−1.97	1.06	1.26	–	3.03
**10 b**	–	−1.81	1.14	≈1.4	1.49	2.95
**13**	−1.82	−1.66	1.32	1.53	–	2.98
**12**	–	−1.07	1.47	–	–	2.54

To conclude, we achieved the wet chemical synthesis of the pyridine analogue of HBC. The pre‐formation of the C−C bond at the 3/5 position of the pyridine to the adjacent phenyl rings was the decisive step here. This precursor design allowed an efficient closure of the remaining bonds in the last step by a Scholl oxidation. The synthesis of the final products was easily carried out on a scale >150 mg with room for further improvement. Classical pyridine chemistry such as protonation, *N*‐alkylation, oxidation and coordination to a zinc‐porphyrin worked perfectly fine. In the future the chemistry and physicochemical properties of these novel compounds will be investigated in more detail. We are confident that our pre‐formation strategy of C−C bonds at positions that are unreactive for oxidative cyclodehydrogenation represents a further step towards the on‐demand introduction of nitrogen in PAHs and will ultimately lead to a plethora of novel heteroatom containing nanographenes.

## Conflict of interest

The authors declare no conflict of interest.

## Supporting information

As a service to our authors and readers, this journal provides supporting information supplied by the authors. Such materials are peer reviewed and may be re‐organized for online delivery, but are not copy‐edited or typeset. Technical support issues arising from supporting information (other than missing files) should be addressed to the authors.

Supplementary
