# Supplementary material for: Pyridinic Nanographenes by Novel Precursor Design
Source: Chemistry. 2021 Jan 12;27(6):1984–9. doi: 10.1002/chem.202004983 (PMC7898602; doi:10.1002/chem.202004983)
Supplement: Supplementary file 1 — Supplementary [file CHEM-27-1984-s001.pdf]

Chemistry–A European Journal

Supporting Information

Pyridinic Nanographenes by Novel Precursor Design

David Reger+, Kilian Schöll+, Frank Hampel, Harald Maid, and Norbert Jux*[a]

Table of content

1 GENERAL INFORMATION 2

2 EXPERIMENTAL 3

3 PHOTOPHYSICAL CHARACTERIZATION 15

4 X-RAY DATA FOR 12 22

5 NMR SPECTROSCOPY AND MASS SPECTROMETRY DATA 24

6 ASSIGNMENT OF SIGNALS FOR 10B 52

7 NMR DILUTION EXPERIMENTS 57

8 ELECTROCHEMICAL CHARACTERIZATION 59

9 LITERATURE 62

1 General information

All chemicals were purchased from Sigma-Aldrich and used without any further purification. Solvents were distilled prior to usage. CH_2Cl_2, $CHCl_3$ and EtOAc were distilled from K_2CO_3 prior to usage. Thin layer chromatography (TLC) was performed on Merck silica gel 60 F254, detected by UV-light (254 nm, 366 nm). Column chromatography and flash column chromatography were performed on Macherey–Nagel silica gel 60 M (230–400 mesh, 0.04–0.063 mm). Microwave reactions were carried out in a mono-mode microwave reactor Biotage Initiator+. The microwave assisted reactions were carried out exclusively in the fixed hold time mode. Unless otherwise noted, reactions were degassed via bubbling of N_2-gas through the reaction mixtures. NMR spectroscopy was performed on a Bruker Avance 300 (^{1}H: 300 MHz, ^{13}C: 75 MHz), a Bruker Avance 400 (^{1}H: 400 MHz, ^{13}C: 100 MHz) or a Bruker Avance Neo Cryo-Probe DCH (^{1}H: 600 MHz, ^{13}C: 150 MHz). Deuterated solvents were purchased from Sigma Aldrich and used as received. Chemical shifts are referenced to residual protic impurities in the solvents ($CHCl_3$ ^{1}H: 7.26 ppm, $CDHCl_2$ ^{1}H: 5.32 ppm, C_6HD_5 ^{1}H: 7.16) or the deuterated solvent itself ($CDCl_3$ ^{13}C: 77.0 ppm, $CDHCl_2$ ^{13}C: 53.8 ppm, C_6HD_5 ^{13}C: 128.1 ppm). The resonance multiplicities are indicated as “s“ (singlet), “d” (doublet), “t” (triplet), “q” (quartet) and “m” (multiplet). Signals referred to as bs (broad singlet) are not clearly resolved or significantly broadened. *Para*-substituted phenylrings with an AA’BB’ spin system are termed as duplets even though we are aware that these spin systems give spectra of higher order. The NMR spectra were processed using TopSpin3.5pl7. Exact peak assignment was done with help of 2D NMR techniques (COSY, HSQC, HMBC). LDI/MALDI-ToF mass spectrometry and high resolution mass spectrometry were performed on Bruker ultrafleXtreme (nitrogen UV-laser, 337 nm). In case of MALDI, the following matrices were used: 2,5-dihydroxybenzoic acid (DHB), sinapic acid (SIN) or trans-2-[3-(4-tert-butylphenyl)-2-methyl-2-propenylidene]malononitrile (DCTB). High resolution mass spectrometry was also performed on an ESI-ToF mass spectrometer Bruker maXis 4G UHR MS/MS spectrometer or a Bruker micrOTOF II focus TOF MS-spectrometer. X-ray diffraction analysis was conducted on a Super Nova Dual Wavelength Platform diffractometer by Agilent Technologies GmbH. Steady-state absorption measurements were performed using a Varian Cary 5000 UV-Vis-NIR spectrometer. Samples were measured in a 10 × 10 mm quartz cuvette. Steady state fluorescence measurements were performed using a Shimadzu RF-5301PC Spectrofluorophotometer. Samples were measured in a 10 × 10 mm quartz cuvette. Electrochemical measurements were conducted in a classical three-electrode cell from Deutsche Metrohm GmbH&Co. KG, which was connected to Metrohm Autolab PGSTAT 101, controlled by NOVA 2.1 software. As a working electrode, a motionless gold electrode tip (0.03 cm^2) was used combined with a platinum sheet (1.0 cm^2) that served as a counter electrode. All potentials are presented relative to a Ag/AgCl (2 M LiCl in ethanol) reference electrode with a potential of 0.164 V vs SHE at 21 ± 1 °C. Spectra were recorded in CH_2Cl_2 (HPLC grade) at 21 ± 1 °C with 0.1 M TBA(PF_6) as a supporting electrolyte. For cyclic voltammetry, two different scan rates of 50 and 100 mVs^{-1} were chosen, whereas differential pulse voltammetry was conducted with a scan rate of 10 mVs^{-1}. CH_2Cl_2 was degassed by nitrogen bubbling (2 min/mL) prior to each measurement. The nitrogen atmosphere was maintained during all measurements.

2 Experimental

2.1 Design of "lower-half" via an adapted functionalization of *para*-nitroaniline

Synthesis of the "lower-half" precursors **4a/b** for the pyridine-HBCs. Amine **2** was synthesized according to our procedure developed for the synthesis of highly functionalized hexaarylbenzenes.[S1]

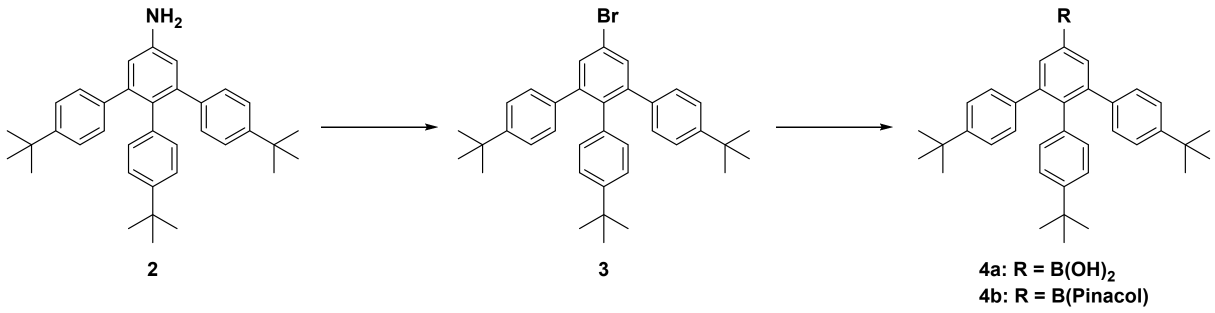

Scheme 1. Synthesis of lower-half precursors **4a/b**.

3,4,5-Tri-(4-*tert*-butylphenyl)-bromobenzene 3

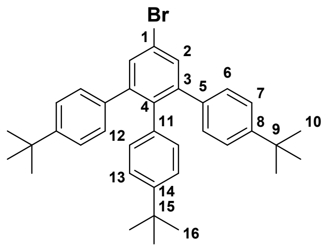

A round-bottom Schlenk-flask (100 mL) equipped with a magnetic stirring bar and a reflux condenser was charged with **2** (5.00 g, 10.2 mmol) and $CHBr_3$. The solution was degassed by N_2-bubbling for 15 min. The solution was heated to 80 °C and isoamyl nitrite (2.34 g, 2.69 mL, 20.4 mmol) was added dropwise. The reaction mixture was stirred for 20 min at 80 °C. After cooling to rt. the dark reaction mixture was washed with sat. aq. $Na_2S_2O_3$ solution (50 mL) and brine (50 mL). The organic phase was dried over $MgSO_4$. After filtration and evaporation of the solvents the remaining black oil was filtered over a pad of silica gel (10 x 5 cm) with hexanes. All fractions containing the product were collected. After evaporation of the solvents a colorless oil was obtained. After addition of methanol (30 mL) the product precipitated and was filtered off through a glass-frit (P4). The product was dried under vacuum and obtained as a white solid in a yield of 47 % (2.64 g, 4.77 mmol).

^{1}H NMR ($CDCl_3$, 400 MHz, rt.): δ [ppm] = 7.57 (s, 2H, 2), 7.15 (d, ^3J = 8.5 Hz, 4H, 7), 6.99 – 6.94 (m, 6H, 6/13), 6.67 (d, ^3J = 8.5 Hz, 2H, 12), 1,26 (s, 18H, 10), 1.18 (s, 9H, 16).

^{13}C NMR ($CDCl_3$, 100 MHz, rt.): δ [ppm] = 149.5 (14), 149.0 (8), 144.0 (3), 138.6 (4), 137.9 (5), 135.8 (11), 131.9 (2), 131.2 (12), 129.5 (6), 124.5 (7), 124.0 (13), 120.8 (1), 34.5 (9), 34.4 (15), 31.41 (10), 31.37 (16).

**HRMS (APPI)**:	m/z (calc. for $C_{36}H_{41}Br$ [M^+]):	552.2386
	m/z (measured):	552.2399
	error [ppm]:	-2.2

3,4,5-Tri-(4-*tert*-butylphenyl)phenylboronic acid 4a

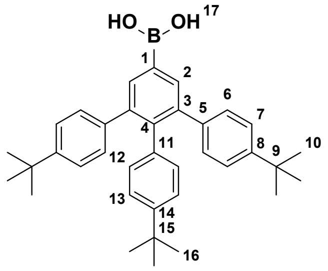

A flame dried Schlenk-tube (Ø 2.5 cm) was charged with **3** (0.50 g, 0.90 mmol) and anhydrous THF (7 mL). The solution was degassed by N_2 bubbling for 5 min. After cooling to -72 °C, 2.5 M *n*-BuLi in hexanes (0.43 mL, 1.08 mmol) was added dropwise and the reaction mixture was stirred for 1.5 h and $B(OEt)_3$ (0.23 mL, 0.20 g, 1.36 mmol) was added dropwise. The reaction mixture was allowed to warm to rt. overnight. It was poured onto 1 M aq. HCl (45 mL) and stirred for 2 h at rt. EtOAc (30 mL) was added and the phases were separated. The aqueous phase was extracted with CH_2Cl_2 (3 x 30 mL) and the combined organic phases were dried over $MgSO_4$. The product was precipitated from CH_2Cl_2 and hexanes and was dried under vacuum. It was obtained in a yield of 50 % (0.24 g, 0.46 mmol).

Note: NMR spectra show two sets of signals. One belongs to the free boronic acid and shows OH proton signals. The second, more intense one (marked with ') belongs to some dimeric/trimeric species that does not show OH proton signals. The ^{13}C signals of position 1/1' was not observed because of line broadening due to the short relaxation time and the quadrupole moment of boron-11 (I = 3/2).[S2]

^1H NMR ($CDCl_3$, 300 MHz, rt.): δ [ppm] = 8.26 (s, 2H, 2'), 7.77 (s, 0.4H, 2), 7.15 (d, 3J = 8.5 Hz, 4+0.8H, 7'+7), 7.05 (d, 3J = 8.5 Hz, 4H, 6'), 7.00 (d, 3J = 8.5 Hz, 0.8H, 6), 6.96 (d, 3J = 8.5 Hz, 2+0.4H, 13'+13), 6.72 (d, 3J = 8.5 Hz, 2H, 12'), 6.72 (d, 3J = 8.5 Hz, 0.4H, 12), 1.26 (s, 3.6H, 10), 1.26 (s, 18H, 10'), 1.19 (s, 9+1.8H, 16'+16).

^{13}C NMR ($CDCl_3$, 100 MHz, rt.): δ [ppm] = 149.1 (14'/8'/14/8), 148.9 (14'/8'/14/8), 148.8 (14'/8'/14/8), 144.0 (11'), 142.5 (11), 141.8 (5), 141.7 (5'), 139.1 (3'+3), 136.8 (4'+4), 136.5 (2'), 134.5 (2), 131.1 (12'+12), 129.8 (6'), 129.6 (6), 124.4 (7'/7), 124.4 (7'/7), 124.0 (13'/13), 123.9 (13'/13), 34.4 (9'+9), 34.4 (15'+15), 31.5 (10'+10), 31.4 (16'+16).

MS (MALDI): No product peak detected.

3,4,5-Tri-(4-*tert*-butylphenyl)phenylboronic acid pinacol ester 4b

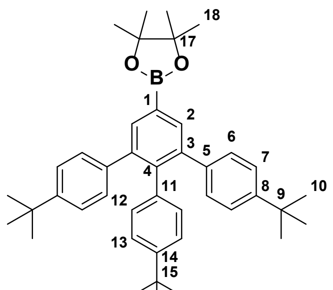

A round-bottom Schlenk-flask (100 mL) was charged with **3** (1.53 g, 2.76 mmol), KOAc (0.81 g, 8.28 mmol), bis(pinacolato)diboron (0.772 g, 3.04 mmol), and 1,4-dioxane (25 mL). The solution was degassed by N_2 bubbling for 10 min, $Pd(dppf)Cl_2 \cdot CH_2Cl_2$ (0.101 g, 0.124 mmol) was added and the reaction mixture was stirred at 80 °C for 20 h. After cooling to rt., the solvent was evaporated, H_2O (30 mL) was added and the mixture was extracted with CH_2Cl_2 (3 x 30 mL). The combined organic phases were washed with brine (1 x 30 mL) and the solvent was evaporated. Purification was achieved by column chromatography over silica gel (4.5 x 9 cm) starting with CH_2Cl_2/hexanes 1:4 as eluent and then increasing the eluent polarity to CH_2Cl_2/hexanes 2:3. The column was operated as fast as possible to minimize decomposition of the pinacol ester on the column. The product was dried under vacuum and obtained as white solid in a yield of 70 % (1.17 g, 1.94 mmol).

^1H NMR ($CDCl_3$, 400 MHz, rt.): δ [ppm] = 7.89 (s, 2H, 2), 7.13 (d, ^3J = 8.3 Hz, 4H, 7), 7.01 (d, ^3J = 8.3 Hz, 4H, 6), 6.96 (d, ^3J = 8.3 Hz, 2H, 13), 6.73 (d, ^3J = 8.3 Hz, 2H, 12), 1.35 (s, 12H, 18), 1.25 (s, 18H, 10), 1.18 (s, 9H, 16).

^{13}C NMR (CDCl$_3$, 100 MHz, rt.): *δ* [ppm] = 148.8 (8), 148.7 (14), 142.3 (11), 141.5 (5), 139.1 (3), 136.8 (4), 135.9 (2), 131.2 (12), 129.7 (6), 124.3 (7), 123.9 (13), 83.9 (17), 34.4 (9), 34.4 (15), 31.5 (10), 31.4 (16), 25.0 (18).

MS (LDI): m/z (rel. int.) = 623 [M+Na]$^+$ (100 %).

**HRMS (MALDI, DCTB)**:	m/z (calc. for $C_{42}H_{53}BO_2$ [M$^+$]):	600.4133
	m/z (measured):	600.4140
	error [ppm]:	1.4

2.2 Design of "top-half" via pre-formation of the 3/3' C-C bond of the pyridine

Synthesis of the top half precursors **8a/b** by direct attachment of the adjacent phenyl rings. Precursor **6** was synthesized according to literature procedures.[S3]

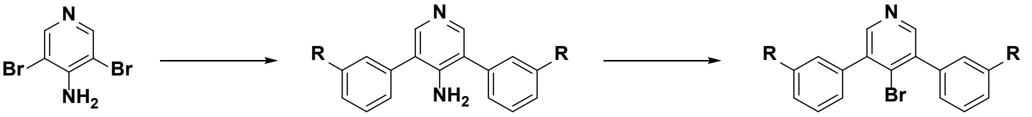

6 **7a: R = H** **7b: R = *t*Bu** **8a: R = H** **8b: R = *t*Bu**

Scheme 2. Synthesis of top-half precursors **8a/b**.

3,5-diphenyl-4-amiopyridine 7a

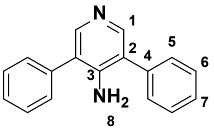

A round-bottom Schlenk-flask (500 mL) equipped with a magnetic stirring bar and a reflux condenser was charged with **6** (2.00 g, 7.94 mmol), phenylboronic acid (2.42 g, 19.85 mmol), Na_2CO_3 (6.73 g, 63.5 mmol), toluene (100 mL), ethanol (20 mL) and H_2O (30 mL). The mixture was degassed by N_2 bubbling for 30 min. $Pd(PPh_3)_4$ (0.73 g, 0.63 mmol) was added and the reaction mixture was stirred under reflux for 24 h. After cooling to rt., the phases were separated, the organic phase was washed with brine (50 mL) and then extracted with 2 M aq. HCl (3 x 50 mL). The extracts were washed with Et_2O (100 mL) and an alkaline pH-value was adjusted by adding solid Na_2CO_3 under vigorous stirring. A white precipitate formed which was filtered off through a glass-frit (P4) and washed with water. The product was dried under vacuum and was obtained as a white solid in a yield of 98 % (1.92 g, 7.80 mmol).

^{1}H NMR (CDCl$_3$, 400 MHz, rt.): *δ* [ppm] = 8.16 (s, 2H, 1), 7.52–7.47 (m, 8H, 5/6), 7.43–7.38 (m, 2H, 7), 4.37 (s, 2H, 8).

^{13}C NMR (CDCl$_3$, 100 MHz, rt.): *δ* [ppm] = 149.3 (1), 147.4 (3), 136.0 (4), 129.4 (5/6), 129.3 (5/6), 128.1 (7), 122.7 (2).

3,5-diphenyl-4-bromopyridine 8a

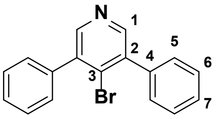

A Schlenk-tube (Ø 3 cm) equipped with a magnetic stirring bar and a reflux condenser was charged with $CuBr_2$ (0.54 g, 2.44 mmol) and acetonitrile (15 mL). The mixture was degassed via N_2 bubbling for 10 min. Isoamyl nitrite (0.36 g, 0.41 mL, 3.05 mmol) was added and the

solution was stirred for 15 min at rt. **7a** (0.50 g, 2.03 mmol) was added and the resulting suspension was stirred for 24 h at 65 °C. To achieve complete conversion after 2.5 h, 3.5 h and 5 h additional portions of isoamyl nitrite (each: 0.36 g, 0.41 mL, 3.05 mmol) were added. After cooling to rt. The solvent was evaporated and the remaining solids were suspended in CH_2Cl_2. The suspension was washed with 25 % aq. ammonia solution (25 mL) which resulted in dissolving of the solids. The aqueous phase was extracted with CH_2Cl_2 (3 x 30 mL) and the combined organic phases were washed with brine (50 mL) and dried over $MgSO_4$. All solvents were evaporated and the crude, yellow product was purified by filtration over a pad of silica gel (5 x 5 cm) with CH_2Cl_2. The product was dried under vacuum and obtained as a off white solid in a yield of 86 % (0.54 g, 1.74 mmol).**^{1}H NMR ($CDCl_3$, 400 MHz, rt.):** *δ* [ppm] = 8.46 (s, 2H, 1), 7.51 – 7.43 (m, 10H, 5+6+7).

^{13}C NMR ($CDCl_3$, 100 MHz, rt.): *δ* [ppm] = 149.6 (1), 139.3 (2), 138.04 (4), 133.6 (3), 129.8 (6), 128.5 (7), 128.4 (5).

HRMS (APPI, CH_2Cl_2, acetonitrile): m/z (calc. for $C_{17}H_{13}BrN$ $[M+H]^+$): 310.0226

m/z (measured): 310.0226

error [ppm]: 0.0

3,5-di-(3-*tert*-butyl-phenyl)-4-aminopyridine 7b

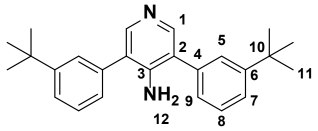

A round-bottom Schlenk-flask (500 mL) equipped with a magnetic stirring bar and a reflux condenser was charged with **6** (2.00 g, 7.94 mmol), (3-*tert*-butylphenyl)boronic acid (3.53 g, 19.9 mmol), Na_2CO_3 (6.73 g, 63.5 mmol), toluene (100 mL), EtOH (20 mL) and H_2O (30 mL). The mixture was degassed via N_2 bubbling for 30 min. $Pd(PPh_3)_4$ (0.73 g, 0.64 mmol) was added and the and the reaction was stirred for 16 h under reflux. After cooling to rt. The phases were separated and the organic phase was washed with H_2O (30 mL) and brine (30 mL). 2 M aq. HCl (100 mL) were added to the organic phase and a white solid precipitated. The aqueous phase was removed and the remaining suspension was filtered through a glass-frit (P4). The residue was washed with toluene and H_2O. The white solid was dissolved in a mixture of MTBE (50 mL) and sat. aq. $NaHCO_3$ (50 mL). The phases were separated, the aqueous phase was extracted with MTBE (20 mL) and the combined organic phases were dried over $MgSO_4$. After evaporation of the solvent the solids were dried under vacuum. The product was obtained as a colorless solid in a yield of 91 % (2.43 g, 6.78 mmol).

^{1}H NMR ($CDCl_3$, 400 MHz, rt.): *δ* [ppm] = 8.18 (s, 2H, 1), 7.51 – 7.50 (m, 2H, 5), 7.45 – 7.40 (m, 4H, 8+7), 7.32 – 7.29 (m, 2H, 9), 4.40 (s, 2H, NH_2), 1.37 (s, 18H, 11).

^{13}C NMR ($CDCl_3$, 100 MHz, rt.): *δ* [ppm] = 152.4 (6), 149.2 (1), 147.6 (3), 135.7 (4), 129.0 (8), 126.5 (5), 126.4 (9), 125.1 (7), 123.2 (2), 35.0 (10), 31.5 (11).

HRMS (ESI, CH_2Cl_2, acetonitrile): m/z (calc. for $C_{25}H_{31}N_2$ $[M+H]^+$): 359.248175

m/z (measured): 359.248458

error [ppm]: 0.8

3,5-di-(3-*tert*-butyl-phenyl)-4-bromopyridine 8b

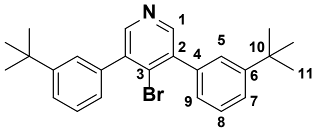

A round-bottom Schlenk-flask (500 mL) equipped with a magnetic stirring bar and a reflux condenser was charged with **7b** (2.44 g, 6.81 mmol) and acetonitrile (100 mL). $CuBr_2$ (4.56 g, 20.4 mmol) was added and the solution was degassed via N_2 bubbling for 20 min. Isoamyl nitrite (4.79 g, 5.50 mL, 40.9 mmol) was added and the reaction mixture was stirred for 3 h at 65 °C. For complete conversion another portion of isoamyl nitrite (2.39 g, 2.75 mL, 20.4 mmol) was added and the reaction mixture was stirred for further 2 h at 65 °C. Urea (0.5 g) was added after total of 5 h. All solvents were evaporated, the remaining solids were re-dissolved in CH_2Cl_2 (50 mL) and 25 % aq. ammonia solution (30 mL) was added. The phases were separated and the organic phase was washed with 25 % aq. ammonia solution (30 mL) and H_2O (2 x 30 mL) and dried over $MgSO_4$. After evaporation of all solvents and yellow oil was obtained which was filtered over a pad of silica gel (5 x 5 cm) with CH_2Cl_2. The product was dried under vacuum and was obtained as a colorless oil that solidifies slowly in a yield of 95 % (2.72 g, 6.45 mmol).

^{1}H NMR ($CDCl_3$ + TFA, 400 MHz, rt.): δ [ppm] = 8.61 (s, 2H, 1), 7.63 – 7.61 (m, 2H, 7), 7.53 – 7.49 (m, 4H, 8+5), 7.30 – 7.28 (m, 2H, 9), 1.38 (s, 18H, 11).

^{13}C NMR ($CDCl_3$ + TFA, 100 MHz, rt.): δ [ppm] = 152.7 (6), 147.3 (3), 145.2 (2), 139.1 (1), 133.8 (4), 129.1 (8), 127.7 (7), 126.5 (5), 126.3 (9), 35.1 (10), 31.2 (11).

**HRMS (APPI, toluene)**:	m/z (calc. for $C_{25}H_{29}BrN$ $[M+H]^+$):	422.1478
	m/z (measured):	422.1482
	error [ppm]:	1.0

2.3 Formation of pseudo-HABs

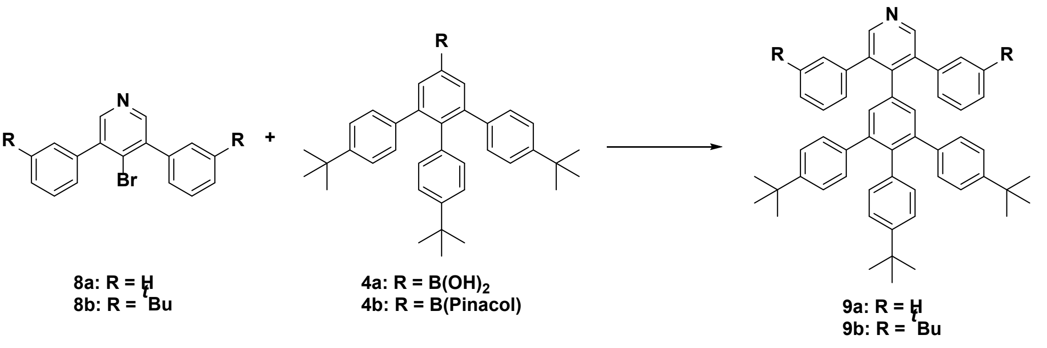

Scheme 3. Synthesis of pseudo-HABs **9a/b**.

Tris-*tert*-butyl pseudo-pyridine HAB 9a (from 4a and 8a)

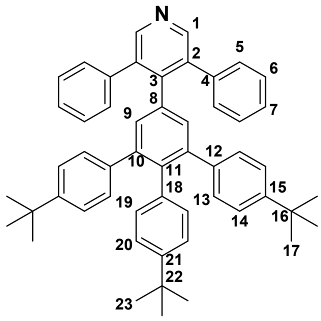

A pressure-vial (5 mL) equipped with a magnetic stirring bar was charged with **8a** (0.10 g, 0.32 mmol) Cs_2CO_3 (0.21 g, 0.64 mmol), THF (2 mL) and H_2O (0.5 mL). The mixture was degassed via N_2 bubbling for 5 min and finally **4a** (0.20 g, 0.39 mmol) and $Pd(PPh_3)_4$ (37.0 mg, 32.0 µmol) were added. The vial was closed and the reaction mixture was stirred for 17 h at 80 °C. After cooling to rt. CH_2Cl_2 and H_2O were added until all solids were dissolved. The phases were separated, the aqueous phase was extracted with CH_2Cl_2 (3 x 10 mL) and the combined organic phases were dried over $MgSO_4$. After evaporation of the solvents the crude product was purified via column chromatography over silica gel (4 x 14 cm) with CH_2Cl_2. The product was dried under vacuum and was obtained as a white solid in a yield of 80 % (0.18 g, 0.26 mmol).

^{1}H NMR (CD_2Cl_2, 400 MHz, rt.): *δ* [ppm] = 8.68 (br, 2H, 1), 7.43–7.41 (m, 6H, 5+7), 7.28–7.26 (m, 4H, 6), 7.01 (d, ^3J = 8.4 Hz, 4H, 14), 6.96 (d, ^3J = 8.4 Hz, 2H, 20), 6.84 (s, 2H, 9), 6.58 (d, ^3J = 8.4 Hz, 2H, 19), 6.42 (d, ^3J = 8.4 Hz, 4H, 13), 1.21 (s, 18H, 17), 1.18 (s, 9H, 23).

^{13}C NMR ($CDCl_3$, 100 MHz, rt.): *δ* [ppm] = 149.9 (1), 148.9 (15), 148.8 (21), 145.9 (3), 141.0 (10), 138.6 (2/4/12), 138.6 (2/4/12), 138.1 (11), 136.3 (18), 134.7 (8), 132.2 (9), 131.2 (19), 130.5 (6), 129.5 (13), 128.4 (5), 127.3 (7), 124.1 (14), 123.9 (20), 34.4 (16), 34.3 (22), 31.4 (17+23).

MS (MALDI, DHB): m/z (rel. int.) = 704 $[M+H]^+$ (100 %).

**HRMS (MALDI, DCTB)**:	m/z (calc. for $C_{53}H_{53}N$ $[M]^+$):	703.4173
	m/z (measured):	703.4174
	error [ppm]:	0.3

Pentakis-*tert*-butyl pseudo-pyridine HAB 9b (from 4a and 8b)

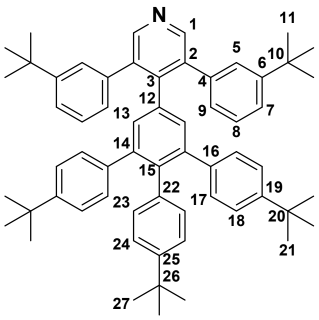

A pressure vial (5 mL) was charged with **8b** (0.14 g, 0.32 mmol), Cs_2CO_3 (0.21 g, 0.64 mmol), THF (2 mL) and H_2O (0.5 mL). The mixture was degassed via N_2 bubbling for 5 min. **4a** (0.20 g, 0.39 mmol) and $Pd(PPh_3)_4$ (37 mg, 32 µmol) were added and the vial was closed. The reaction mixture was stirred for 24 h at 80 °C. After cooling to rt. EtOAc (10 mL) and H_2O (10 mL) were added, the phases were separated and the aqueous phase was extracted with CH_2Cl_2 (3 x 10 mL). The combined organic phases were dried over $MgSO_4$ and the solvent was evaporated. Purification was achieved by column chromatography on silica gel (3 x 15 cm) with CH_2Cl_2. With the product eluting the solvents were switched to CH_2Cl_2/EtOAc 5/1 to narrow the product distribution on the column. The product was dried under vacuum and obtained as a white solid in a yield of 95 % (0.25 g, 0.31 mmol).

For the analytical data please check the procedure below. ^{1}H NMR, ^{13}C NMR and MS data are equivalent to the data obtained for the synthesis of **9b** from **4b** and **8b**.

Pentakis-*tert*-butyl pseudo-pyridine HAB 9b (from 4b and 8b)

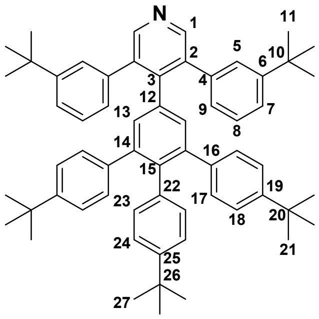

A pressure vial (5 mL) equipped with a magnetic stirring bar was charged with **8b** (0.16 g, 0.39 mmol), Cs_2CO_3 (0.25 g, 0.77 mmol), THF (2 mL) and H_2O (0.5 mL). The mixture was degassed via N_2 bubbling for 5 min and finally **4b** (0.28 g, 0.46 mmol) and $Pd(PPh_3)_4$ (45.0 mg, 39.0 µmol) were added. The vial was closed and the reaction mixture was stirred for 24 h at 80 °C. After cooling to rt. CH_2Cl_2 (10 mL) and H_2O (10 mL) were added, the phases were separated and the aqueous phase was extracted with CH_2Cl_2 (3 x 10 mL). The combined organic phases were dried over $MgSO_4$. After evaporation of the solvents the crude product was purified by flash chromatography eluting with a gradient of hexanes to CH_2Cl_2. The product was dried under vacuum and was obtained as a white solid in a yield of 79 % (0.25 g, 0.31 mmol).

^{1}H NMR ($CDCl_3$, 400 MHz, rt.): δ [ppm] = 8.74 (s, 2H, 1), 7.49 – 7.46 (m, 2H, 8), 7.45 – 7.42 (m, 2H, 7), 7.33 – 7.31 (m, 2H, 9), 7.03 – 7.02 (m, 2H, 5), 6.97 (d, ^3J = 8.4 Hz, 4H, 18), 6.89 (d, ^3J = 8.4 Hz, 2H, 24), 6.82 (s, 2H, 13), 6.47 (d, ^3J = 8.4 Hz, 2H, 23), 6.35 (d, ^3J = 8.4 Hz, 4H, 17), 1.21 (s, 18H, 21), 1.16 (s, 9H, 27), 1.16 (s, 18H, 11).

^{13}C NMR ($CDCl_3$, 100 MHz, rt.): δ [ppm] = 150.9 (6), 149.6 (1), 148.8 (19), 148.7 (25), 146.1 (3), 141.4 (14), 138.8 (16), 137.8 (4), 137.8 (15), 137.1 (2), 136.3 (22), 135.6 (12), 132.2 (13), 131.1 (23), 129.4 (17), 129.1 (5), 128.4 (8), 126.9 (9), 124.2 (18), 124.0 (7), 123.9 (24), 34.7 (10), 34.4 (20), 34.3 (26), 31.4 (11/21/27), 31.4 (11/21/27).

MS (MALDI, DCTB): m/z (rel. int.) = 817 $[M+H]^+$ (100 %).

**HRMS (MALDI, DCTB)**:	m/z (calc. for $C_{61}H_{70}N$ $[M+H]^+$):	816.5503
	m/z (measured):	816.5494
	error [ppm]:	1.1

2.4 Planarization to pyridine-HBCs

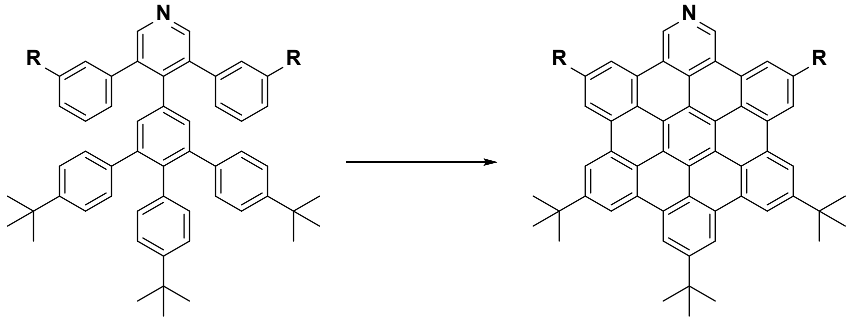

9a: R = H
9b: R = *t*Bu

10a: R = H
10b: R = *t*Bu

Scheme 4. Synthesis of pyridine-HBCs **10a/b**.

π-extended pyridine HBC 10a

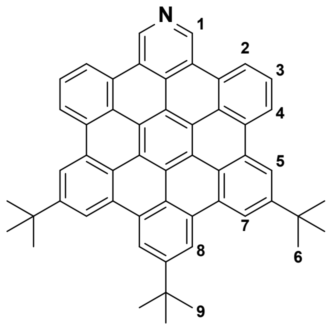

A Schlenk-tube (Ø 3 cm) equipped with a magnetic stirring bar was charged with **9a** (50.0 mg, 71.0 µmol) and CH_2Cl_2 (15 mL). The solution was degassed via N_2 bubbling for 5 min and cooled to 0 °C. DDQ (0.11 g, 0.50 mmol) and finally triflic acid (0.15 g, 0.09 mL, 0.99 mmol) were added and the tube was closed via a rubber septum. The reaction mixture was stirred for 1 h at 0 °C and was subsequently quenched with MeOH (20 mL) and NEt_3 (5 mL). All solvents were evaporated and the brown residue was suspended in THF (10 mL) and NEt_3 (1 mL) under ultrasonication. The suspension was stirred for 2 h at rt. MeOH (50 mL) was added and the solids were sedimented in a centrifuge (30 min at 5000 rpm). The supernatant was decanted and the solids were again suspended in MeOH (40 mL). This washing process (suspending in MeOH, centrifugation and decantation) was repeated five times. The remaining solids were dried under vacuum. The product was obtained as an orange powder in a yield of 81 % (40.0 mg, 58.0 µmol).

The product was insoluble in most common organic solvents. Tiny amounts could be dissolved in CS_2, hot toluene or ODCB. Adding a droplet of NEt_3 increases the solubility.

^{1}H NMR (CS_2/$CDCl_3$/NEt_3, 600 MHz, rt.): δ [ppm] = 10.12 (s, 2H, 1), 9.21 (s, 2H, 8), 9.19 (s, 2H, 7/5), 9.13 (s, 2H, 7/5), 9.09 (d, ^3J = 7.7 Hz, 2H, 2/4), 9.07 (d, ^3J = 7.7 Hz, 2H, 2/4), 8.14 (t, ^3J = 7.7 Hz, 2H, 3), 1.89 (s, 9H, 9), 1.86 (s, 18H, 6).

The solubility was too low to obtain proper ^{13}C NMR data.

MS (MALDI, LDI): m/z (rel. int.) = 692 [M+H]$^+$ (100 %).

UV/Vis (toluene, rt.) λ_{max} [nm] (rel. Int.): 343 (46%), 359 (100%), 389 (39%), 436 (6%), 444 (4%), 463 (5%).

Emission (toluene, rt., exc. 360 nm) λ_{max} [nm] (rel. Int.): 464 (100%), 485 (17%), 495 (32%), 518 (5%), 530 (5%).

Soluble π-extended pyridine HBC 10b

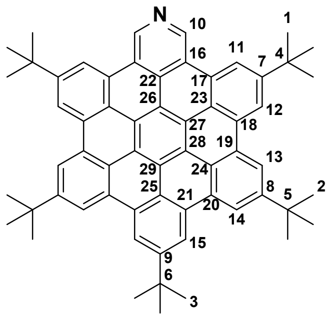

A round-bottom Schlenk-flask (250 mL) equipped with a magnetic stirring bar was charged with **9b** (0.24 g, 0.29 mmol) and CH_2Cl_2 (71 mL). The solution was cooled to -50 °C (solid CO_2, acetonitrile) and degassed via N_2 bubbling for 15 min. DDQ (0.47 g, 2.02 mmol) and finally triflic acid (0.61 g, 0.36 mL, 4.10 mmol) were added and the flask was closed via a rubber septum. The reaction mixture was stirred for 4 h and allowed to warm from -50 °C to -20 °C over that period. It was quenched by addition of MeOH (20 mL) and NEt_3 (1 mL) and all solvents were evaporated. The crude was adsorbed onto silica gel and pre-purified via a filtration over a pad of silica gel (5 x 3 cm) with THF/hexanes 4/1 + 2vol% NEt_3. Final purification was achieved by column chromatography over silica gel (4 x 20 cm) with THF/hexanes 4/1 + 2vol% NEt_3 and subsequent precipitation from a concentrated THF solution by addition of MeOH. The yellow precipitated was filtered off through a glass-frit (P4) and washed with MeOH. The product was dried under vacuum and was obtained as a yellow solid in a yield of 83 % (0.19 g, 0.24 mmol).

^{1}H NMR ($CDCl_3$ + NEt_3, 600 MHz, rt.): *δ* [ppm] = 9.96 (s, 2H, 10), 9.17 (s, 2H, 15), 9.12 (s, 2H, 14), 9.04 (s, 2H, 13), 8.99 (s, 2H, 12), 8.94 (s, 2H, 11), 1.89 (s, 9H, 3), 1.84 (s, 18H, 2), 1.77 (s, 18H, 1).

^{13}C NMR ($CDCl_3$ + NEt_3, 150 MHz, rt.): *δ* [ppm] = 149.2 (9), 149.1 (7), 148.8 (8), 142.2 (10), 130.6 (21), 130.2 (20), 130.1 (18), 130.0 (19), 129.0 (22), 128.1 (17), 123.8 (16), 123.4 (23+25), 123.3 (24), 121.5 (29), 120.9 (27), 119.9 (28), 119.2 (12), 119.0 (14), 119.0 (15), 118.8 (13), 118.3 (11), 117.4 (26), 35.9 (6), 35.8 (5), 35.8 (4), 32.3 (3), 32.2 (2), 32.1 (1).

MS (MALDI, DCTB): m/z (rel. int.) = 803 $[M]^+$ (100 %).

**HRMS (LDI)**:	m/z (calc. for $C_{61}H_{57}N$ $[M]^+$):	803.4486
	m/z (measured):	803.4475
	error [ppm]:	1.3

UV/Vis (THF, rt.) λ_{max} [nm] (ε [M^{-1} cm^{-1}]): 342 (86000), 359 (206000), 389 (63000), 436 (3500), 446 (1500), 464 (5000).

UV/Vis (toluene, rt.) λ_{max} [nm] (ε [M^{-1} cm^{-1}]): 344 (77000), 361 (187000), 391 (68000), 437 (3100), 446 (1300), 464 (4500).

Emission (THF, rt., exc. 360 nm) λ_{max} [nm] (rel. Int.): 465 (100%), 486 (11%), 496 (32%), 520 (3%), 531 (5%).

Emission (toluene, rt., exc. 360 nm) λ_{max} [nm] (rel. Int.): 466 (100%), 486 (12%), 497 (31%), 520 (3%), 532 (5%).

2.5 Post-functionalization

Post-functionalization of **10b** to obtain **12** by methylation, **13** by oxidation and **11** by coordination.

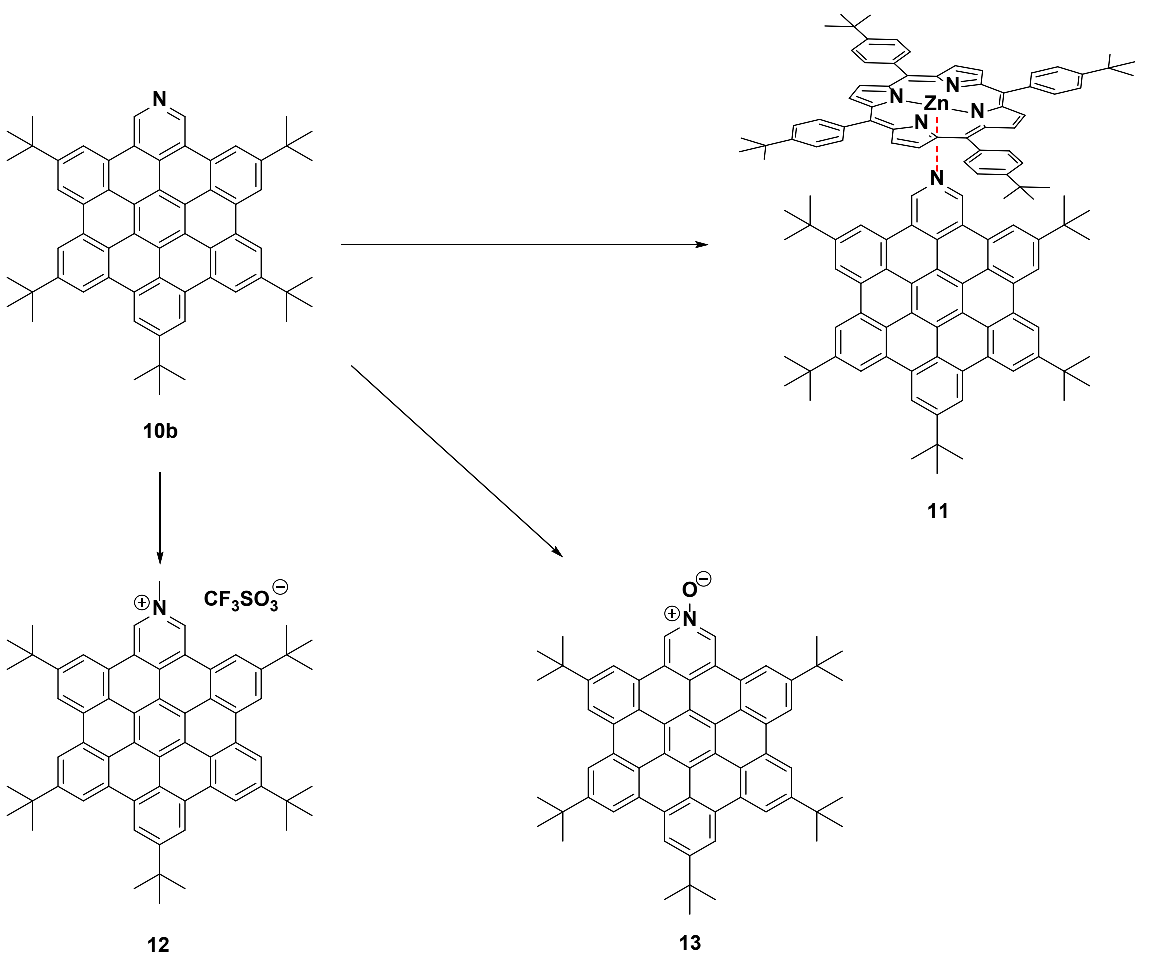

Scheme 5. Post-functionalization of **10a/b** to obtain derivatives **11**, **12** and **13**.

π-extended pyridinium triflate 12

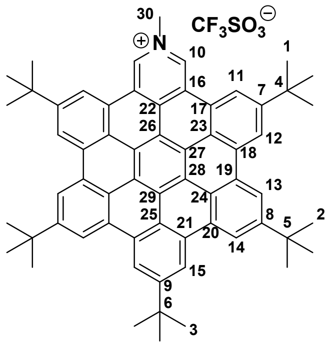

A Schlenk-tube (Ø 2.5 cm) equipped with a magnetic stirring bar was charged with **10b** (15.0 mg, 18.7 µmol), and the tube was put under nitrogen. MeI (1 mL) and acetonitrile (1 mL) (both were previously degassed by N_2 bubbling) were added, the tube was closed with a rubber septum and the reaction mixture was stirred for 2 h at rt. All solvents were removed under vacuum, the remaining yellow solid was re-dissolved in acetonitrile (5 mL). A solution of Ag(OTf) (10 mg, 39.0 µmol) in acetonitrile (also here acetonitrile previously degassed by N_2 bubbling was used for both cases) was added and the resulting mixture was stirred for 15 min at rt. The solvents were removed under vacuum and the remaining yellow solid was dissolved in CH_2Cl_2 (10 mL). The solution was washed with H_2O (2 *x 10 mL) and the aqueous phases were extracted with CH_2Cl_2 (2 x 10 mL). The combined organic phases were dried over $MgSO_4$ and the solvents were evaporated. The obtained solids were dissolved in a concentrated solution of CH_2Cl_2 and reprecipitated by addition of MeOH. The precipitate was filtered off through a glass-frit (P4) and dried under vacuum. The product was obtained as a yellow solid in a yield of 94 % (17.0 mg, 17.6 µmol).

^{1}H NMR ($CDCl_3$, 600 MHz, rt.): *δ* [ppm] = 9.87 (br, 2H, 10), 9.26 (s, 2H, 15), 9.20 (s, 2H, 14), 9.09 (br, 2H, 12), 9.04 (s, 2H, 13), 8.84 (br, 2H, 11), 4.77 (s, 3H, 30), 1.90 (s, 9H, 3), 1.80 (s, 18H, 2), 1.72 (s, 18H, 1).

^{13}C NMR ($CDCl_3$, 150 MHz, rt.): *δ* [ppm] = 151.5 (7), 151.2 (9), 150.4 (8), 135.2 (10), 131.2, 130.3, 130.3, 129.6, 128.1, 125.2, 124.1, 123.6, 122.9, 122.8, 122.2 (12), 122.0, 120.6, 120.4 (11), 120.0 (14), 119.6 (15), 119.5 (13), 114.7, 49.6 (30), 36.2 (4), 36.1 (6), 36.0 (5), 32.2 (3), 32.1 (2), 32.0 (1).

^{19}F NMR ($CDCl_3$, 470 MHz, rt.): *δ* [ppm] = 78.0 (s, CF_3)

MS (MALDI, DCTB): m/z (rel. int.) = 818 $[M]^+$ (100 %), 1786 $[2M\text{-}OTf]^+$ (7 %)

**HRMS (MALDI, DCTB)**:	m/z (calc. for $C_{62}H_{60}N$ $[M]^+$):	818.4720
	m/z (measured):	818.4723
	error [ppm]:	0.4

UV/Vis (MeOH, rt.) λ_{max} [nm] (ε [M^{-1} cm^{-1}]): 309 (38000), 325 (42000), 339 (61000), 376 (49000), 411 (26000), 488 (6300).

UV/Vis (toluene, rt.) λ_{max} [nm] (rel. int.): 313 (64%), 328 (71%), 343 (100%), 382 (88%), 413 (46%), 462 (21%), 493 (11%).

UV/Vis (THF, rt.) λ_{max} [nm] (rel. int.): 311 (57%), 325 (66%), 341 (100%), 378 (92%), 410 (44%), 455 (19%), 488 (11%)

UV/Vis (CH_2Cl_2, rt.) λ_{max} [nm] (rel. int.): 307 (61%), 326 (71%), 342 (100%), 361 (50%), 379 (59%), 427 (42%).

Emission (MeOH, rt., exc. 228 nm) λ_{max} [nm] (rel. Int.): 529 (100%).

Emission (toluene, rt., exc. 343 nm) λ_{max} [nm] (rel. Int.): 506 (100%), 541 (46%).

Emission (THF, rt., exc. 340 nm) λ_{max} [nm] (rel. Int.): 512 (100%).

Emission (CH_2Cl_2, rt., exc. 342 nm) λ_{max} [nm] (rel. Int.): 547 (100%).

π-extended pyridine N-oxide 13

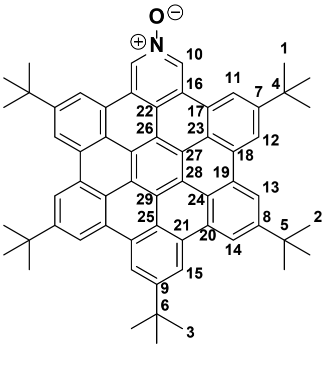

A Schlenk-tube (Ø 1.5 cm) equipped with a magnetic stirring bar was charged with **10b** (50.0 mg, 62.0 µmol), and $CHCl_3$ (1 mL). mCPBA (70 %) (15.0 mg, 62.0 µmol) was added at 0 °C and the solution was stirred for 24 h. During that time it was allowed to warm to rt. Sat. aq. $NaHCO_3$ (2 mL) was added and the mixture was stirred vigorously for 30 min. CH_2Cl_2 (10 mL) and H_2O (10 mL) were added, the phases were separated and the aqueous phase was extracted with CH_2Cl_2 (10 mL). The combined organic phases were washed with sat. aq. $NaHCO_3$ (20 mL) and brine (20 mL). The solvents were evaporated, and the remaining solids were dissolved in a minimal amount of CH_2Cl_2 and reprecipitated by addition of MeOH. The precipitate was filtered off through a glass-frit (P4) and dried under vacuum. The product was obtained as a yellow powder in a yield of 93 % (47.0 mg, 57.0 µmol).

^{1}H NMR ($CDCl_3$, 400 MHz, rt.): *δ* [ppm] = 9.24 (br, 2H, 10), 9.20 (s, 2H, 15), 9.15 (s, 2H, 14), 8.99 (s, 2H, 13), 8.94 (s, 2H, 12), 8.49 (s, 2H, 11), 1.95 (s, 9H, 3), 1.89 (s, 18H, 2), 1.76 (s, 18H, 1).

^{13}C NMR ($CDCl_3$, 100 MHz, rt.): *δ* [ppm] = 149.6 (9), 149.2 (7), 149.2 (8), 131.2 (10), 130.4, 130.1, 130.0, 129.7, 125.5, 123.5, 123.0, 120.3, 120.0, 119.1 (14), 119.1 (15), 119.0 (11), 118.9 (13), 36.1 (6), 36.0 (5), 35.8 (4), 32.4 (3), 32.3 (2), 32.2 (1).

MS (MALDI, DCTB): m/z (rel. int.) = 819 $[M]^+$ (100 %), 803 $[M\text{-}O]^+$ (30 %)

**HRMS (MALDI, DCTB)**:	m/z (calc. for $C_{61}H_{57}NO$ $[M]^+$):	819.4435
	m/z (measured):	819.4427
	error [ppm]:	1.0

UV/Vis (toluene, rt.) λ_{max} [nm] (ε [M^{-1} cm^{-1}]): 335 (25000), 350 (60000), 366 (138000), 410 (31000), 456 (2800), 484 (1900).

Emission (toluene, rt., exc. 366 nm) λ_{max} [nm] (rel. Int.): 466 (54%), 487 (100%), 497 (34%), 508 (22%), 520 (40%), 558 (8%).

***π*-extended pyridine Zn-porphyrin complex 11**

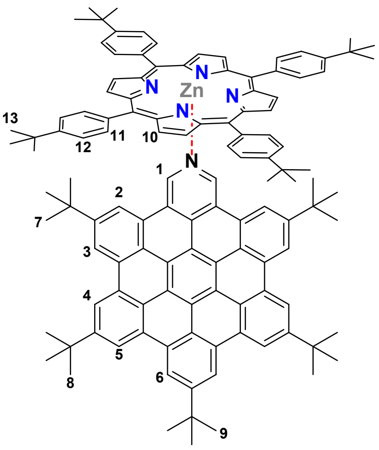

Pyridine-HBC **10b** (1.00 mg, 1.24 µmol) and Zn-porphyrin (1.12 mg, 1.24 µmol) were dissolved in anhydrous C_6D_6 (520 µL) in an NMR-tube and a dark purple solution was obtained. The quantitative complex formation in solution was confirmed by ^{1}H NMR spectroscopy.

^{1}H NMR (C_6D_6, 400 MHz, rt.): *δ* [ppm] = 9.44 (s, 8H, 10), 9.40 (s, 2H, 6), 9.39 (s, 2H, 5), 9.36 (s, 2H, 4), 9.30 (s, 2H, 3), 8.45 (d, ^3J = 8.4 Hz, 8H, 12), 7.79 (s, 2H, 2), 7.63 (d, ^3J = 8.3 Hz, 8H, 11), 4.61 (s, 2H, 1), 1.71 (s, 18H, 8), 1.63 (s, 27H, 7+9), 1.46 (s, 36H, 13).

2.6 Reference compound Pentakis-*tert*-butyl HBC 14

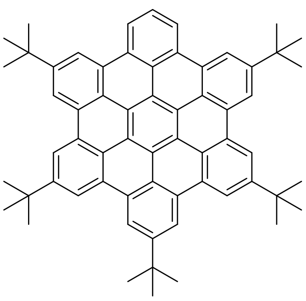

Reference compound **14** was synthesized according to literature procedures[S4].

3 Photophysical characterization

3.1 Steady state absorption data of the plain compounds

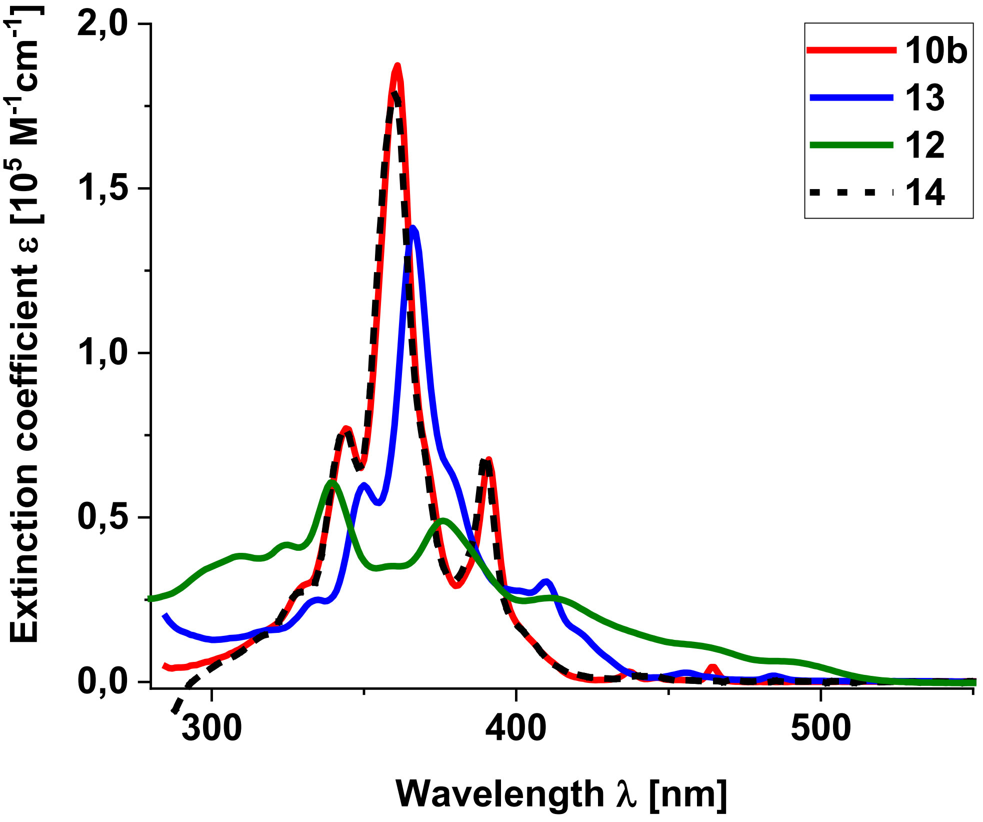

Figure 1. Steady state absorption for compounds **10b**, **13** and **14** (toluene, rt.) and **12** (MeOH, rt.)

Table 1. Peaks and extinction coefficients found in the steady state absorption of compounds **10b**, **13** and **14** (toluene, rt.) and **12** (MeOH, rt.).

10b		14		13		12	
λ [nm]	ε [$M^{-1}cm^{1}$]	λ [nm]	ε [$M^{-1}cm^{1}$]	λ [nm]	ε [$M^{-1}cm^{1}$]	λ [nm]	ε [$M^{-1}cm^{1}$]
~331(s)	~30000	~329	~27000	335	25000	309	38000
344	77000	344	77000	350	60000	325	42000
361	187000	360	179000	366	138000	339	61000
~370(s)	~72000	~369	~75000	~378(s)	~65000	376	49000
391	68000	390	69000	410	31000	411	26000
437	3100	440	1900	~419(s)	~16000	~459(s)	~11000
446	1300	445	2000	456	2800	488	6300
464	4500			484	1900		

3.2 Steady state absorption data of the compounds with an excess of TFA

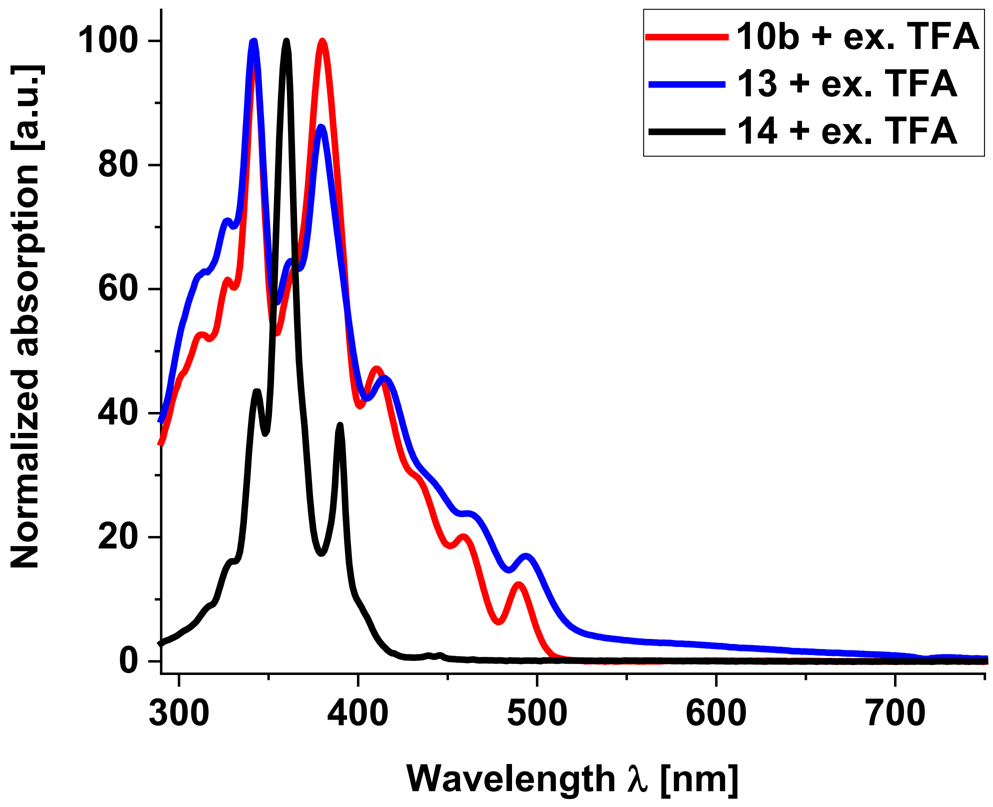

Figure 2. Normalized steady state absorption data for **10b, 13 and 14** each with an excess of TFA (toluene, rt.)

Table 2. Peaks and relative intensities found in the normalized steady state absorption of compounds **10b**, **13** and **14** each with an excess of TFA (toluene, rt.).

**10b**		**14**		**13**	
λ [nm]	rel. int. [%]	λ [nm]	rel. int. [%]	λ [nm]	rel. int. [%]
312	53	~329(s)	16	~312(s)	62
327	62	344	44	327	71
342	98	360	100	342	100
~362(s)	62	~369(s)	41	362	65
380	100	390	38	379	86
410	47	439	1	415	46
~434(s)	29	446	1	~460(s)	24
459	20			494	17
490	12				

3.3 Steady state absorption data of 12 in different solvents

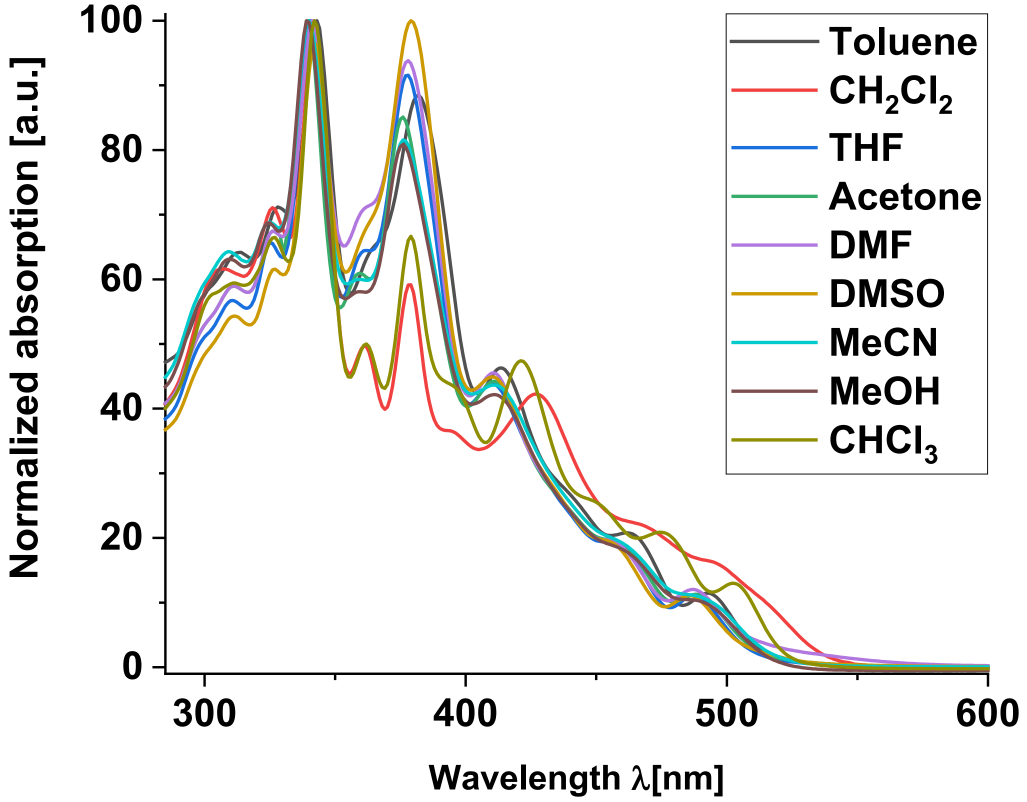

Figure 3. Normalized steady state absorption data for **12** in different solvents indicating the solvatochromic behavior of **12**.

Table 3. Peaks and relative intensities found in the normalized steady state absorption of compound **12** in different solvents.

Toluene		CH_2Cl_2		THF		Acetone		DMF	
λ [nm]	rel. int. [%]	λ [nm]	rel. int. [%]	λ [nm]	rel. int. [%]	λ [nm]	rel. int. [%]	λ [nm]	rel. int. [%]
313	64	307	61	311	57	311	59	311	59
328	71	326	71	325	66	326	67	326	67
343	100	342	100	341	100	339	100	341	100
~365(s)	~65	361	50	~361(s)	~64	359	61	~361(s)	~71
~382(s)	~88	379	59	378	92	376	85	378	94
413	46	~395(s)	~36	410	44	411	44	411	45
462	21	427	42	455	19	456	20	455	20
493	11	~365(s)	~23	488	11	488	11	487	12
		~497(s)	~16						

Table 4. Peaks and relative intensities found in the normalized steady state absorption of compound **12** in different solvents (continued).

**DMSO**		**MeCN**		**MeOH**		**$CHCl_3$**	
λ [nm]	rel. int. [%]	λ [nm]	rel. int. [%]	λ [nm]	rel. int. [%]	λ [nm]	rel. int. [%]
311	54	309	64	309	63	311	59
326	62	325	69	324	69	326	66
342	97	340	100	339	100	342	100
~362(s)	~68	~360(s)	~60	~360(s)	~58	362	50
379	100	376	81	376	81	379	67
410	45	411	44	411	42	~395(s)	~44
~453(s)	~20	~457(s)	~20	~458(s)	19	421	47
486	11	487	11	488	10	~448(s)	~25
						474	21
						503	13

3.4 Steady state emission data of the plain compounds

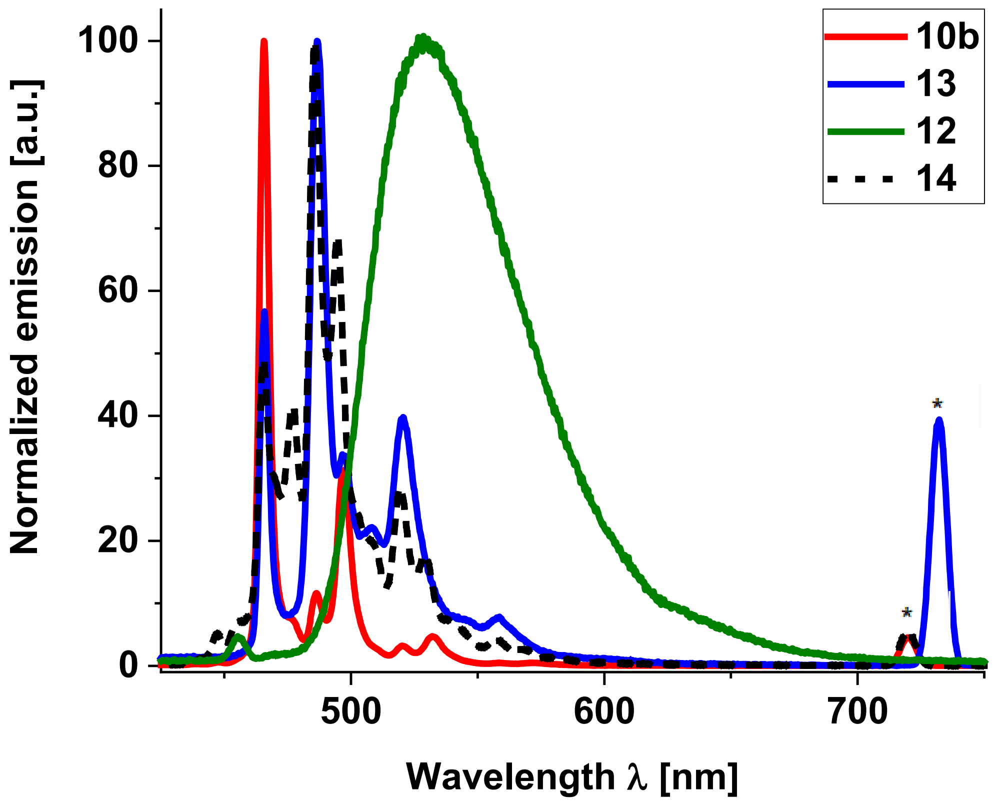

Figure 4. Normalized steady state emission for compounds **10b**, **13** and **14** (toluene, rt.) and **12** (MeOH, rt.). * Artifacts of 2nd order scattering at the double excitation wavelength.

Table 5. Peaks and relative intensities found in the steady state emission of compounds **10b**, **13** and **14** (toluene, rt.) and **12** (MeOH, rt.).

10b		14		13		12	
λ [nm]	rel. int. [%]	λ [nm]	rel. int. [%]	λ [nm]	rel. int. [%]	λ [nm]	rel. int. [%]
466	100	447	5	466	54	529	100
~477(s)	~8	456	7	487	100		
486	12	466	49	497	34		
496	32	477	42	508	22		
520	3	486	100	520	40		
531	5	495	69	558	8		
		~501(s)	~26				
		~508(s)	~20				
		520	29				
		529	18				
		~539(s)	~7				
		557	4				
		568	3				

3.5 Steady state emission data of the compounds with an excess of TFA

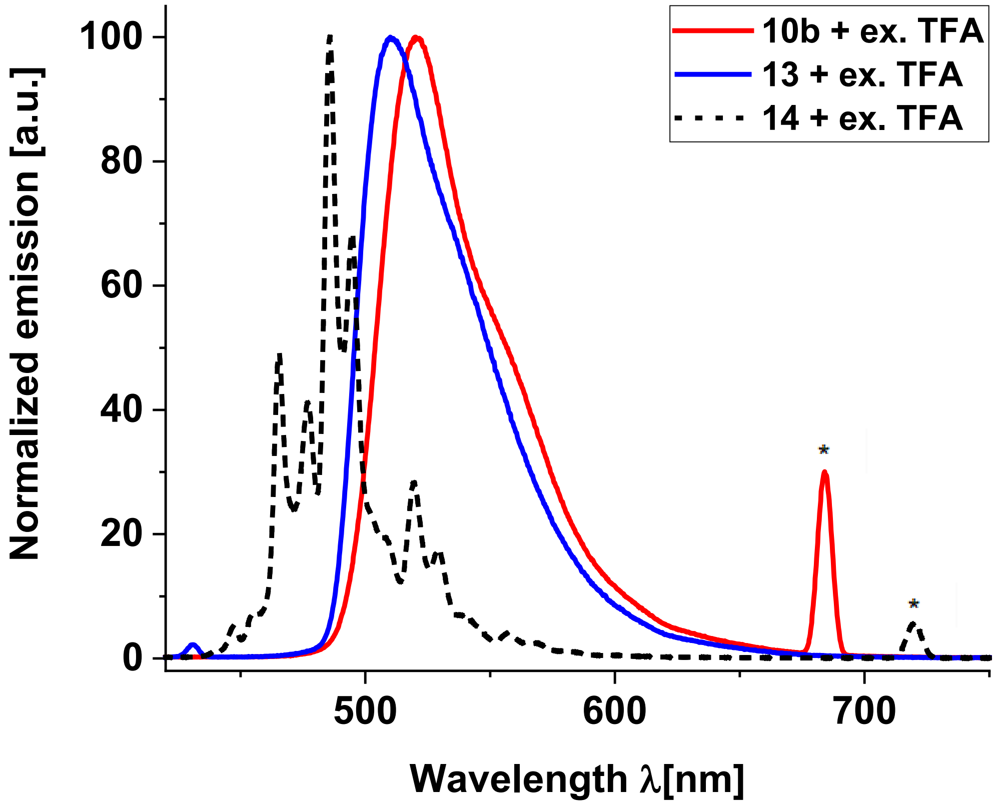

Figure 5. Normalized steady state emission data for **10b**, **13** and **14** each with an excess of TFA (toluene, rt.). * Artifacts of 2nd order scattering at the double excitation wavelength.

Table 6. Peaks and relative intensities found in the normalized steady state emission of compounds **10b**, **13** and **14** each with an excess of TFA (toluene, rt.).

10b		14		13	
λ [nm]	rel. int. [%]	λ [nm]	rel. int. [%]	λ [nm]	rel. int. [%]
520	100	447	5	511	100
		456	7		
		465	49		
		477	41		
		486	100		
		495	69		
		~502(s)	~24		
		~508(s)	~20		
		519	28		
		529	18		
		~540(s)	~7		
		557	4		
		568	2		

3.6 Steady state emission data of 12 in different solvents

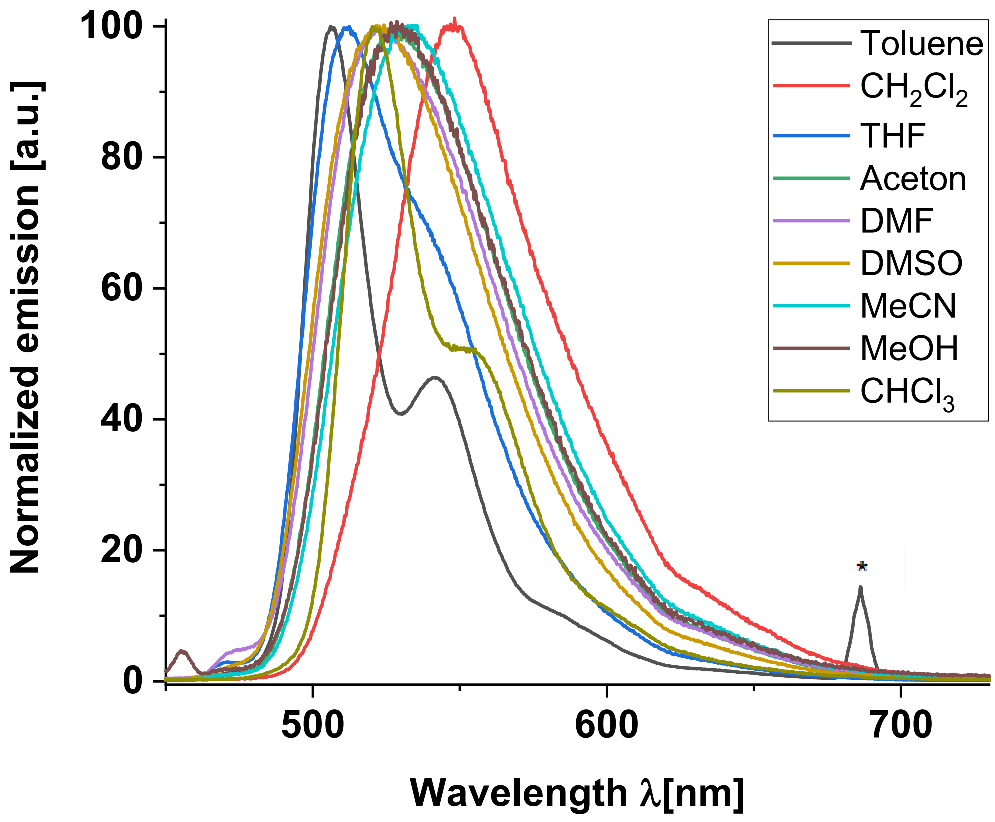

Figure 6. Normalized steady state emission data for **12** in different solvents indicating the solvatochromic behavior of **12**. * Artifacts of 2nd order scattering at the double excitation wavelength.

Table 7. Peaks and relative intensities found in the normalized steady state emission of compound **12** in different solvents.

Toluene		CH_2Cl_2		THF		Acetone		DMF	
λ [nm]	rel. int. [%]	λ [nm]	rel. int. [%]	λ [nm]	rel. int. [%]	λ [nm]	rel. int. [%]	λ [nm]	rel. int. [%]
506	100	547	100	512	100	528	100	522	100
541	46			~535(s)	72				

Table 8. Peaks and relative intensities found in the normalized steady state emission of compound **12** in different solvents (continued).

DMSO		MeCN		MeOH		$CHCl_3$	
λ [nm]	rel. int. [%]	λ [nm]	rel. int. [%]	λ [nm]	rel. int. [%]	λ [nm]	rel. int. [%]
522	100	533	100	529	100	521	100
						~551(s)	51

4 X-ray data for 12

Single clear light-yellow block crystals of **12** were obtained from a concentrated solution in $CHCl_3$ by slow evaporation. A suitable crystal with dimensions 0.34 × 0.20 × 0.10 mm^3 was selected and mounted on a mylar loop in perfluoroether oil on a SuperNova, Dual, Cu at home/near, Atlas diffractometer. The crystal was kept at a steady T = 153.00(10) K during data collection. The structure was solved with the **ShelXT**[S5] solution program using dual methods and by using **Olex2**[S6] as the graphical interface. The model was refined with **ShelXL**[S7] using full matrix least squares minimization on $\boldsymbol{F^2}$.

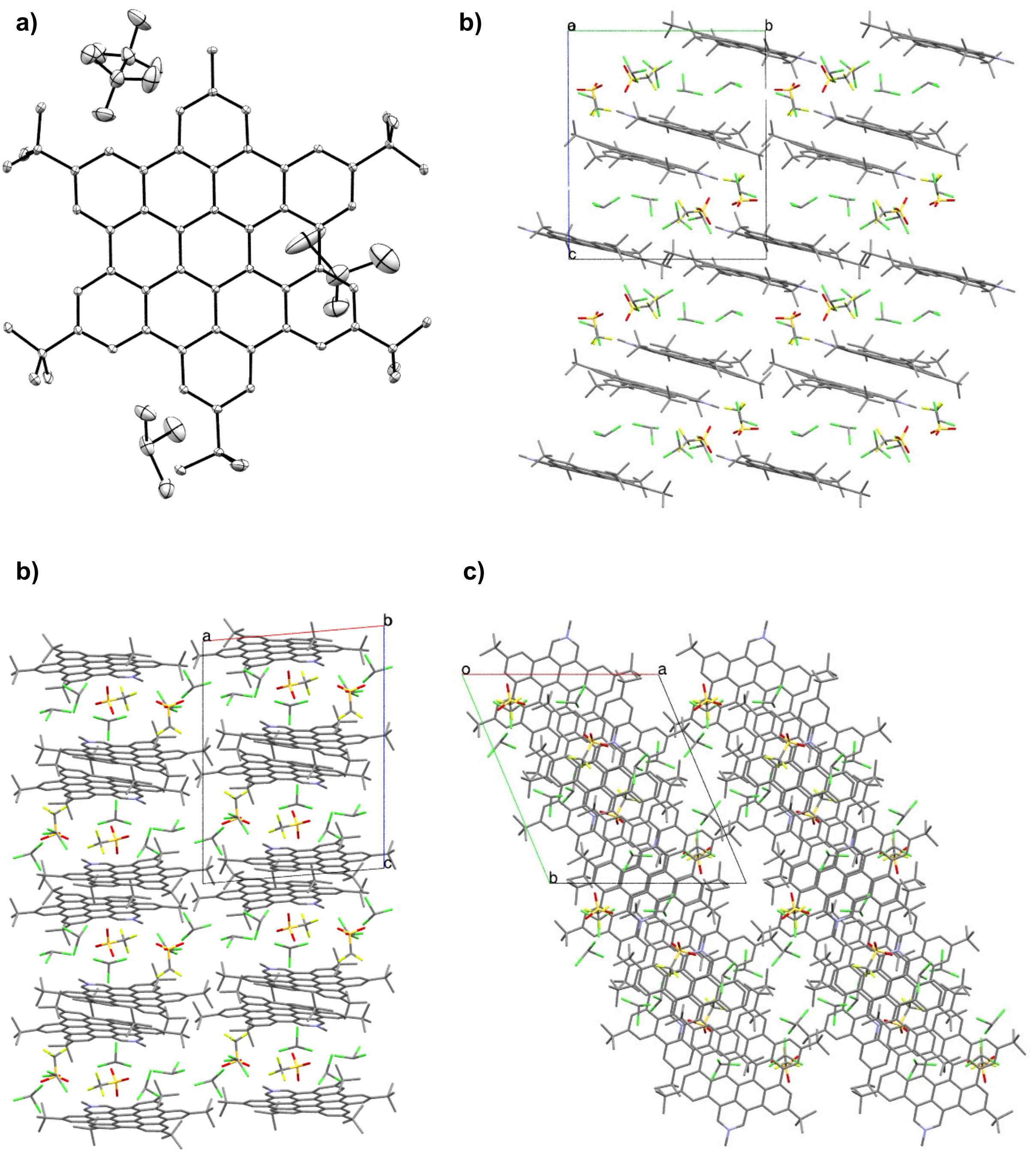

Figure 7. a) ORTEP drawing of **12** with counterion ($CF_3SO_3^-$) and 2 molecules of solvent ($CHCl_3$). Ellipsoids are drawn at 30% probability level; b) Packing with view along crystallographic a axis; c) Packing with view along crystallographic b axis; d) Packing with view along crystallographic c axis. Hydrogens are omitted for clarity.

Table 9. Crystal data and structure refinement for 12.

Identification code	20Jux_KS01
Empirical formula	$C_{78}Cl_{45}F_3H_{75}NO_3S$
Formula weight	1266.60
Temperature/K	153.00(10)
Crystal system	triclinic
Space group	P-1
a/Å	17.6433(5)
b/Å	20.2636(8)
c/Å	21.6900(8)
α/°	87.651(3)
β/°	84.593(3)
γ/°	67.078(3)
Volume/Å^3	7110.4(5)
Z	4
ρ_{calc}g/cm^3	1.183
μ/mm^{-1}	3.391
F(000)	2628.0
Crystal size/mm^3	0.336 × 0.202 × 0.105
Radiation	Cu Kα (λ = 1.54184)
2Θ range for data collection/°	6.246 to 127.83
Index ranges	$-20 \leq h \leq 10$, $-23 \leq k \leq 22$, $-25 \leq l \leq 24$
Reflections collected	40250
Independent reflections	22603 [R_{int} = 0.0427, R_{sigma} = 0.0422]
Data/restraints/parameters	22603/0/1496
Goodness-of-fit on F^2	1.036
Final R indexes [I>=2σ (I)]	R_1 = 0.0760, wR_2 = 0.2125
Final R indexes [all data]	R_1 = 0.0897, wR_2 = 0.2299
Largest diff. peak/hole / e Å^{-3}	0.96/-1.03

5 NMR spectroscopy and mass spectrometry data

5.1 Spectra for the synthesis of the "lower-half" precursors 4a/b

3,4,5-Tri-(4-*tert*-butylphenyl)-bromobenzene 3

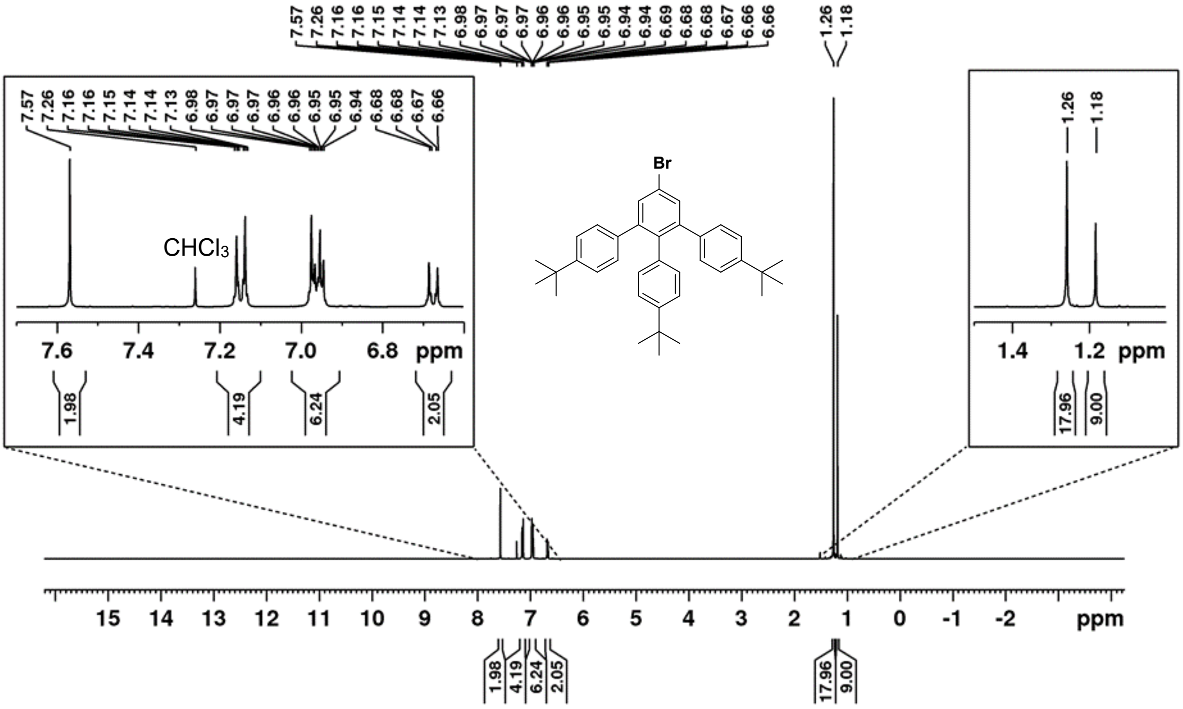

Figure 8. ^{1}H NMR spectrum of **3** (400 MHz, $CDCl_3$, rt.).

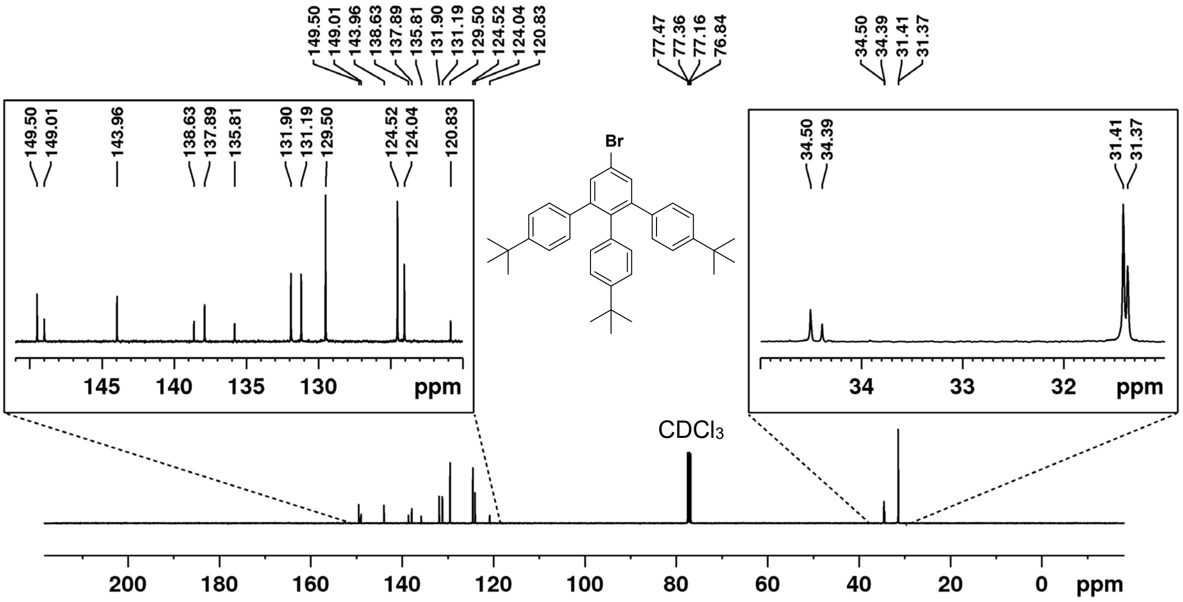

Figure 9. ^{13}C NMR spectrum of **3** (100 MHz, $CDCl_3$, rt.).

Mass Spectrum SmartFormula Report

Analysis Info			
Analysis Name	D:\Data\2018\Jux-2018\Schoell-ks58-.d	Acquisition Date	8/8/2018 10:40:47 AM
Method	tune_low-APPI.m	Operator	MD
Sample Name	Low Concentration Tunemix	Instrument	maXis 288882.20183
Comment	CH2Cl2-ACN		

Acquisition Parameter

Source Type	APPI	Ion Polarity	Positive	Set Nebulizer	2.5 Bar
Focus	Not active	Set Capillary	700 V	Set Dry Heater	220 °C
Scan Begin	50 m/z	Set End Plate Offset	-500 V	Set Dry Gas	1.5 l/min
Scan End	1600 m/z	Set Charging Voltage	0 V	Set Divert Valve	Waste
		Set Corona	0 nA	Set APCI Heater	250 °C

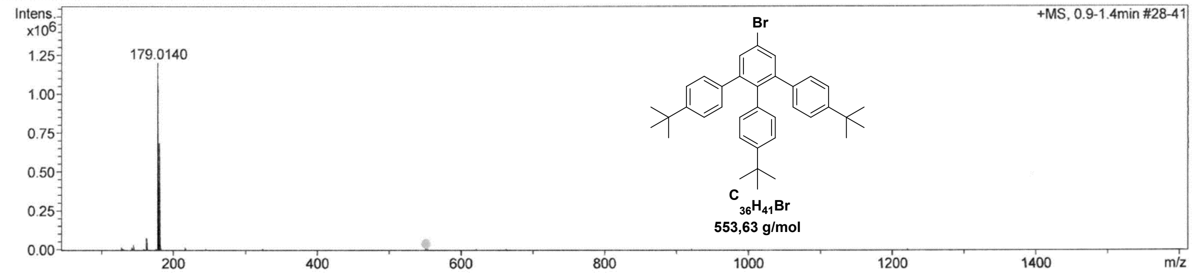

Meas. m/z	#	Ion Formula	m/z	err [ppm]	mSigma	# mSigma	Score	rdb	e⁻ Conf	N-Rule
552.2399	1	C36H41Br	552.2386	-2.2	112.2	2	6.09	16.0	odd	ok
	2	C36H40Br	551.2308	1.$	714.7	3	0.00	16.5	even	ok

Figure 10. HRMS (APPI, CH_2Cl_2/Acetonitrile) of 3.

Display Report

Analysis Info			
Analysis Name	D:\Data\2018\Jux-2018\Schoell-ks58-.d	Acquisition Date	8/8/2018 10:40:47 AM
Method	tune_low-APPI.m	Operator	MD
Sample Name	Low Concentration Tunemix	Instrument	maXis 288882.20183
Comment	CH2Cl2-ACN		

Acquisition Parameter

Source Type	APPI	Ion Polarity	Positive	Set Nebulizer	2.5 Bar
Focus	Not active	Set Capillary	700 V	Set Dry Heater	220 °C
Scan Begin	50 m/z	Set End Plate Offset	-500 V	Set Dry Gas	1.5 l/min
Scan End	1600 m/z	Set Charging Voltage	0 V	Set Divert Valve	Waste
		Set Corona	0 nA	Set APCI Heater	250 °C

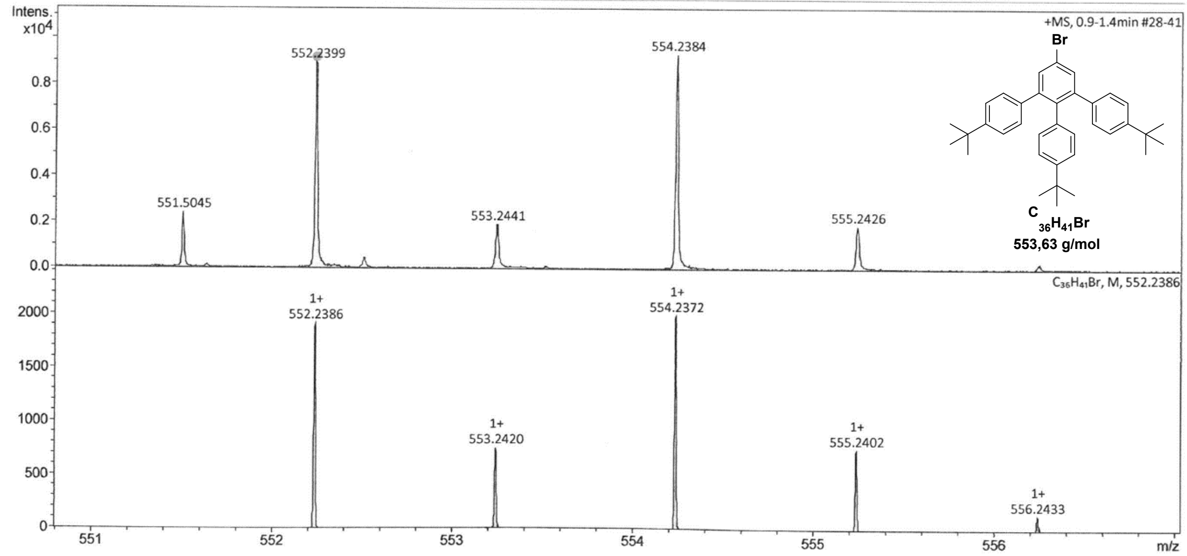

Figure 11. HRMS (APPI, CH_2Cl_2/Acetonitrile) of 3 (enlargement of the corresponding signal of **3**, top: measured, bottom: calculated).

3,4,5-Tri-(4-*tert*-butylphenyl)phenylboronic acid 4a

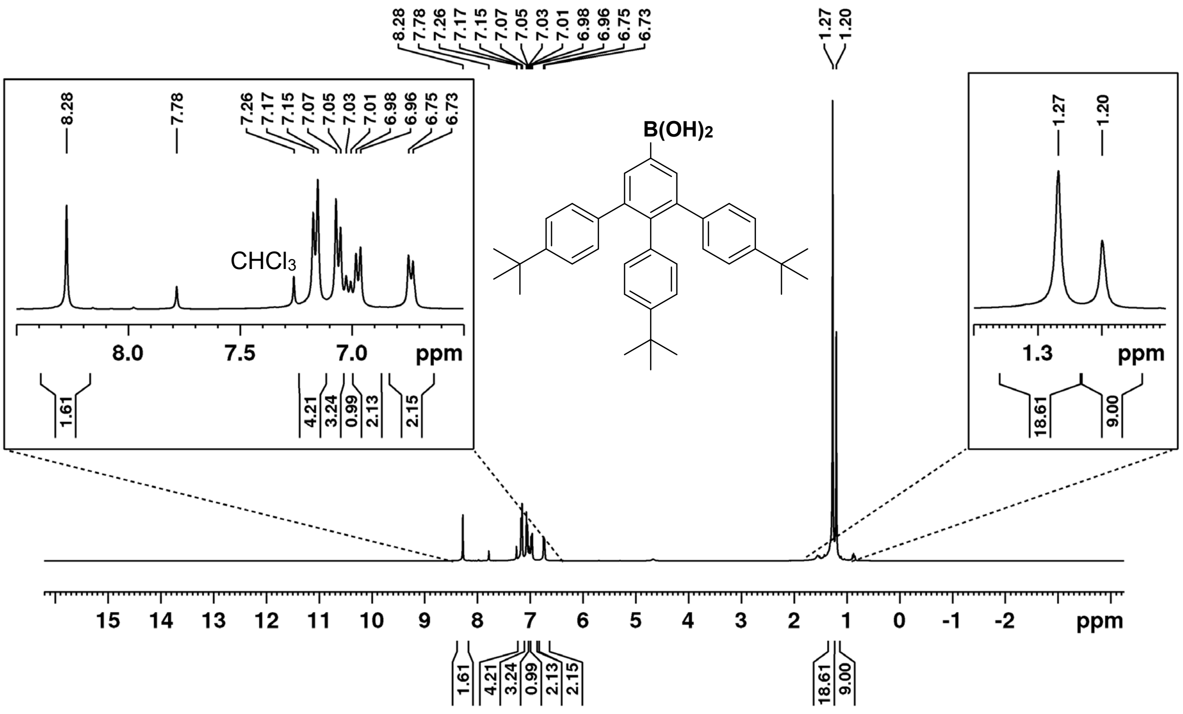

Figure 12. ^{1}H NMR spectrum of **4a** (400 MHz, $CDCl_3$, rt.).

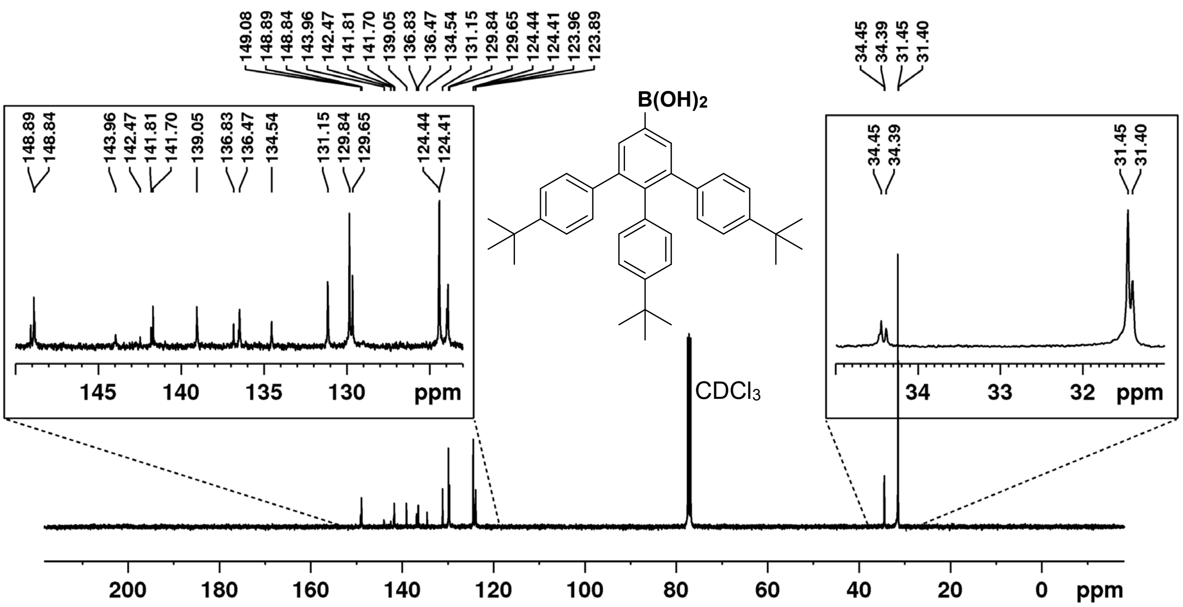

Figure 13. ^{13}C NMR spectrum of **4a** (100 MHz, $CDCl_3$, rt.).

3,4,5-Tri-(4-*tert*-butylphenyl)phenylboronic acid pinacol ester 4b

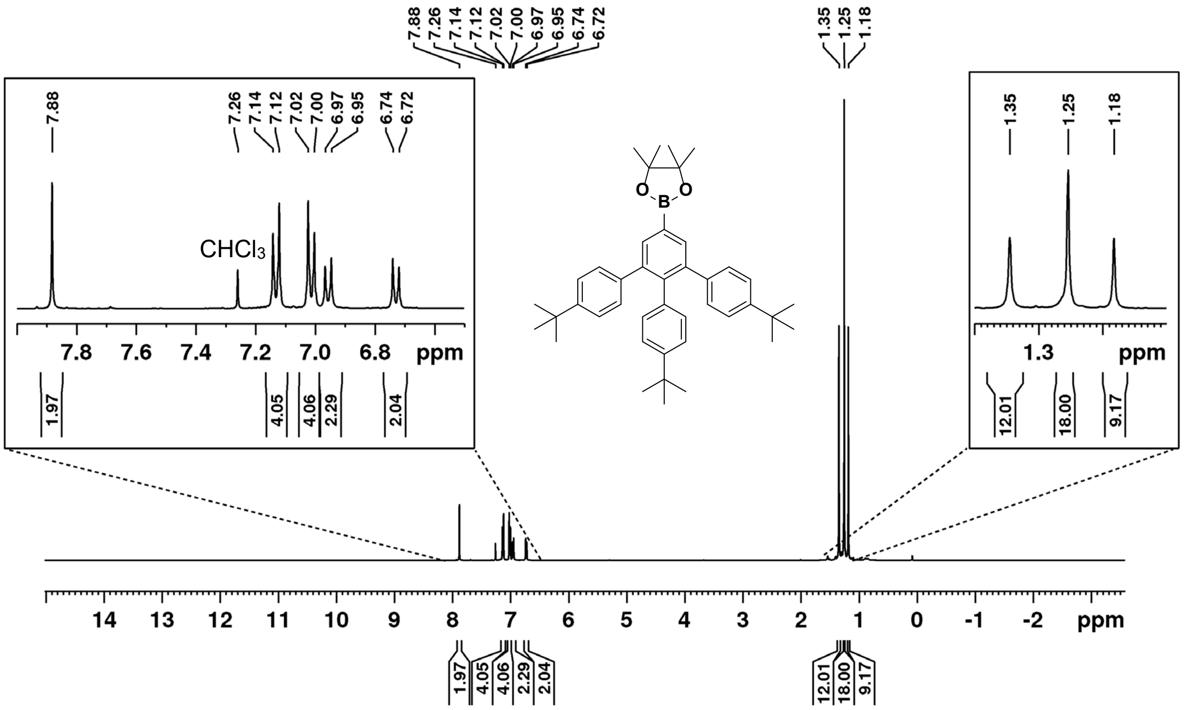

Figure 14. ^1H NMR spectrum of **4b** (400 MHz, $CDCl_3$, rt.).

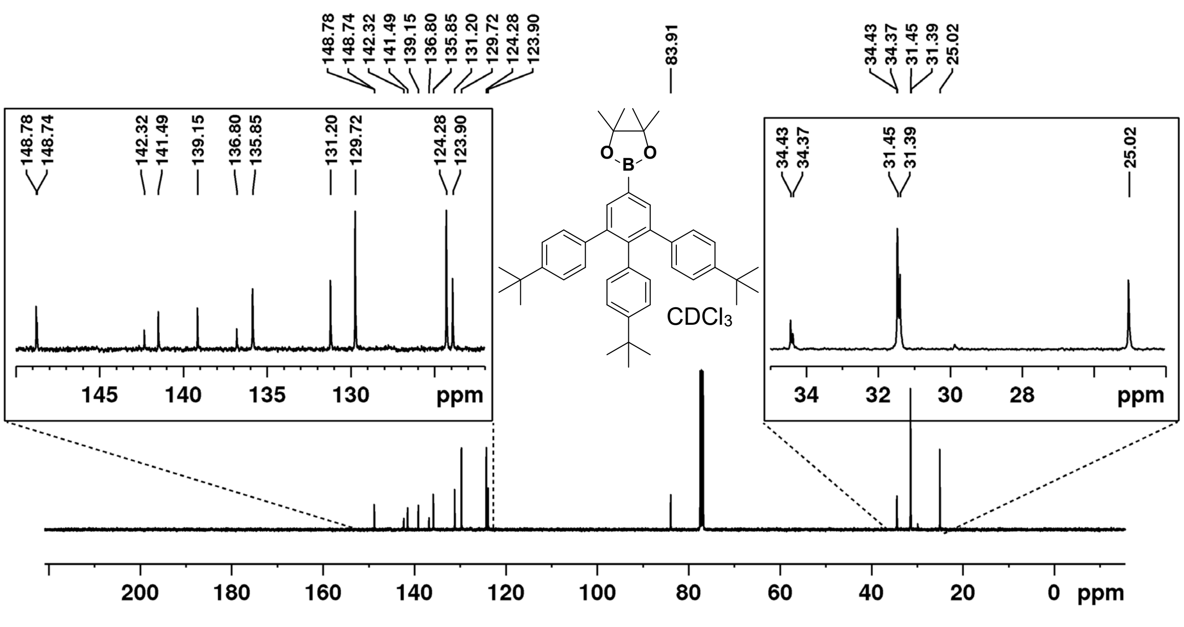

Figure 15. ^{13}C NMR spectrum of **4b** (100 MHz, $CDCl_3$, rt.).

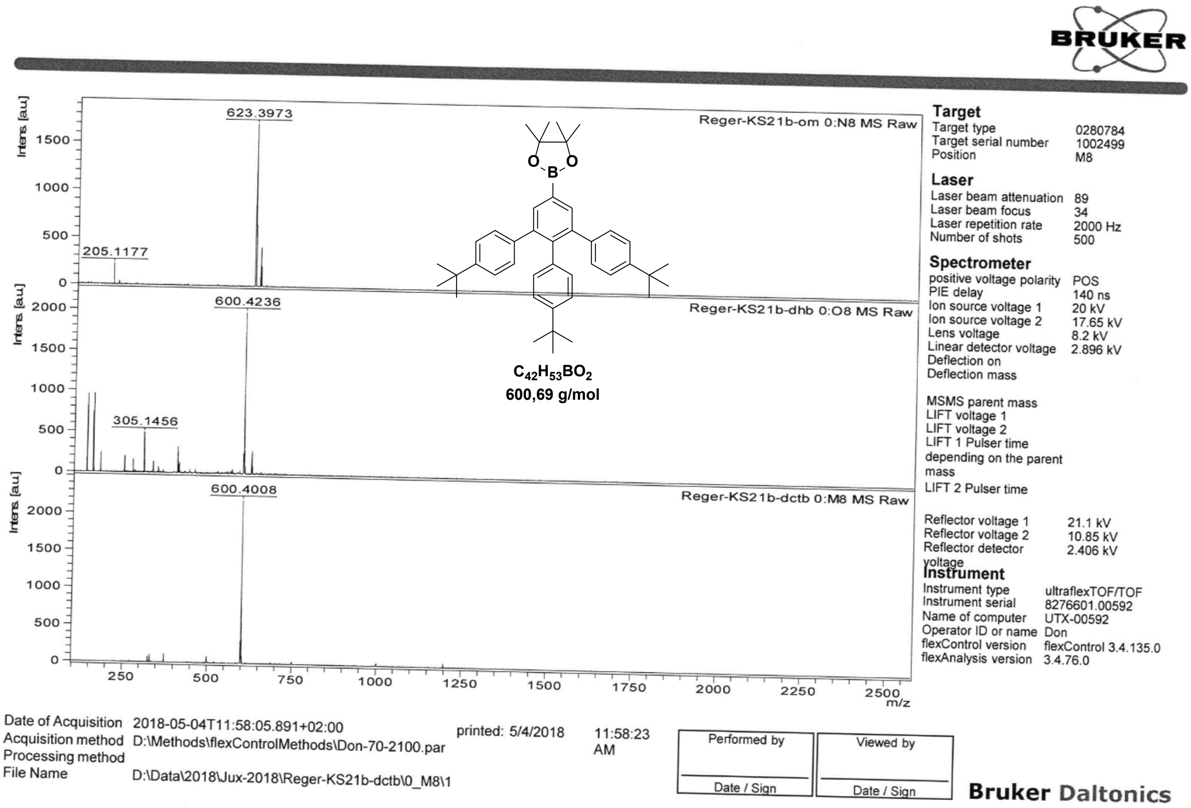

Figure 16. MS (MALDI, top: without matrix, middle: DHB, bottom: DCTB) of **4b**.

Formula	Mass	Error	mSigma	DblEq	N rule	Electron Configuration
C 42 H 53 B O 2	600.4133	1.3820	53.0947	17.00	ok	odd
C 42 H 53 B N O	598.4215	26.4557	585.3120	17.50	ok	even

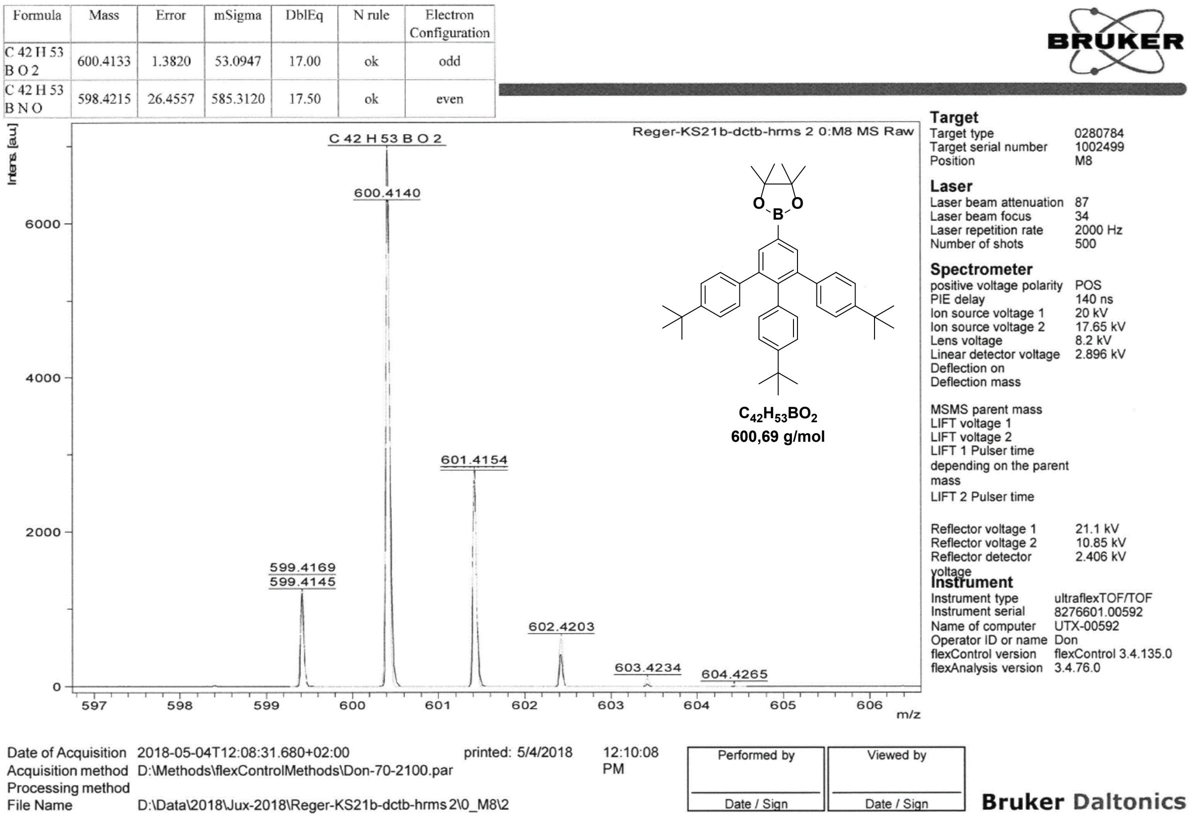

Figure 17. HRMS (MALDI, DCTB) of **4b.** Measured (black) and calculated (grey) spectra overlaid.

5.2 Spectra for the synthesis of the "top-half" precursors 8a/b

3,5-diphenyl-4-amiopyridine 7a

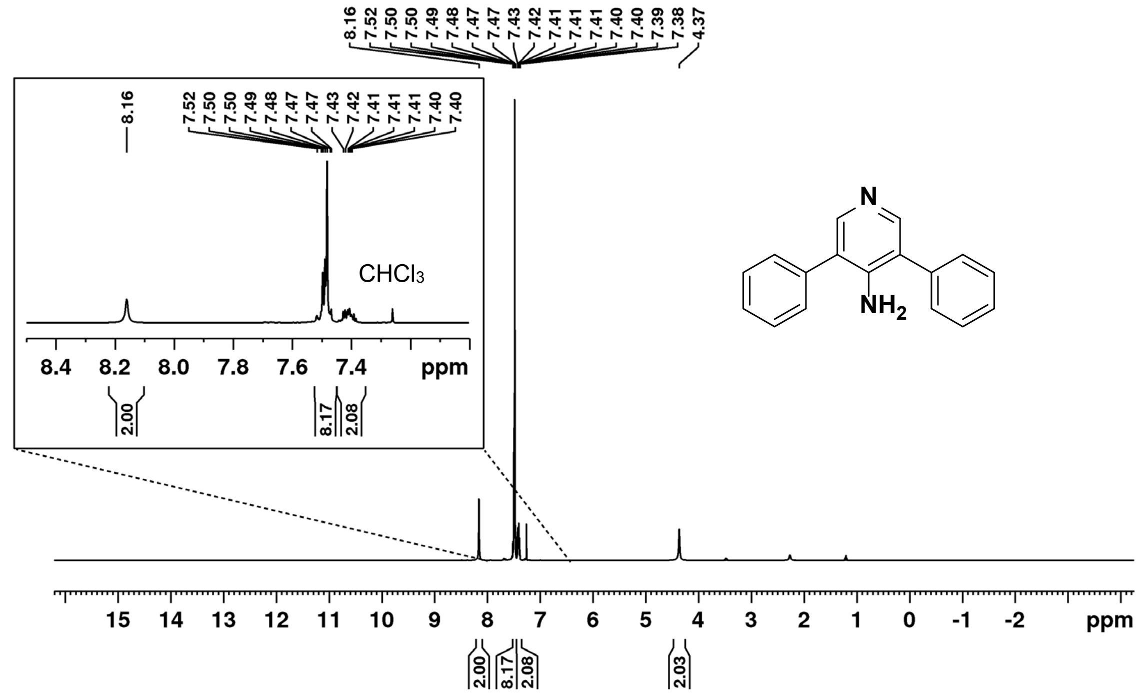

Figure 18. ^{1}H NMR spectrum of **7a** (400 MHz, $CDCl_3$, rt.).

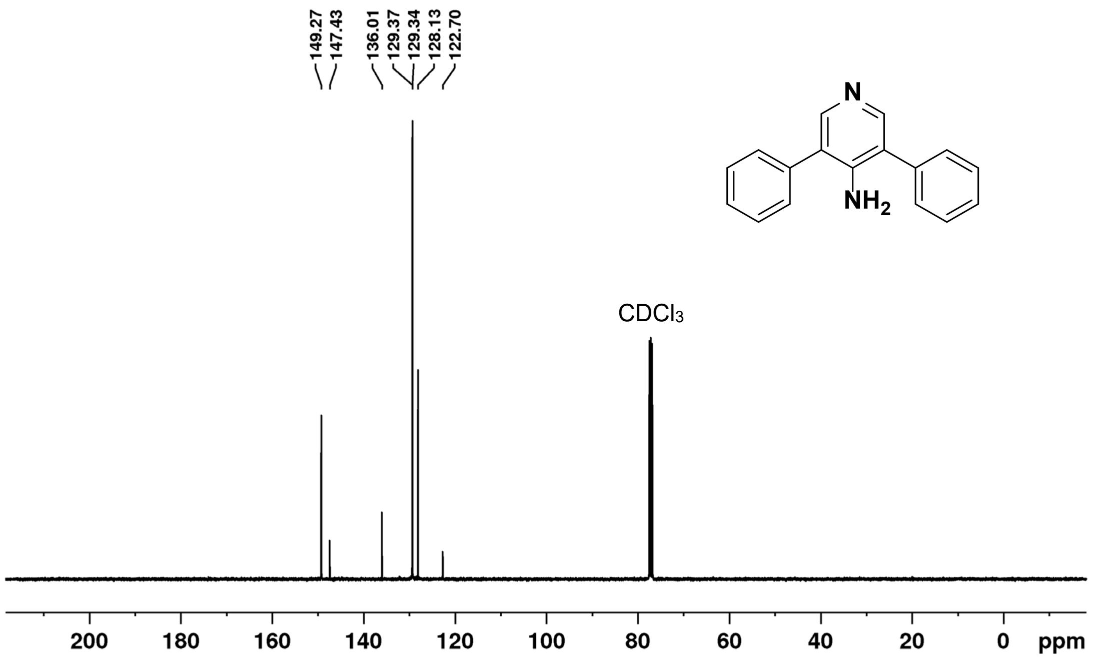

Figure 19. ^{13}C NMR spectrum of **7a** (100 MHz, $CDCl_3$, rt.).

3,5-diphenyl-4-bromopyridine 8a

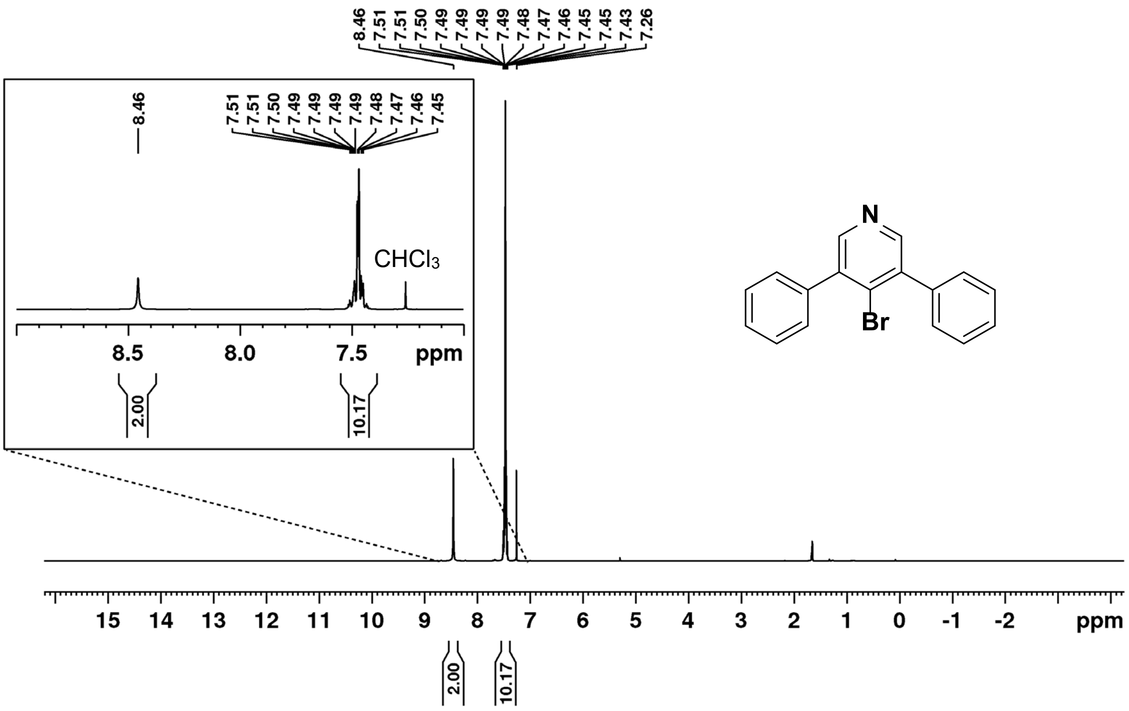

Figure 20. ^{1}H NMR spectrum of **8a** (400 MHz, $CDCl_3$, rt.).

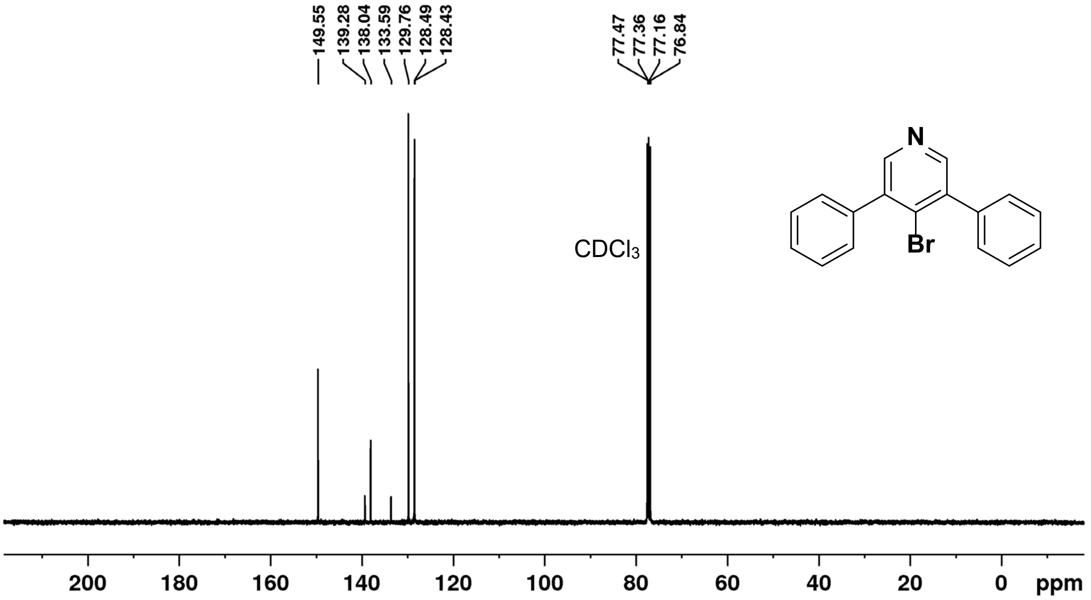

Figure 21. ^{13}C NMR spectrum of **8a** (100 MHz, $CDCl_3$, rt.).

Mass Spectrum SmartFormula Report

Analysis Info

Analysis Name	D:\Data\2018\Jux-2018\Reger-KS-19--appi-.d	Acquisition Date	5/4/2018 11:46:24 AM
Method	tune_low-APPI.m	Operator	MD
Sample Name		Instrument	maXis 288882.20183
Comment	CH2Cl2-,ACN		

Acquisition Parameter

Source Type	APPI	Ion Polarity	Positive	Set Nebulizer	2.5 Bar
Focus	Not active	Set Capillary	700 V	Set Dry Heater	220 °C
Scan Begin	50 m/z	Set End Plate Offset	-500 V	Set Dry Gas	1.5 l/min
Scan End	1550 m/z	Set Charging Voltage	0 V	Set Divert Valve	Waste
		Set Corona	0 nA	Set APCI Heater	250 °C

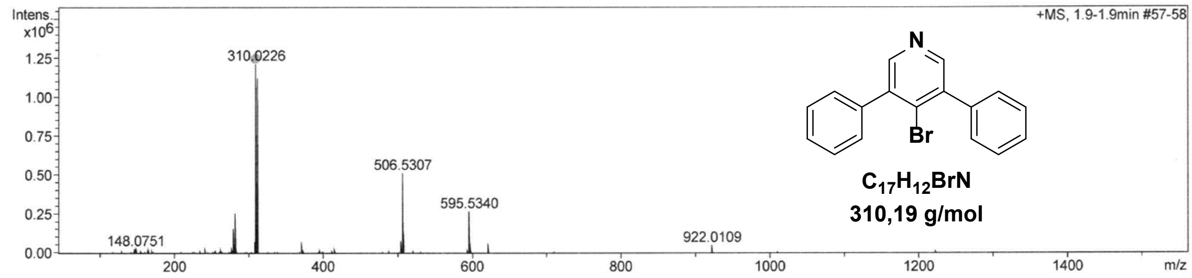

Meas. m/z	#	Ion Formula	m/z	err [ppm]	mSigma	# mSigma	Score	rdb	e⁻ Conf	N-Rule
310.0226	1	C17H13BrN	310.0226	0.0	53.8	1	100.00	11.5	even	ok

Figure 22. HRMS (APPI, CH_2Cl_2/Acetonitrile) of **8a**.

Display Report

Analysis Info

Analysis Name	D:\Data\2018\Jux-2018\Reger-KS-19--appi-.d	Acquisition Date	5/4/2018 11:46:24 AM
Method	tune_low-APPI.m	Operator	MD
Sample Name		Instrument	maXis 288882.20183
Comment	CH2Cl2-,ACN		

Acquisition Parameter

Source Type	APPI	Ion Polarity	Positive	Set Nebulizer	2.5 Bar
Focus	Not active	Set Capillary	700 V	Set Dry Heater	220 °C
Scan Begin	50 m/z	Set End Plate Offset	-500 V	Set Dry Gas	1.5 l/min
Scan End	1550 m/z	Set Charging Voltage	0 V	Set Divert Valve	Waste
		Set Corona	0 nA	Set APCI Heater	250 °C

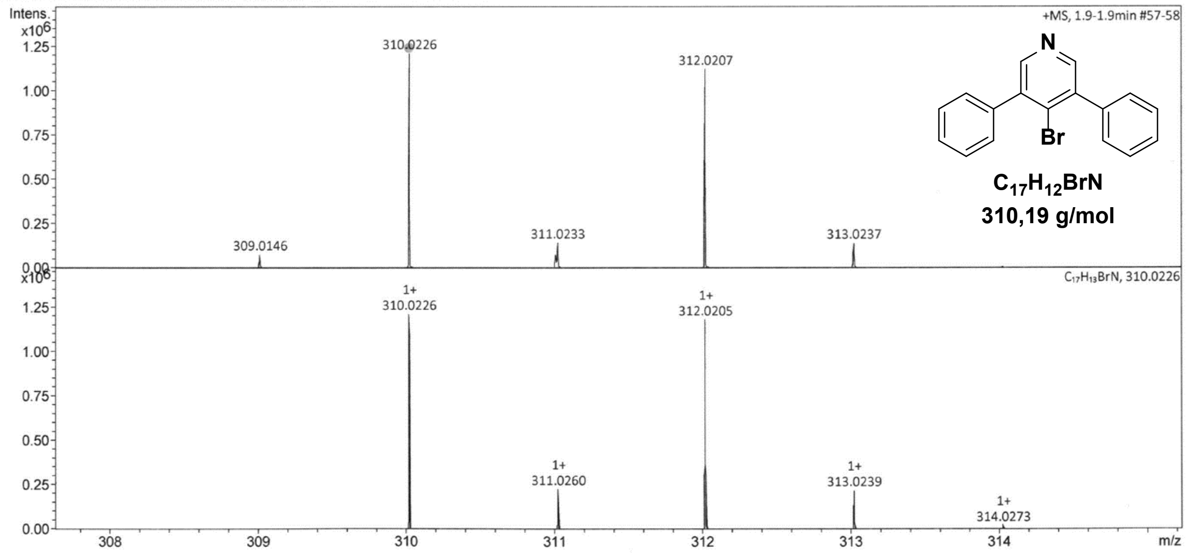

Figure 23. HRMS (APPI, CH_2Cl_2/Acetonitrile) of **8a** (enlargement of the corresponding signal of **8a**, top: measured, bottom: calculated).

3,5-di-(3-*tert*-butyl-phenyl)-4-aminopyridine 7b

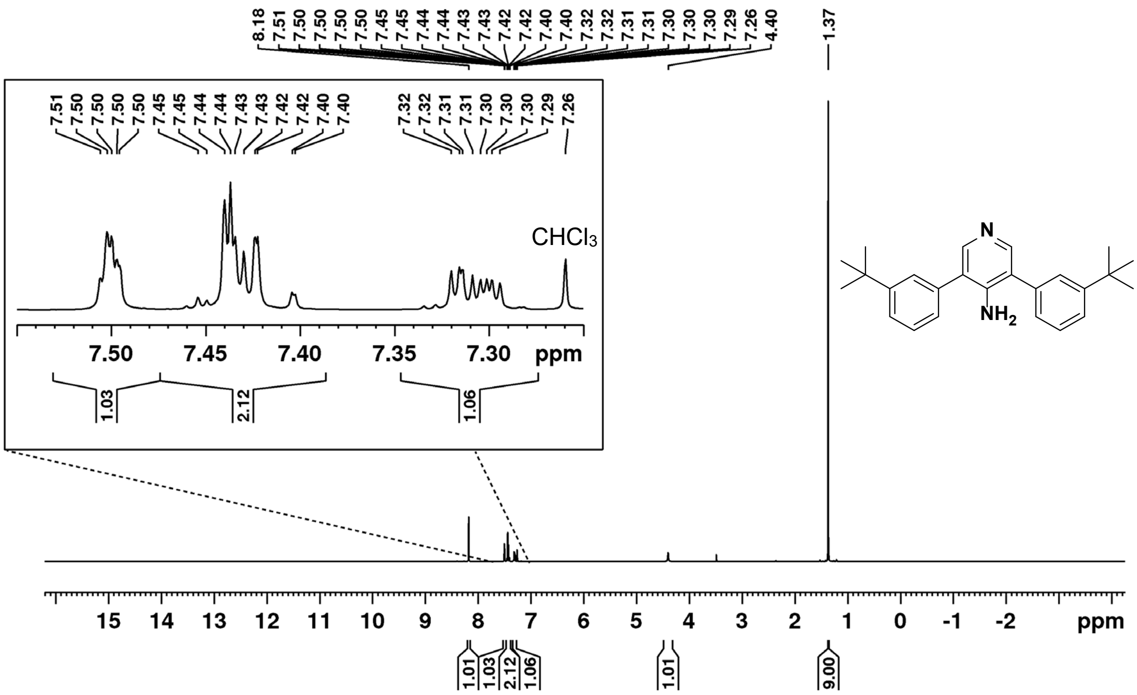

Figure 24. ^{1}H NMR spectrum of **7b** (400 MHz, $CDCl_3$, rt.).

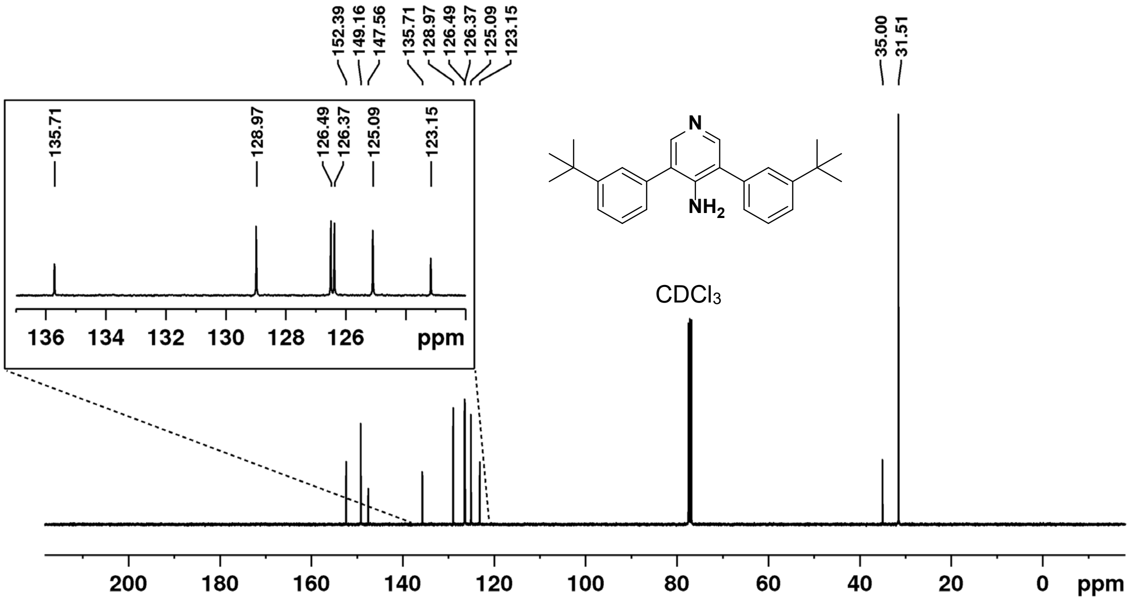

Figure 25. ^{13}C NMR spectrum of **7b** (100 MHz, $CDCl_3$, rt.).

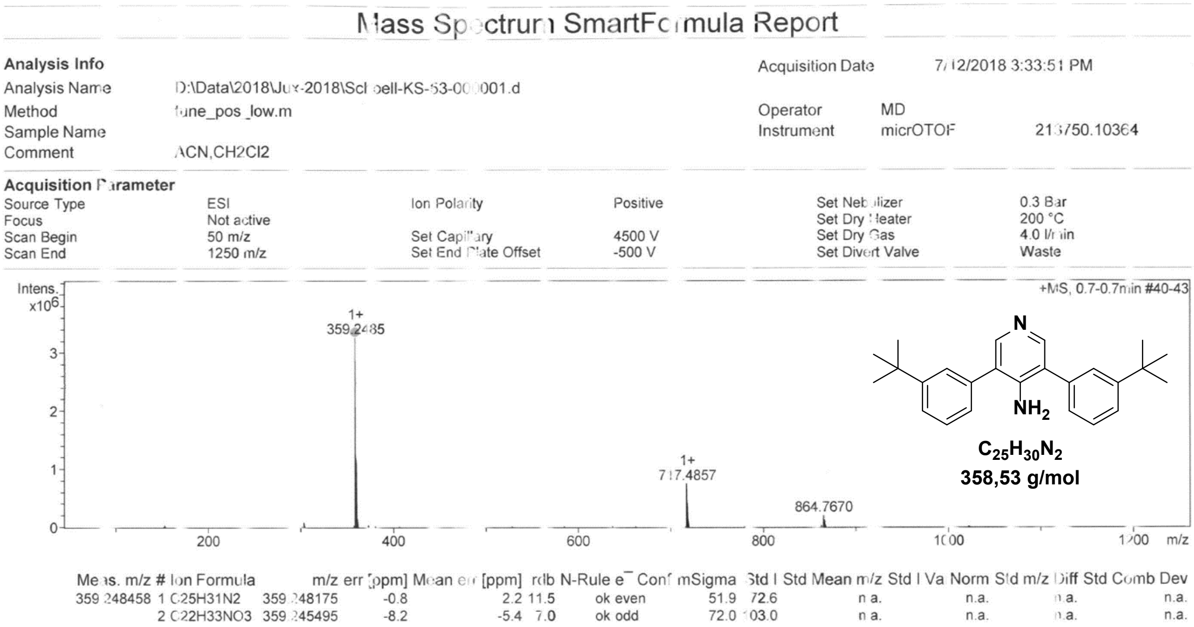

Figure 26. HRMS (ESI, CH_2Cl_2/Acetonitrile) of **7b**.

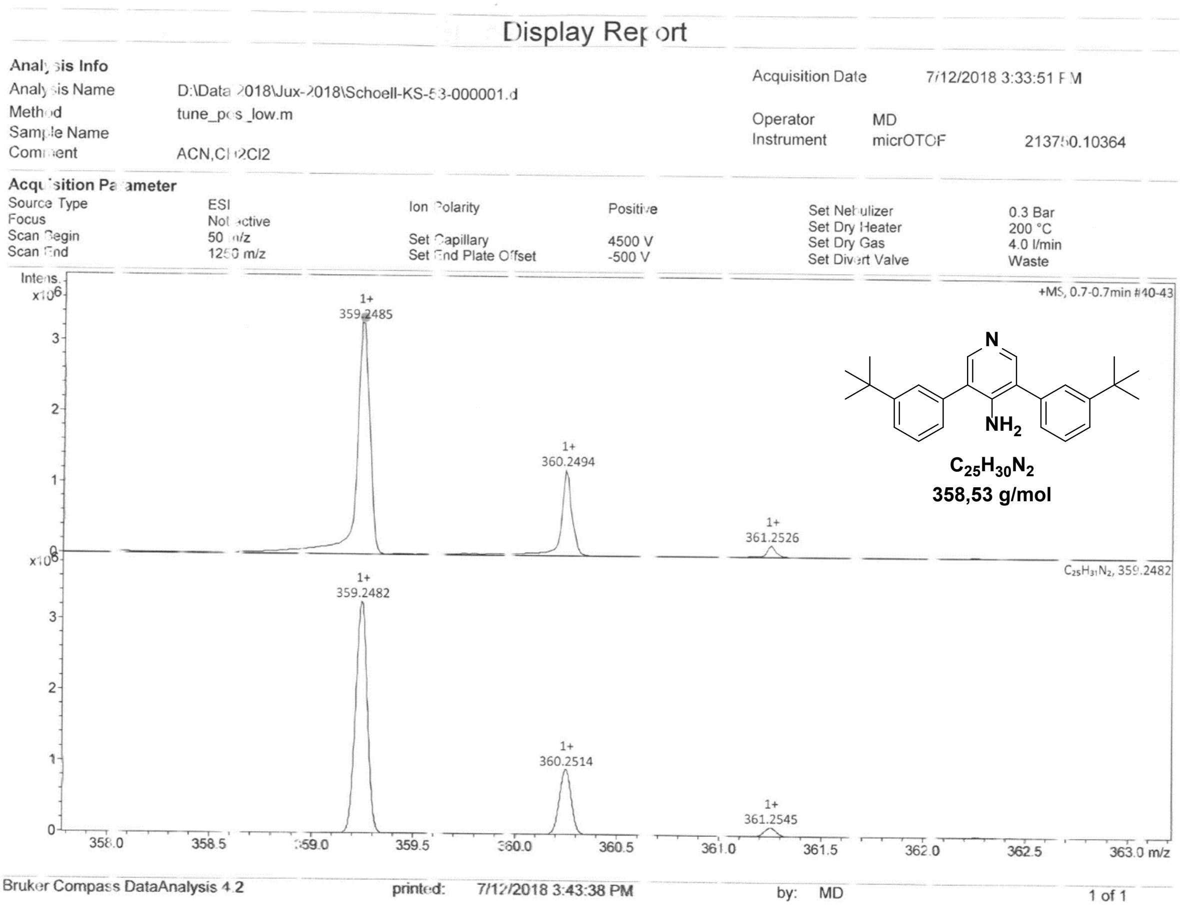

Figure 27. HRMS (ESI, CH_2Cl_2/Acetonitrile) of **7b** (enlargement of the corresponding signal of **7b**, top: measured, bottom: calculated).

3,5-di-(3-*tert*-butyl-phenyl)-4-bromopyridine 8b

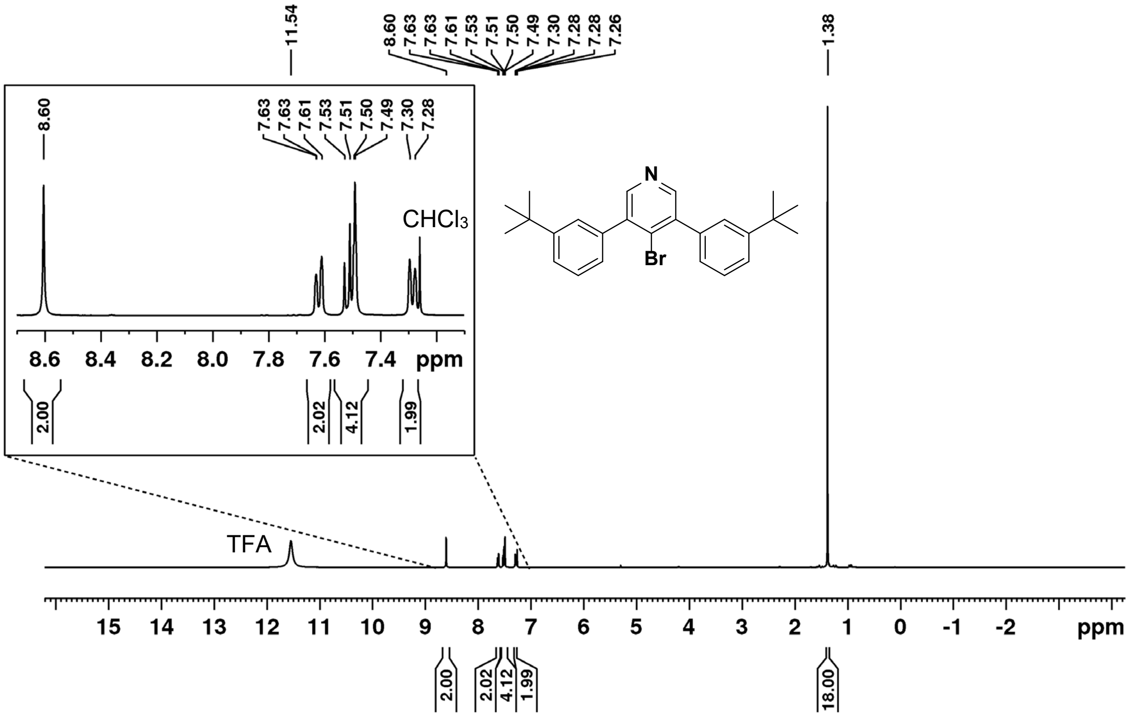

Figure 28. ^{1}H NMR spectrum of **8b** (400 MHz, $CDCl_3$ + TFA, rt.).

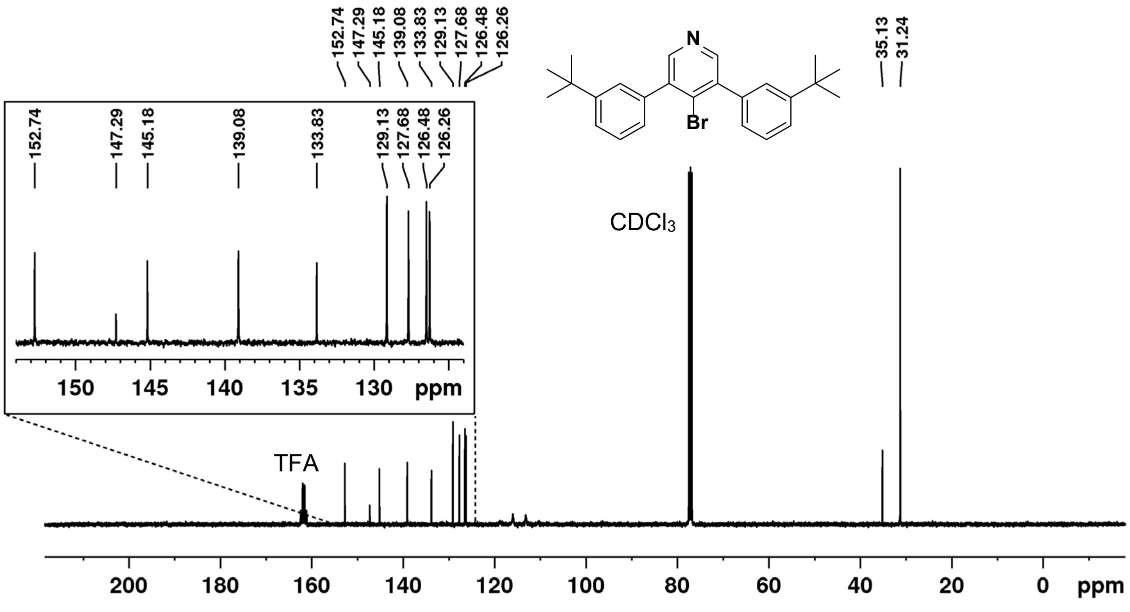

Figure 29. ^{13}C NMR spectrum of **8b** (100 MHz, $CDCl_3$ + TFA, rt.).

Mass Spectrum SmartFormula Report

Analysis Info

Analysis Name	D:\Data\2018\Jux-2018\Schoell-KS-55-appi-.d	Acquisition Date	7/23/2018 10:27:18 AM	
Method	tune_low-APPI.m	Operator	MD	
Sample Name		Instrument	maXis	288882.20183
Comment	TOL			

Acquisition Parameter

Source Type	APPI	Ion Polarity	Positive	Set Nebulizer	2.5 Bar
Focus	Not active	Set Capillary	850 V	Set Dry Heater	220 °C
Scan Begin	50 m/z	Set End Plate Offset	-500 V	Set Dry Gas	1.5 l/min
Scan End	1550 m/z	Set Charging Voltage	0 V	Set Divert Valve	Waste
		Set Corona	0 nA	Set APCI Heater	420 °C

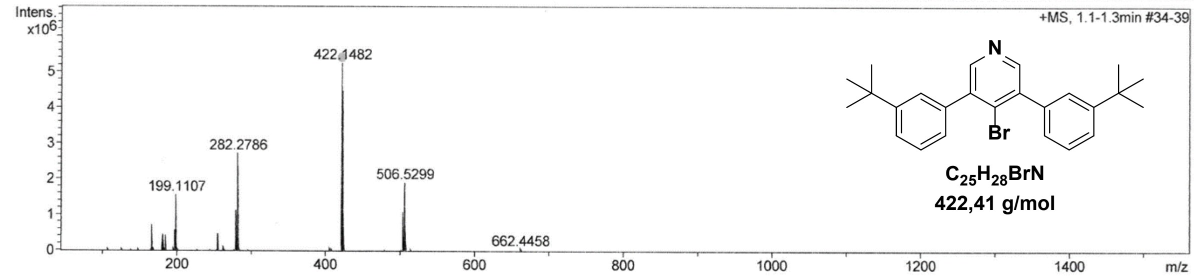

Meas. m/z	#	Ion Formula	m/z	err [ppm]	mSigma	# mSigma	Score	rdb	e⁻ Conf	N-Rule
422.1482	1	C25H29BrN	422.1478	-1.0	77.9	1	100.00	11.5	even	ok

Figure 30. HRMS (APPI, toluene) of **8b**.

Display Report

Analysis Info

Analysis Name	D:\Data\2018\Jux-2018\Schoell-KS-55-appi-.d	Acquisition Date	7/23/2018 10:27:18 AM	
Method	tune_low-APPI.m	Operator	MD	
Sample Name		Instrument	maXis	288882.20183
Comment	TOL			

Acquisition Parameter

Source Type	APPI	Ion Polarity	Positive	Set Nebulizer	2.5 Bar
Focus	Not active	Set Capillary	850 V	Set Dry Heater	220 °C
Scan Begin	50 m/z	Set End Plate Offset	-500 V	Set Dry Gas	1.5 l/min
Scan End	1550 m/z	Set Charging Voltage	0 V	Set Divert Valve	Waste
		Set Corona	0 nA	Set APCI Heater	420 °C

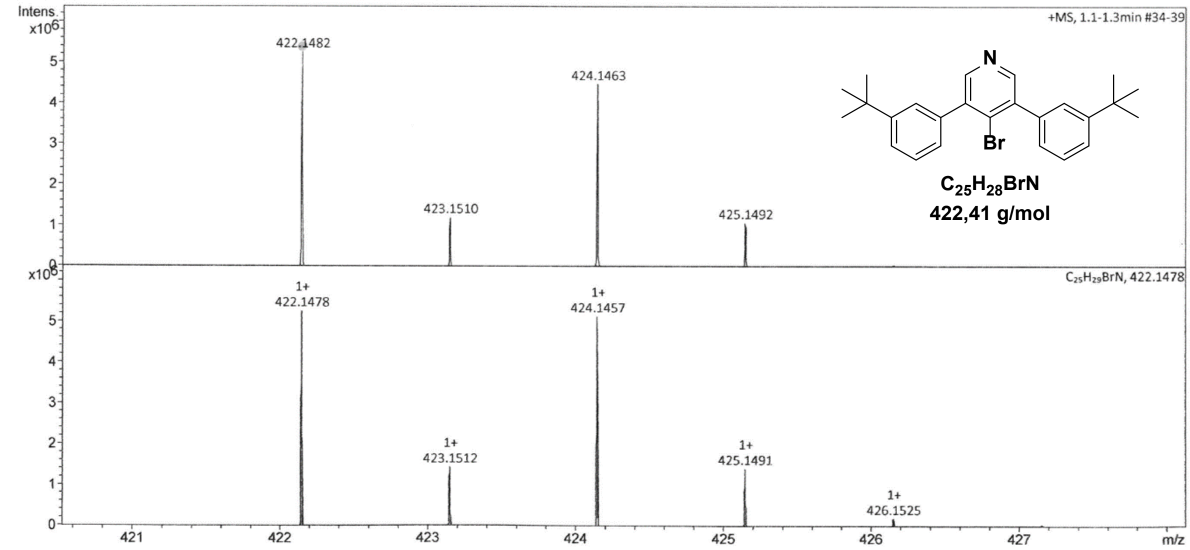

Figure 31. HRMS (APPI, toluene) of **8b** (enlargement of the corresponding signal of **8b**, top: measured, bottom: calculated).

5.3 Spectra for the synthesis of pseudo-HAB precursors 9a/b

Tris-*tert*-butyl pseudo-pyridine HAB 9a

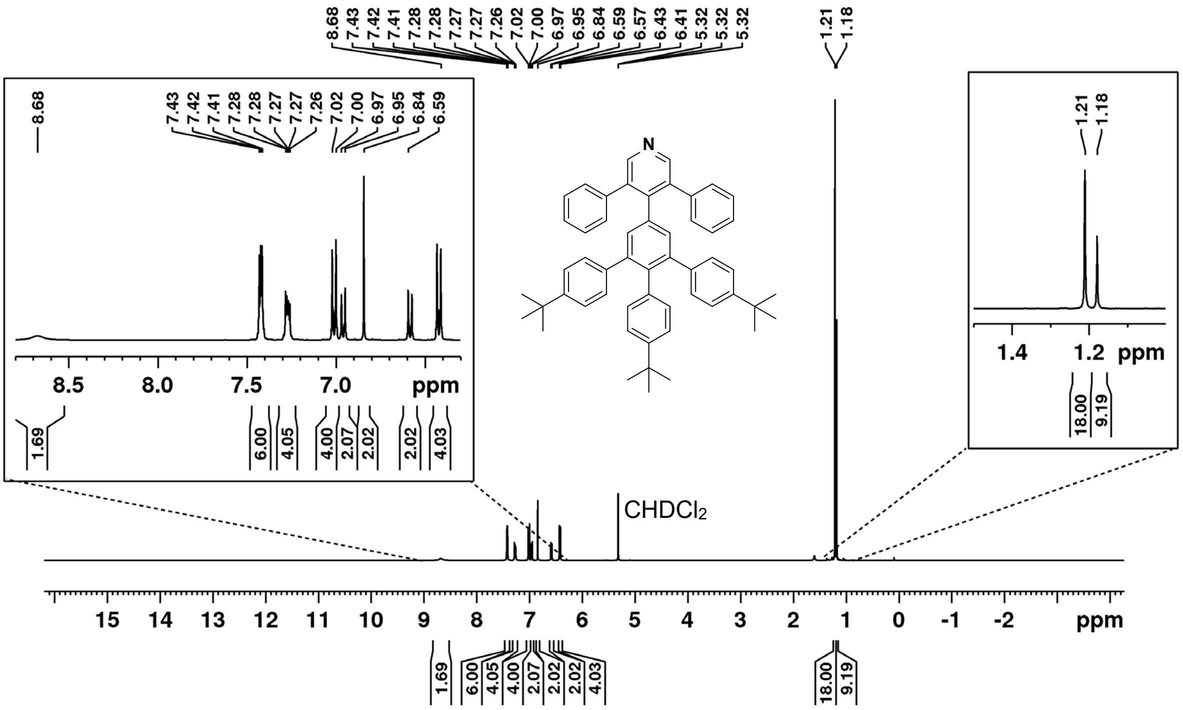

Figure 32. ^{1}H NMR spectrum of **9a** (400 MHz, CD_2Cl_2, rt.).

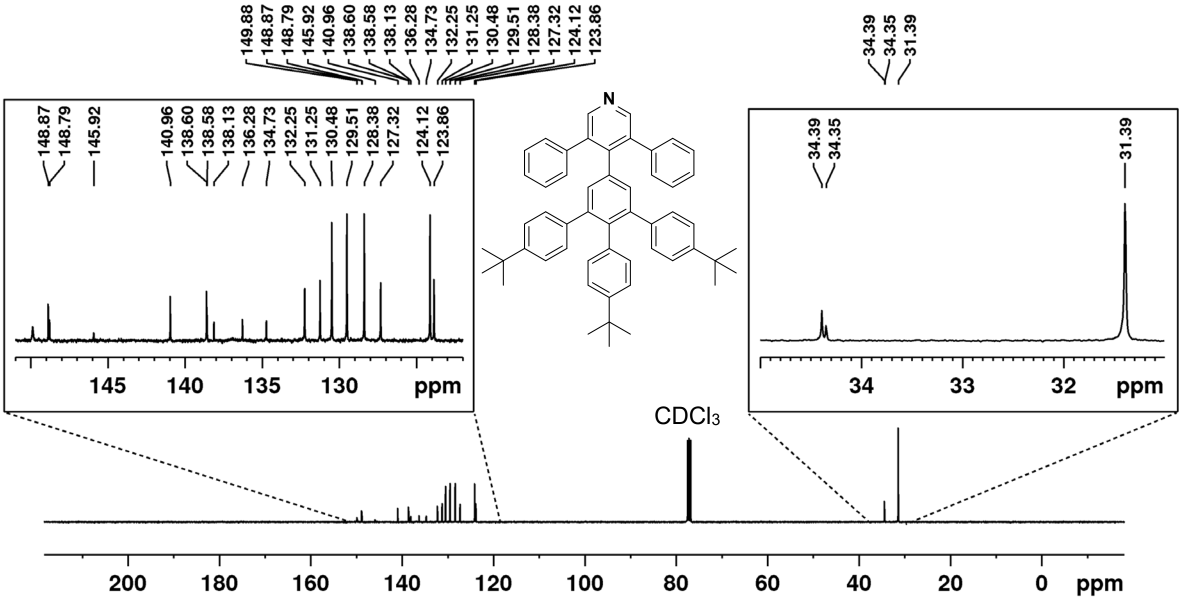

Figure 33. ^{13}C NMR spectrum of **9a** (100 MHz, $CDCl_3$, rt.).

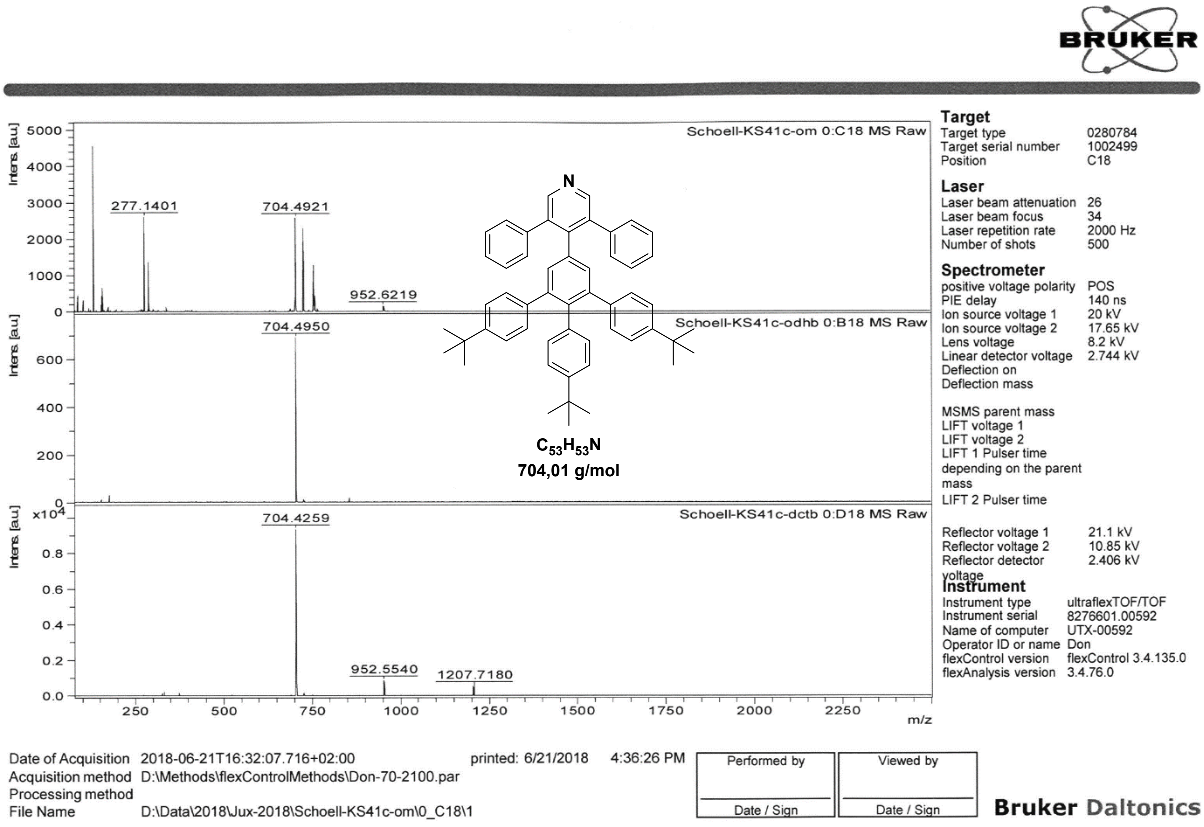

Figure 34. MS (MALDI, top: without matrix, middle: DHB, bottom: DCTB) of **9a**.

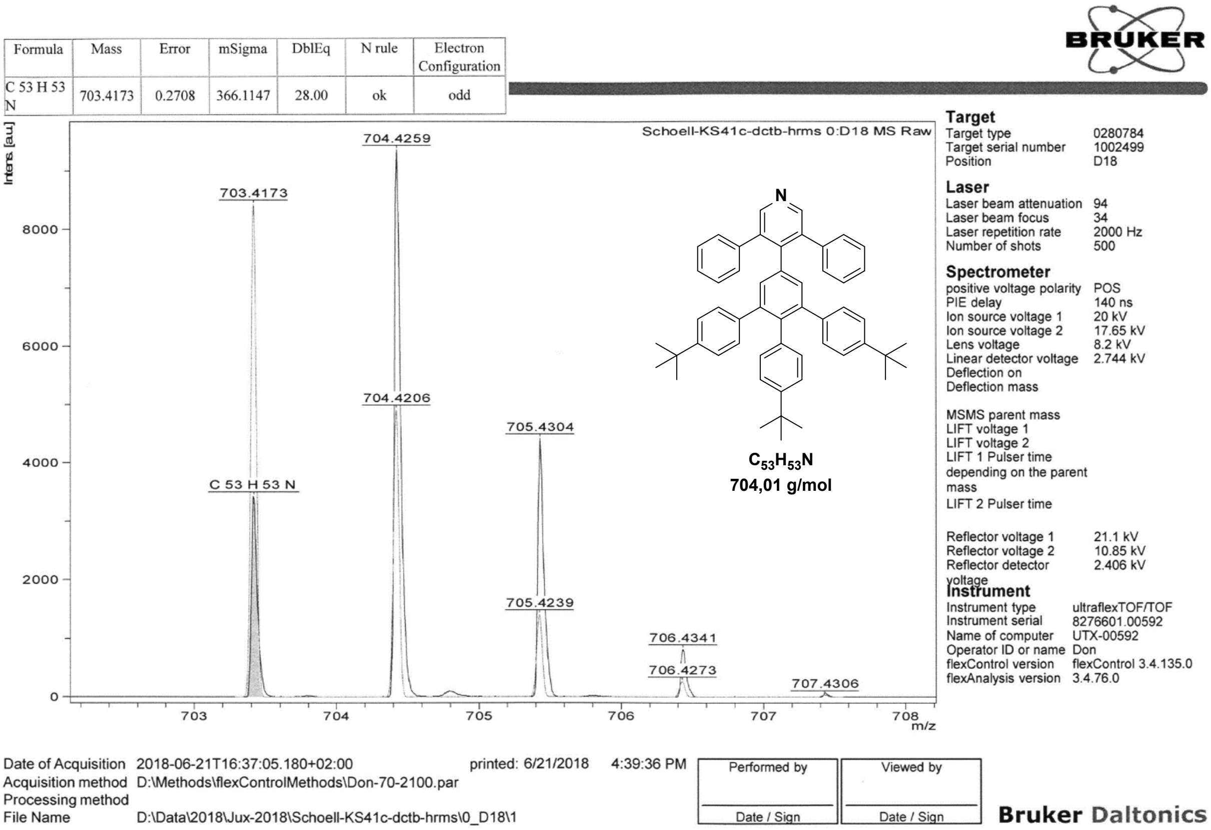

Figure 35. HRMS (MALDI, DCTB) of **9a.** Measured (black) and calculated (grey) spectra overlaid.

Pentakis-*tert*-butyl pseudo-pyridine HAB 9b

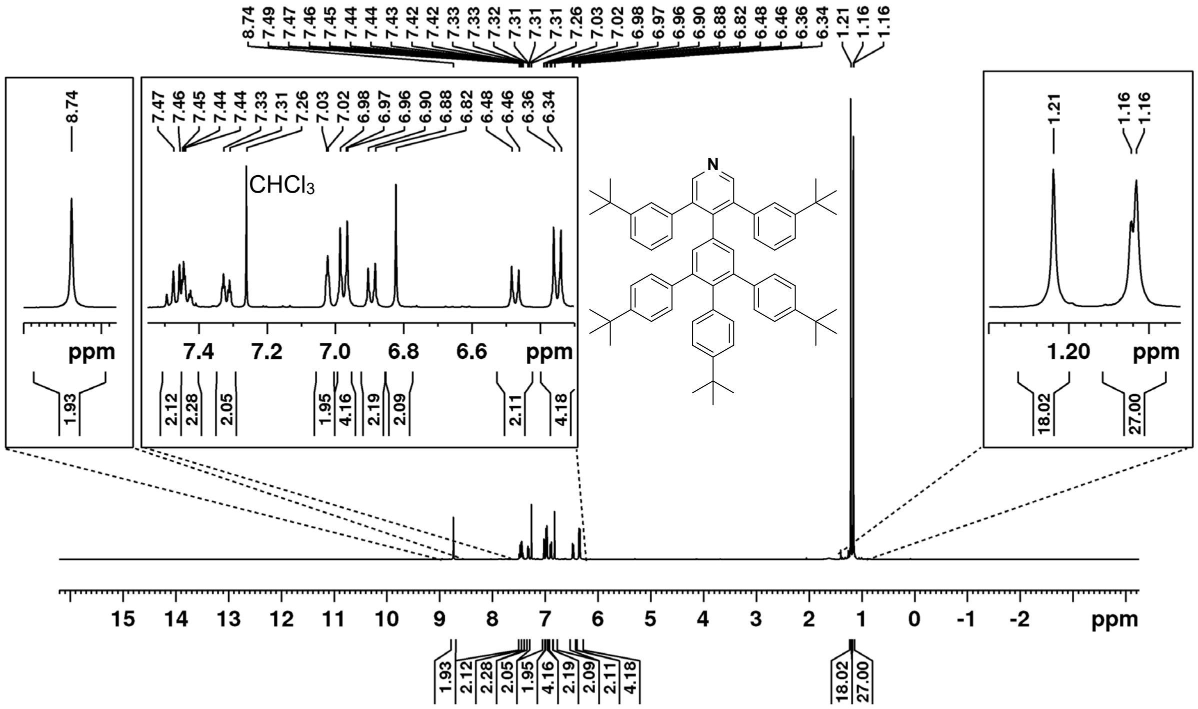

Figure 36. ^{1}H NMR spectrum of **9b** (400 MHz, $CDCl_3$, rt.).

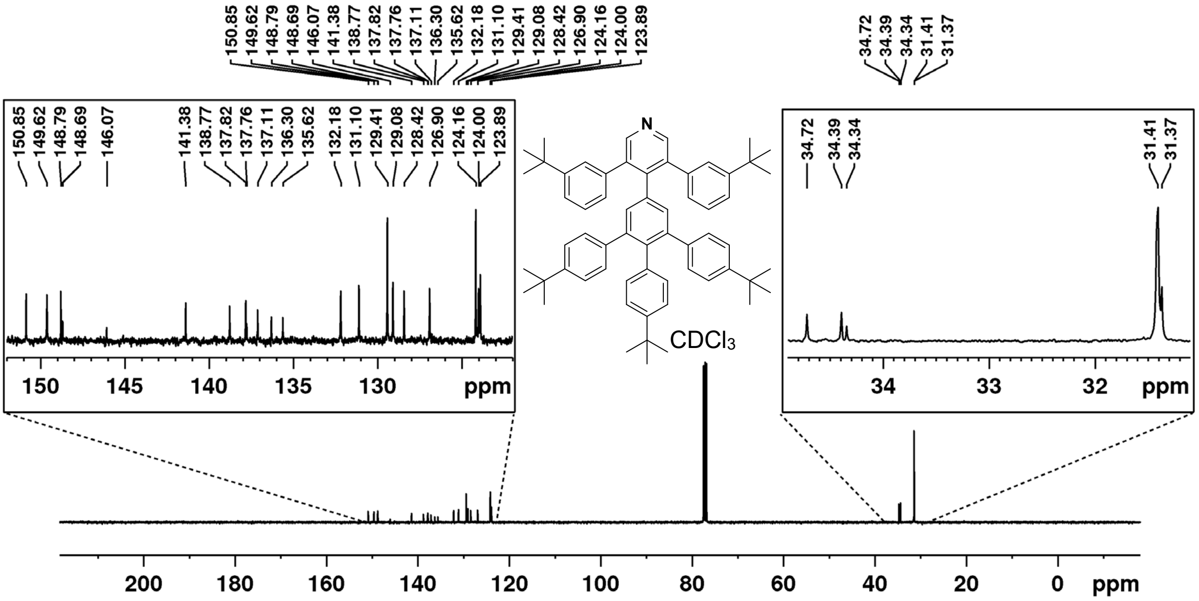

Figure 37. ^{13}C NMR spectrum of **9b** (100 MHz, $CDCl_3$, rt.).

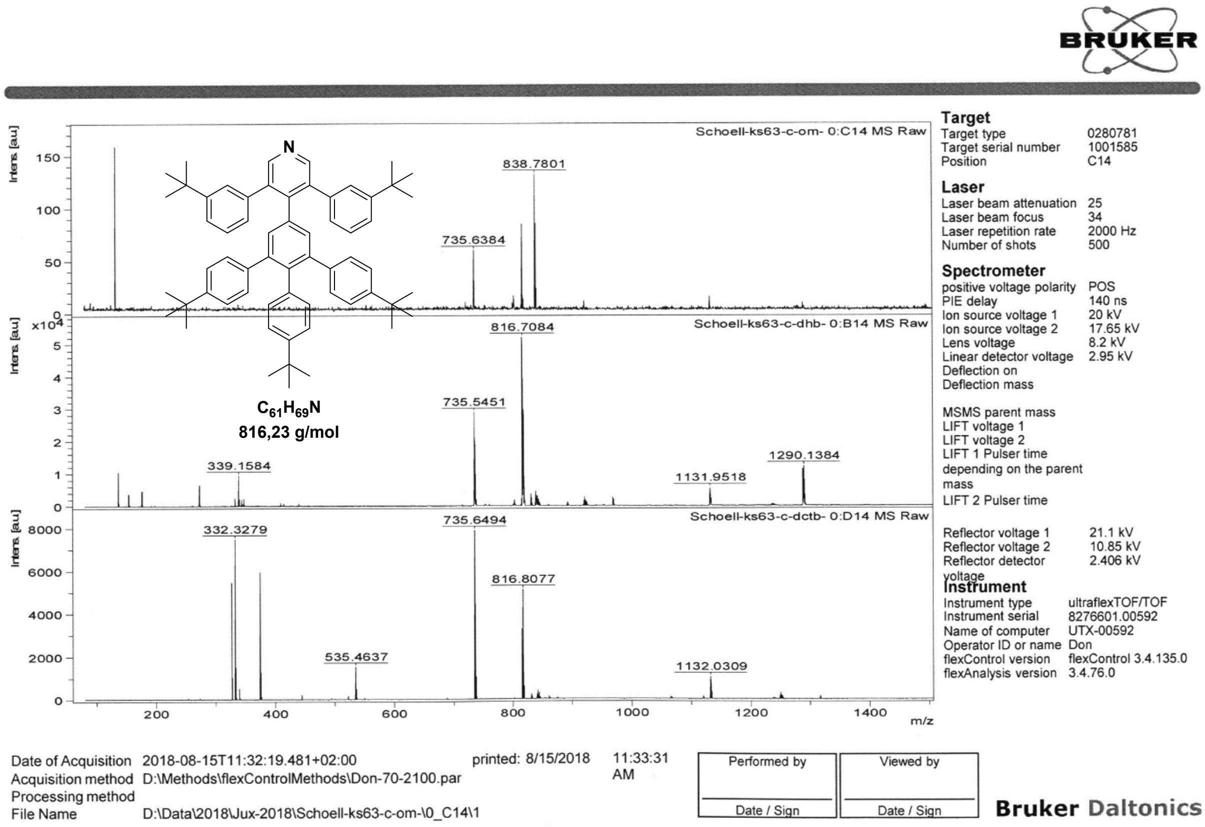

Figure 38. MS (MALDI, top: without matrix, middle: DHB, bottom: DCTB) of **9b.**

Formula	Mass	Error	mSigma	DblEq	N rule	Electron Configuration
C 61 H 70 N	816.5503	1.0985	157.3085	27.50	ok	even
C 61 H 69 N	815.5425	4.3758	288.4359	28.00	ok	odd

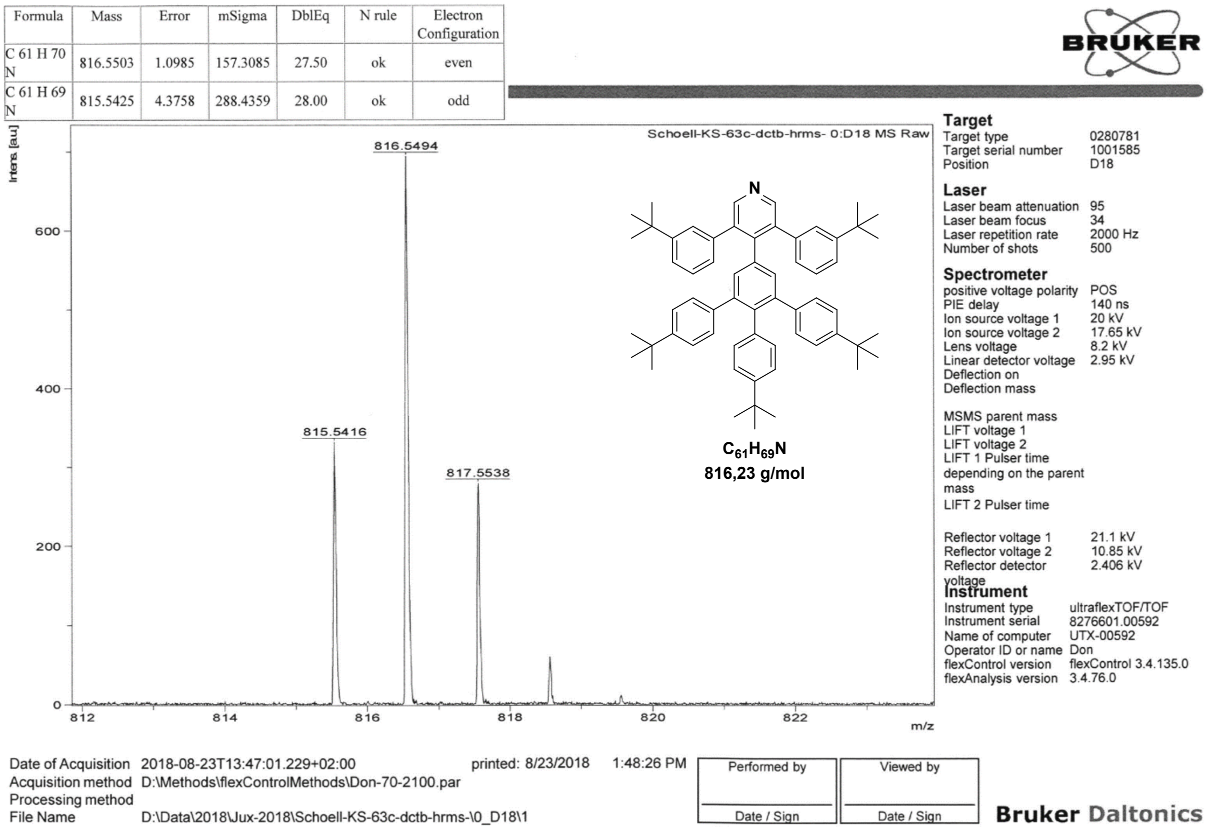

Figure 39. HRMS (MALDI, DCTB) of **9b.**

5.4 Spectra for the synthesis of pyridine-HBCs 10a/b

π-extended pyridine 10a

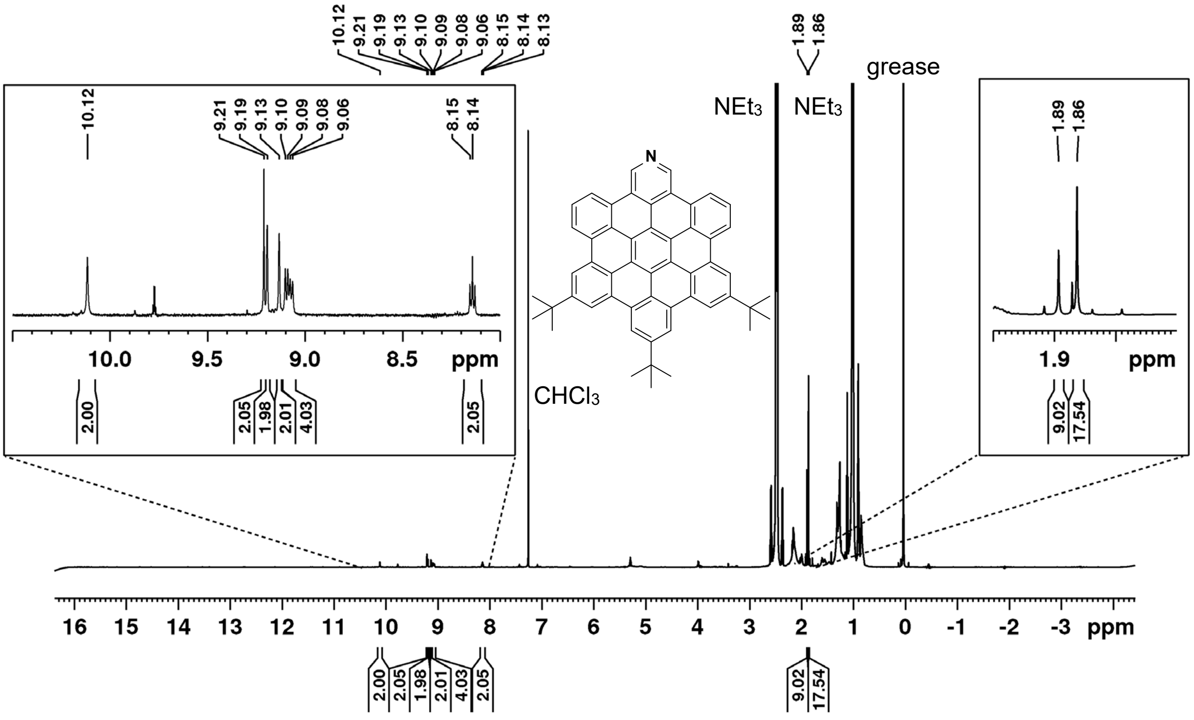

Figure 40. ^{1}H NMR spectrum of **10a** (600 MHz, $CDCl_3/CS_2/NEt_3$, rt.).

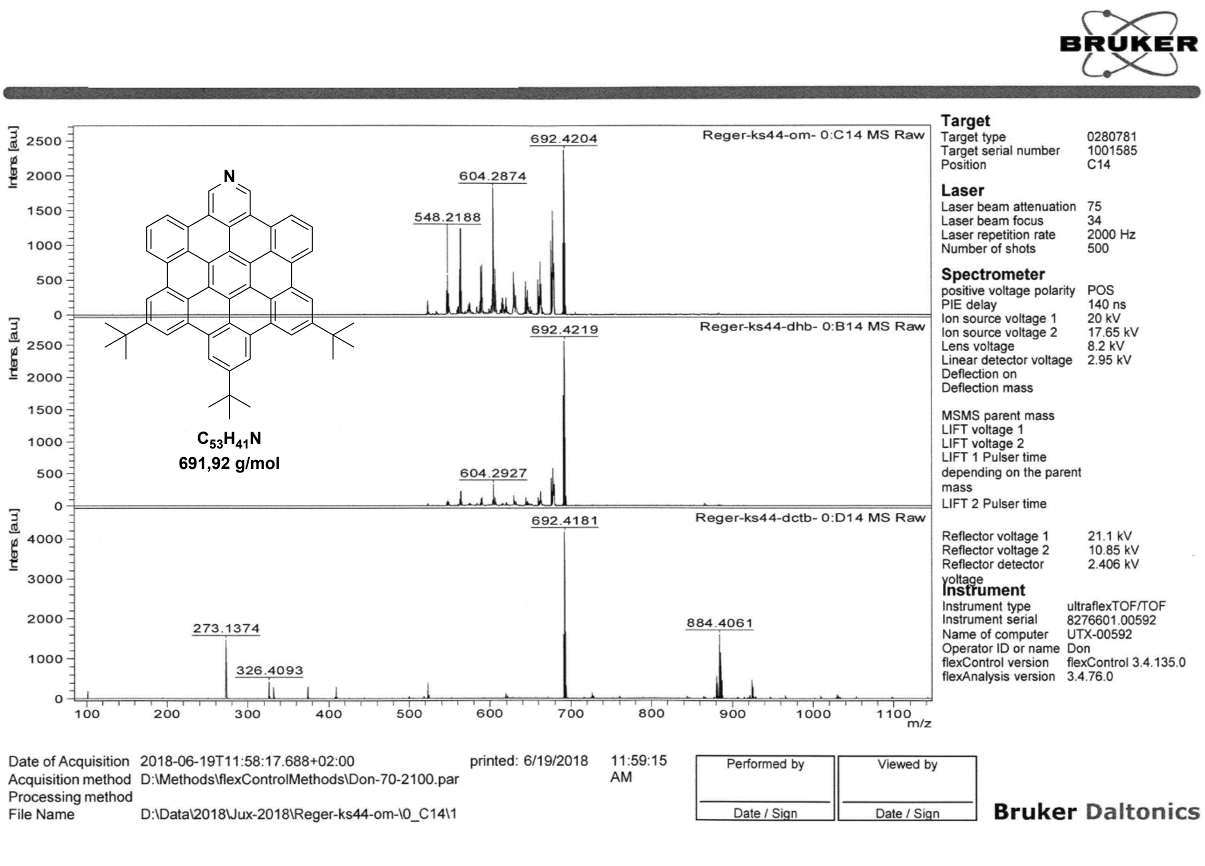

Figure 41. MS (MALDI, top: without matrix, middle: DHB, bottom: DCTB) of **10a.**

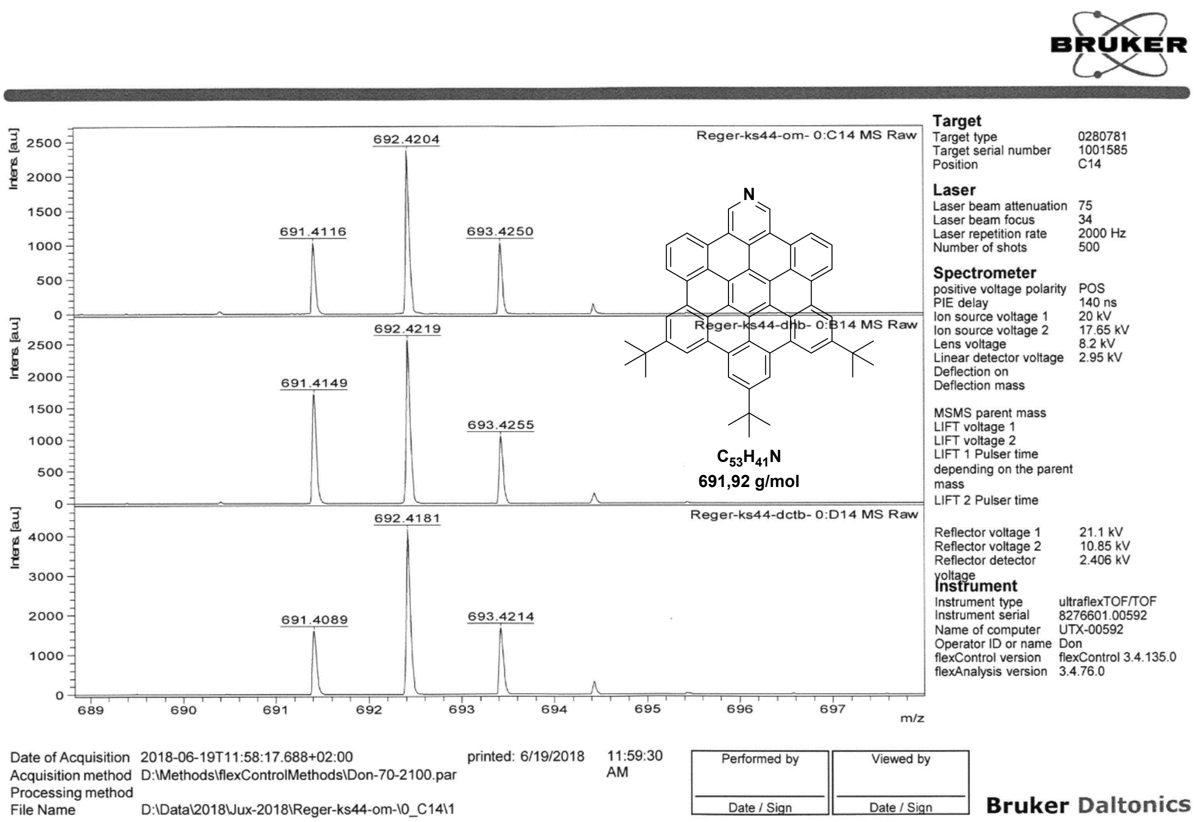

Figure 42. MS (MALDI, top: without matrix, middle: DHB, bottom: DCTB) of **10a** (cutout of the respective peak of **10a**).

π-extended pyridine 10b

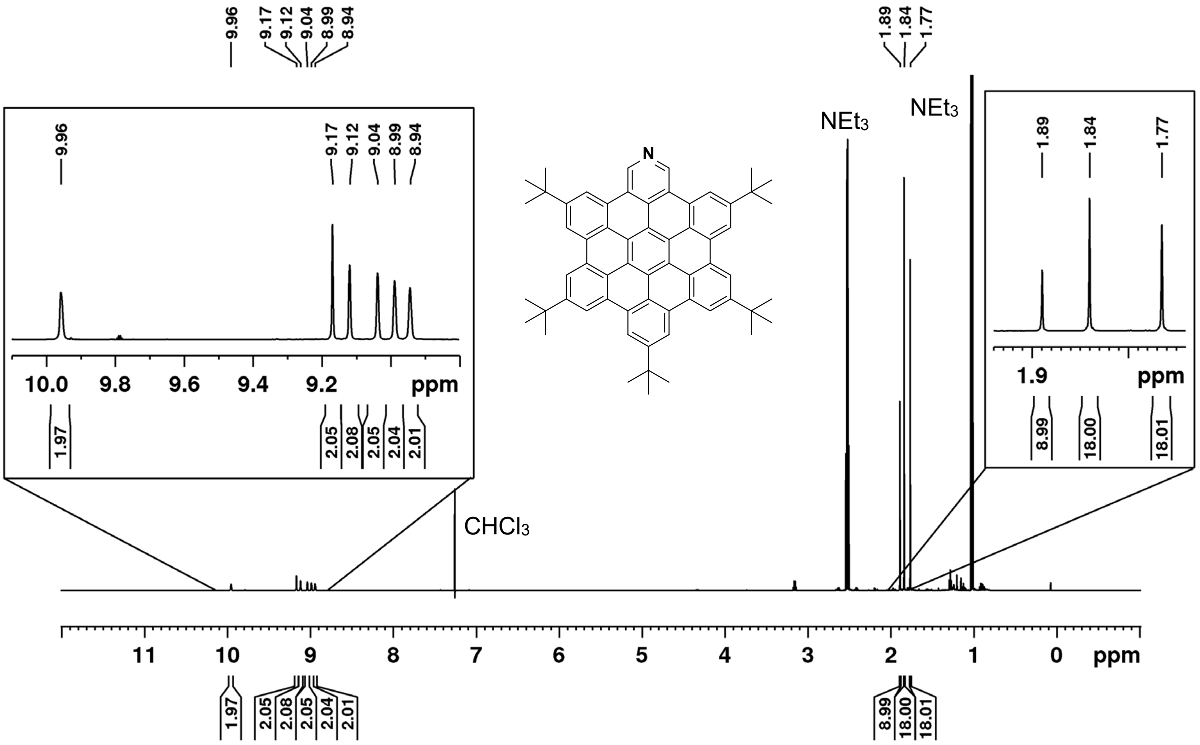

Figure 43. ^{1}H NMR spectrum of **10b** (600 MHz, $CDCl_3$/NEt_3, rt.).

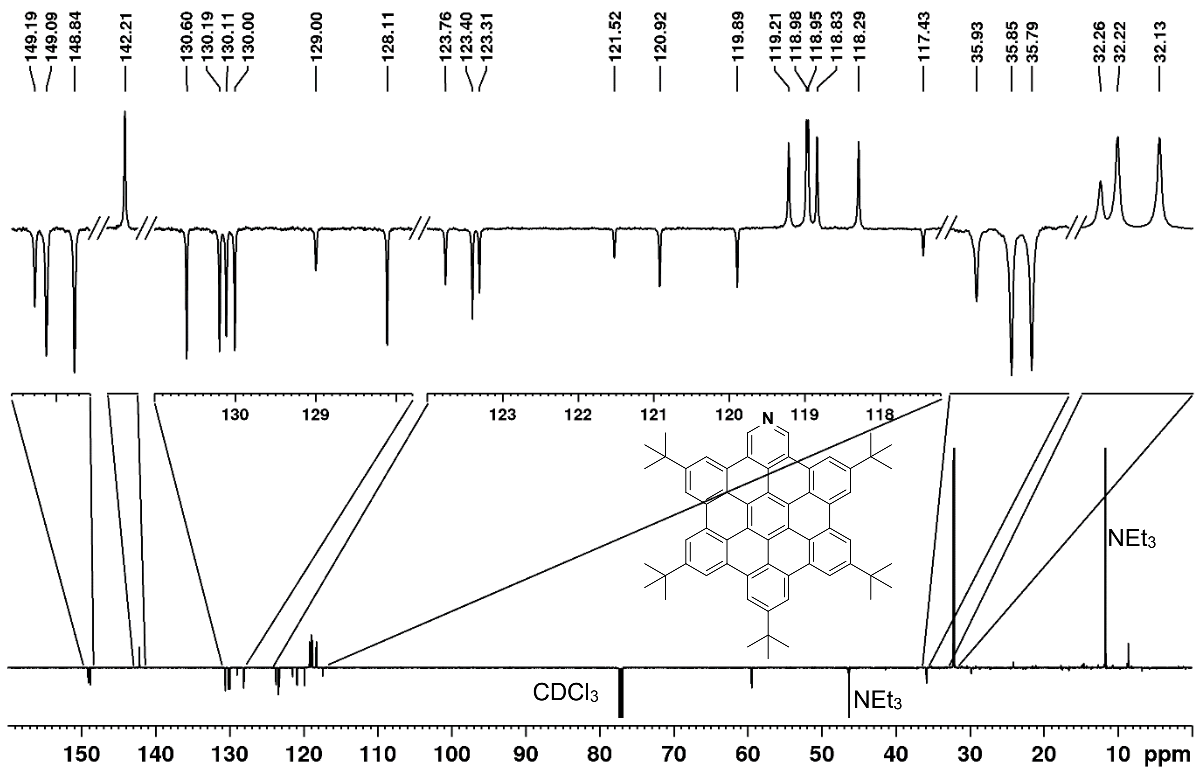

Figure 44. DEPTq-135 spectrum of **10b** (150 MHz, $CDCl_3$/Et_3N, rt.).

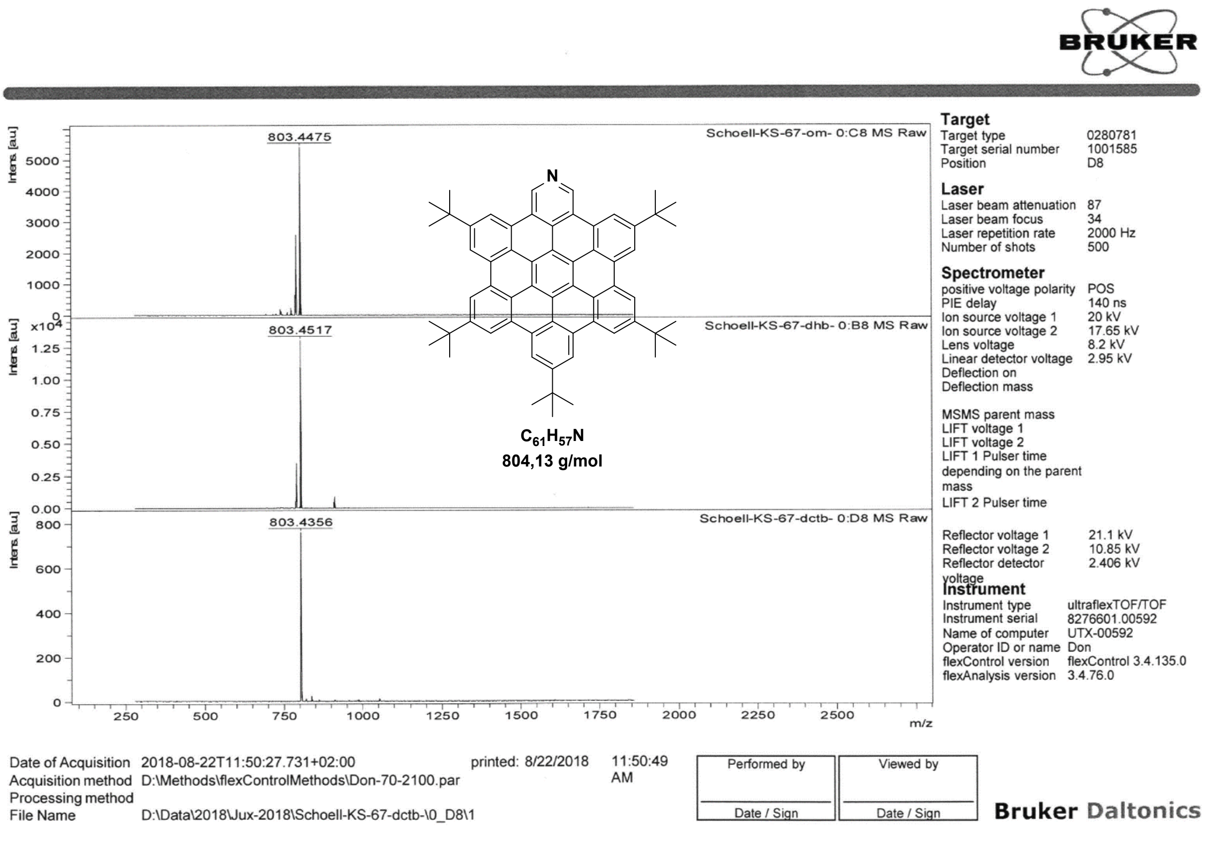

Figure 45. MS (MALDI, top: without matrix, middle: DHB, bottom: DCTB) of **10b.**

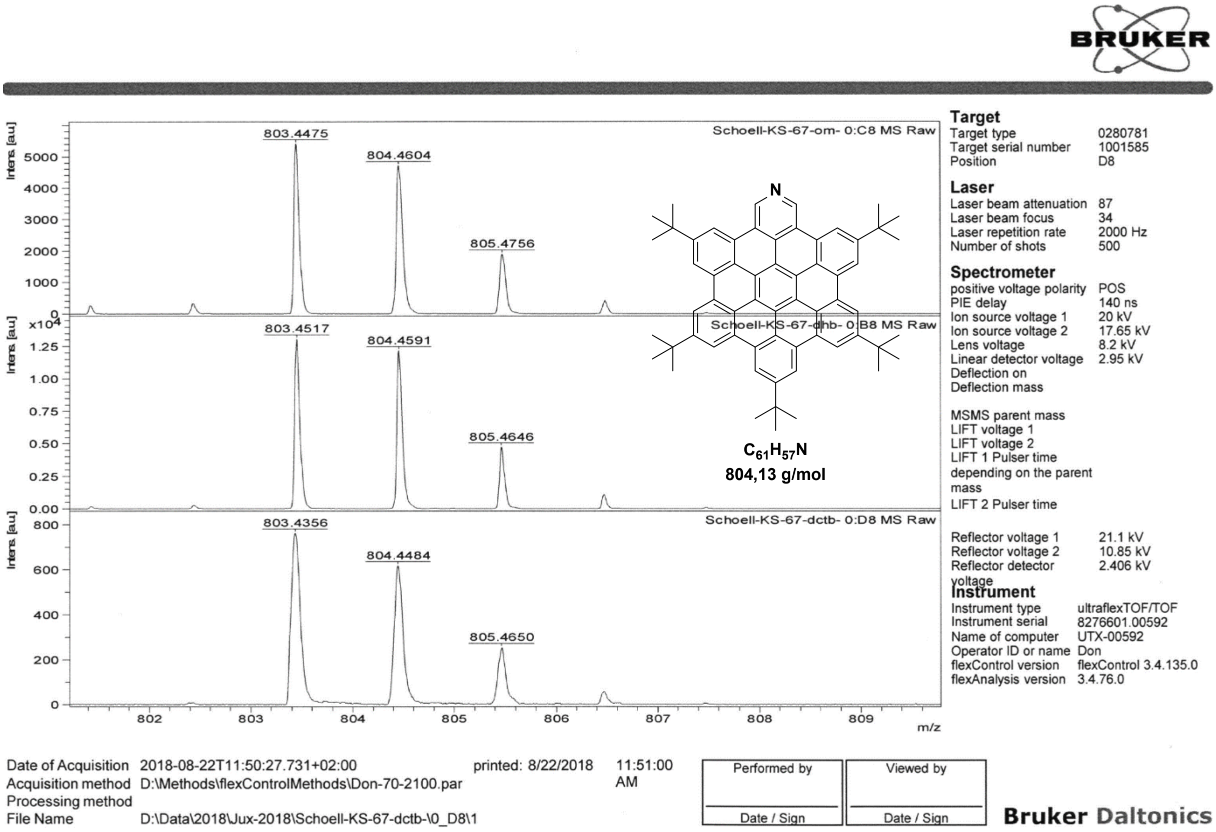

Figure 46. MS (MALDI, top: without matrix, middle: DHB, bottom: DCTB) of **10b** (Cutout of the respective peak of **10b**).

Formula	Mass	Error	mSigma	DblEq	N rule	Electron Configuration
C 61 H 57 N	803.4486	1.2868	109.5154	34.00	ok	odd
C 58 H 59 O 3	803.4459	2.0489	126.0204	29.50	ok	even

Formula	Mass	Error	mSigma	DblEq	N rule	Electron Configuration
C 54 H 59 N O 5	801.4388	2.7070	279.8228	26.00	ok	odd
C 54 H 60 N O 5	802.4466	3.0304	279.8815	25.50	ok	even

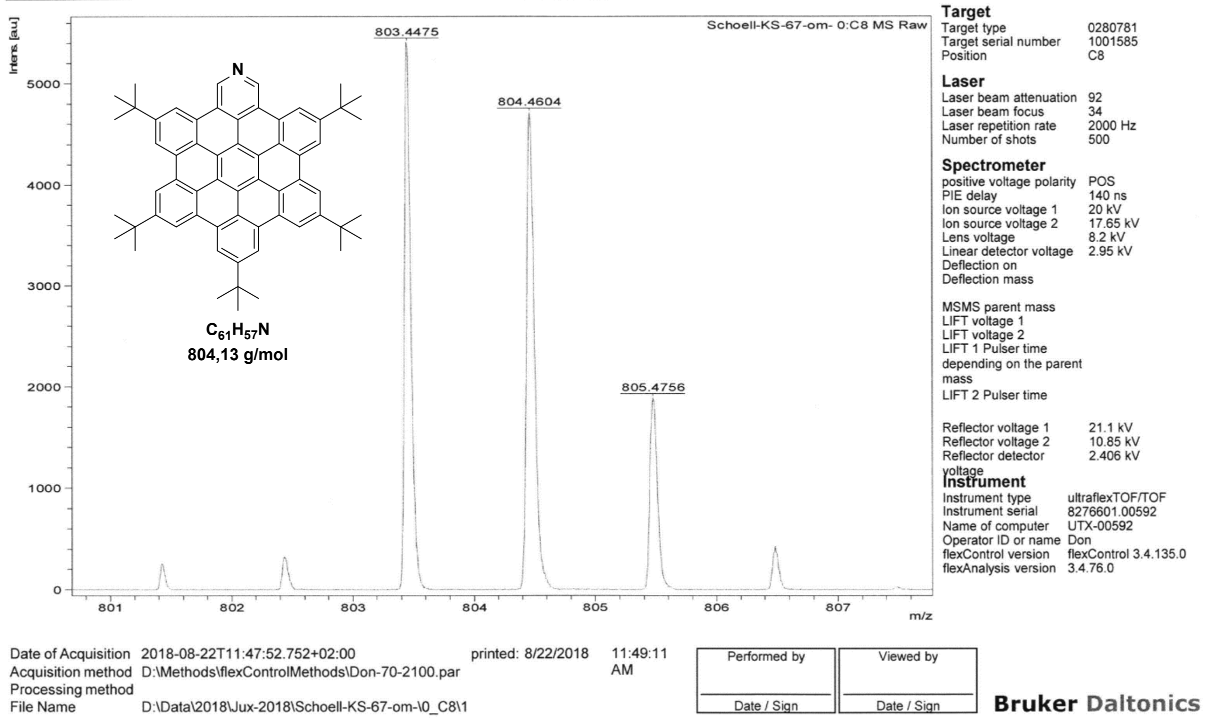

Figure 47. HRMS (LDI) of **10b.**

5.5 Spectra for products from post-functionalization 12/13

π-extended-pyridinium triflate 12

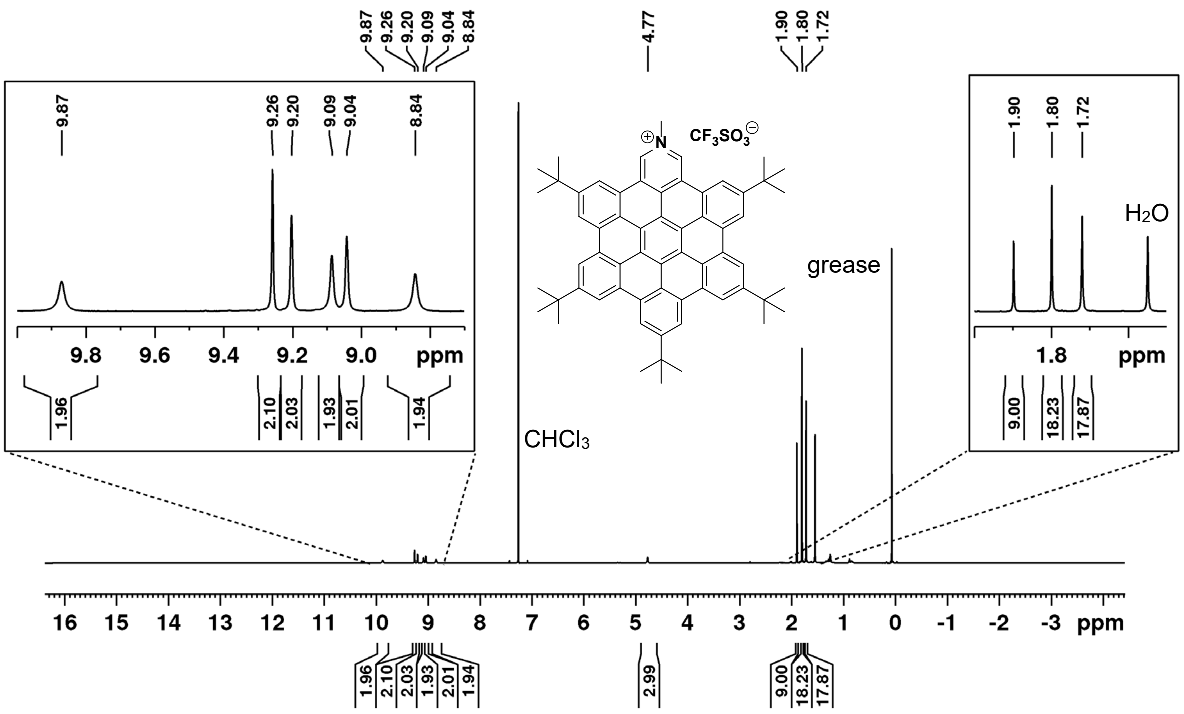

Figure 48. ^{1}H NMR spectrum of **12** (600 MHz, $CDCl_3$, rt.).

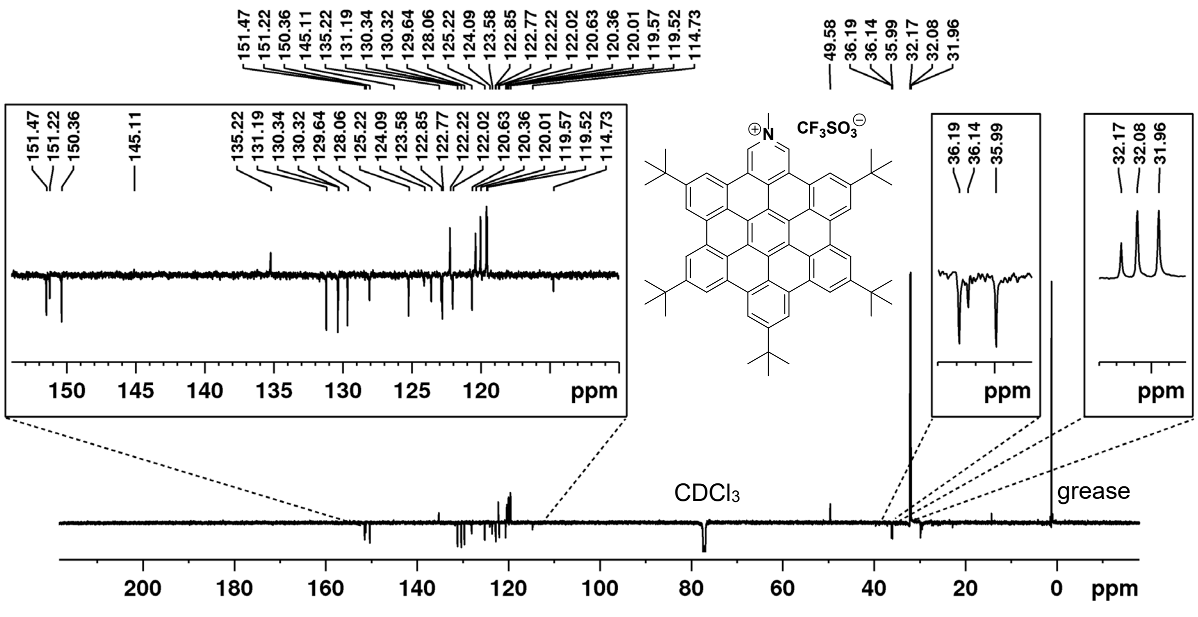

Figure 49. DEPTq-135 spectrum of **12** (150 MHz, $CDCl_3$, rt.).

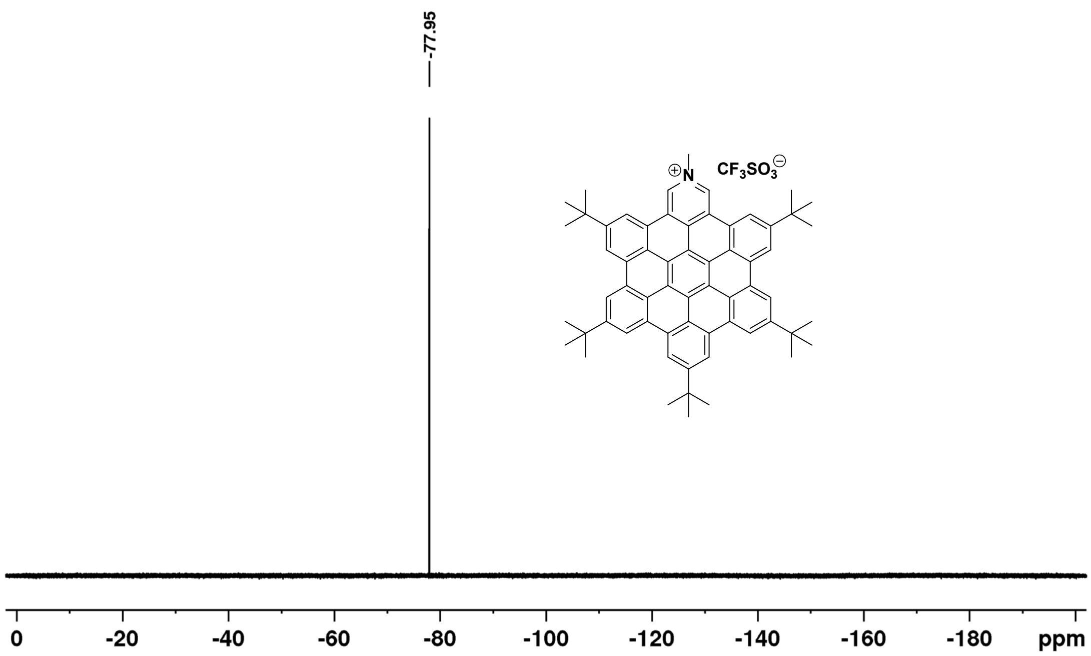

Figure 50. ^{19}F NMR spectrum of **12** (470 MHz, $CDCl_3$, rt.).

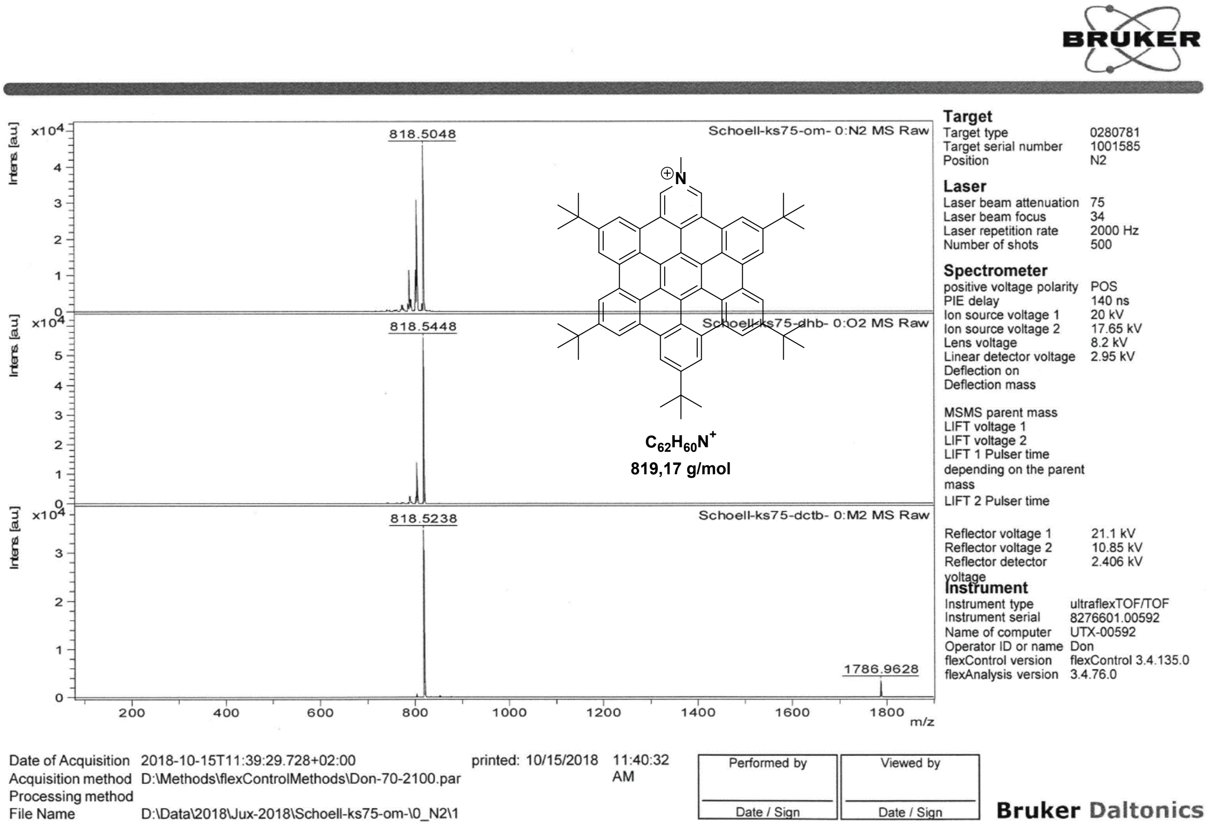

Figure 51. MS (MALDI, top: without matrix, middle: DHB, bottom: DCTB) of **12.**

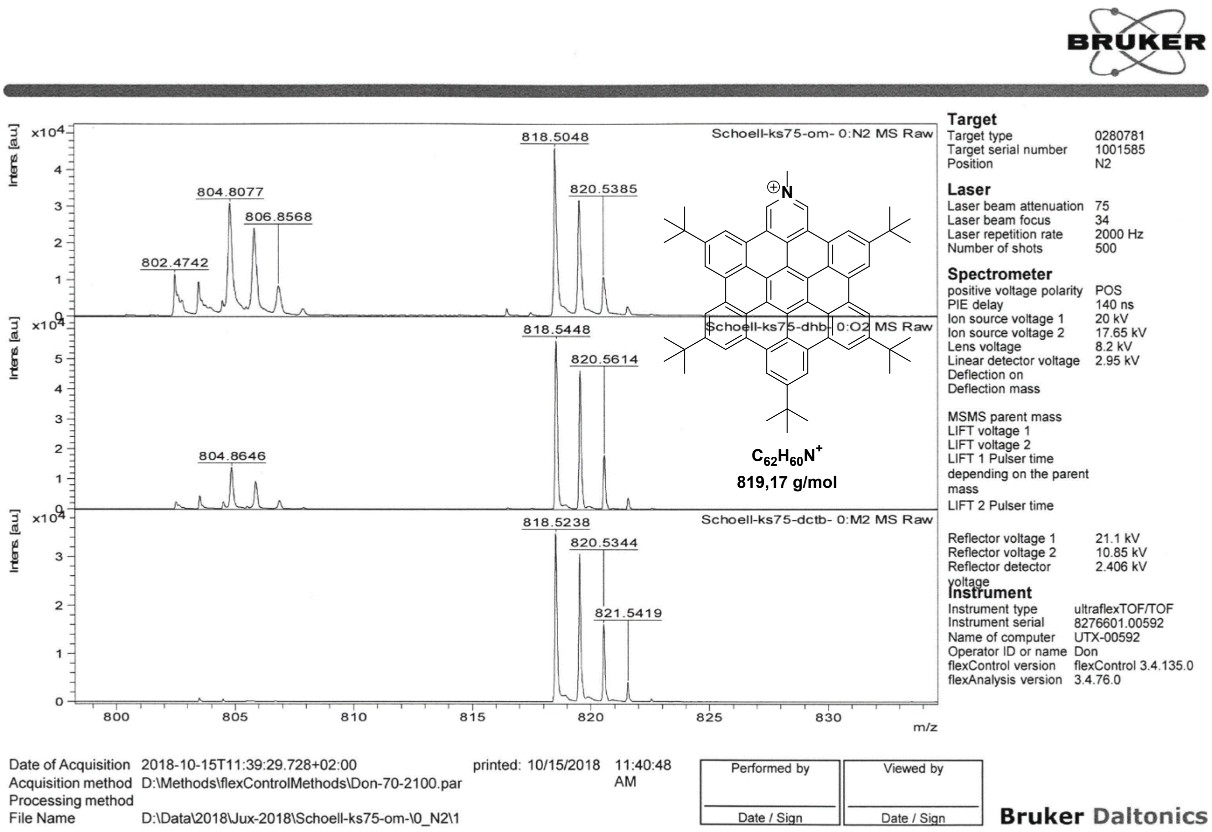

Figure 52. MS (MALDI, top: without matrix, middle: DHB, bottom: DCTB) of **12** (Cutout of the respective peak of **12**).

Formula	Mass	Error	mSigma	DblEq	N rule	Electron Configuration
C 62 H 60 N	818.4720	0.3773	199.6331	33.50	ok	even
C 62 H 58 O	818.4482	29.4674	200.4884	34.00	ok	odd

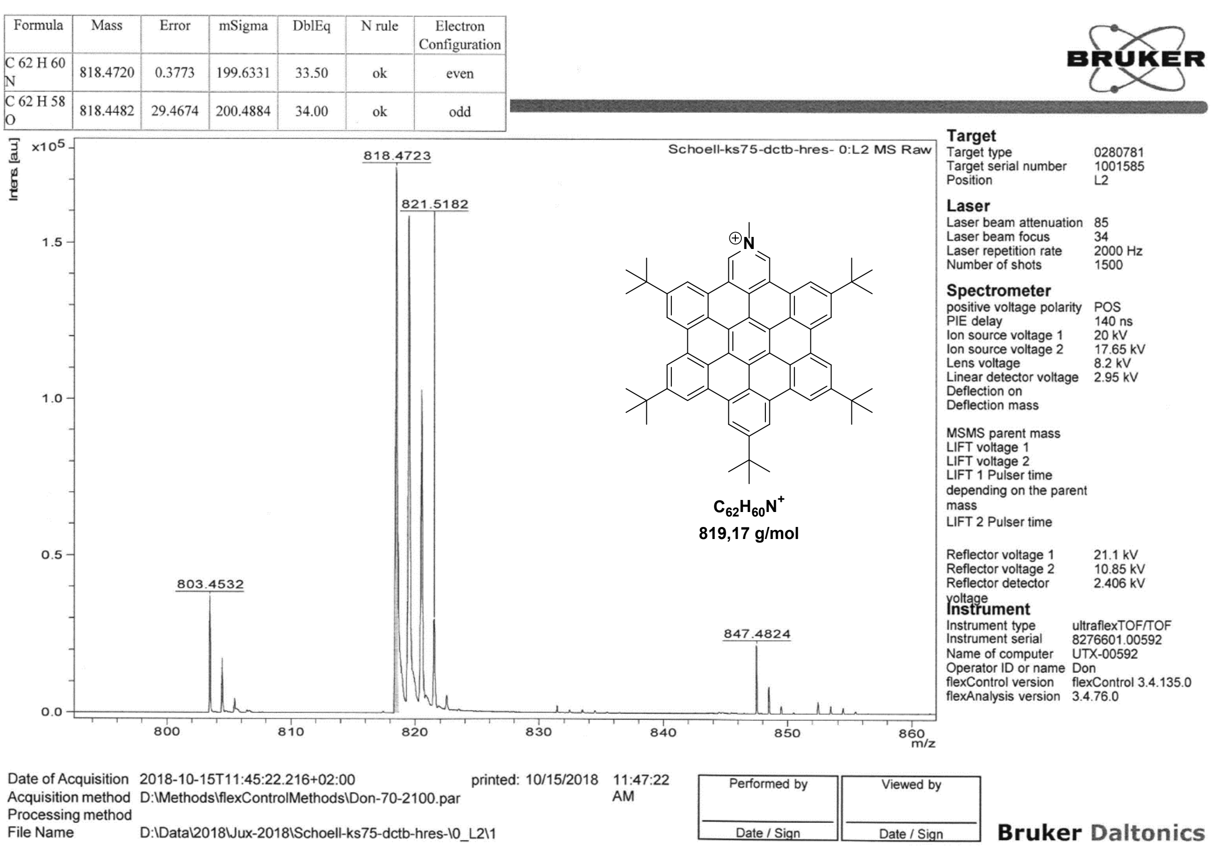

Figure 53. HRMS (MALDI, DCTB) of **12.**

π-extended pyridine N-oxide 13

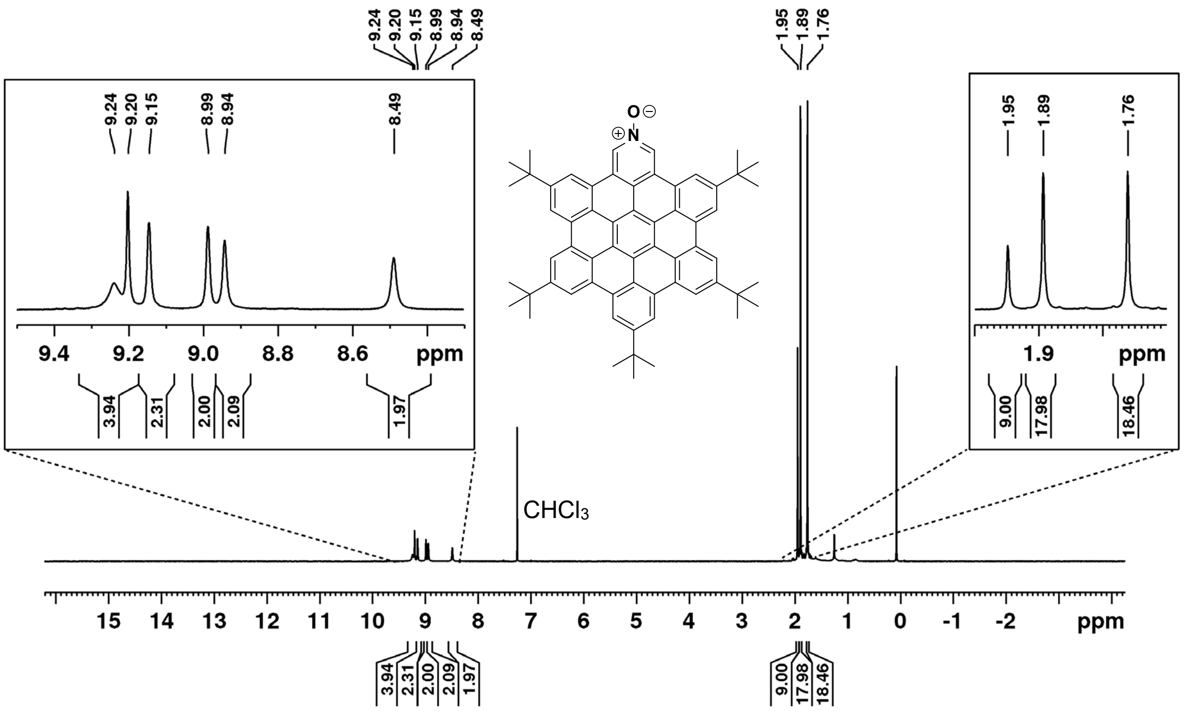

Figure 54. ^{1}H NMR spectrum of **13** (400 MHz, $CDCl_3$, rt.).

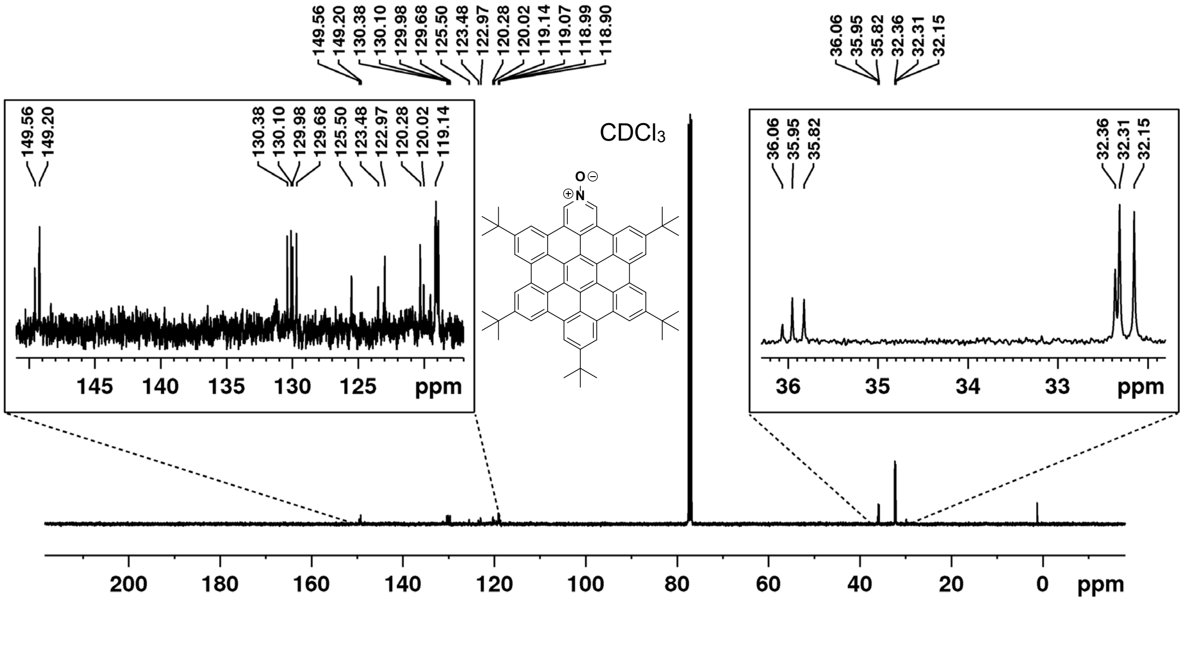

Figure 55. ^{13}C NMR spectrum of **13** (100 MHz, $CDCl_3$, rt.).

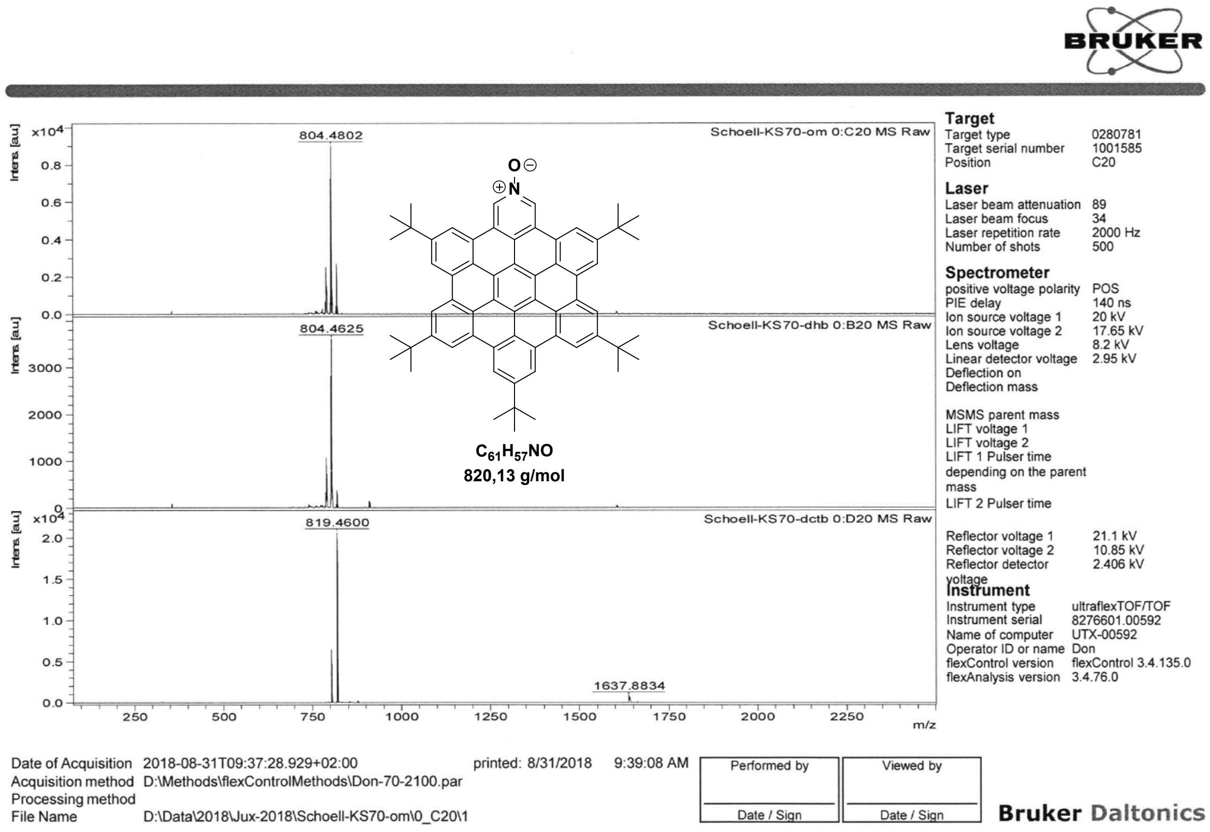

Figure 56. MS (MALDI, top: without matrix, middle: DHB, bottom: DCTB) of **13.**

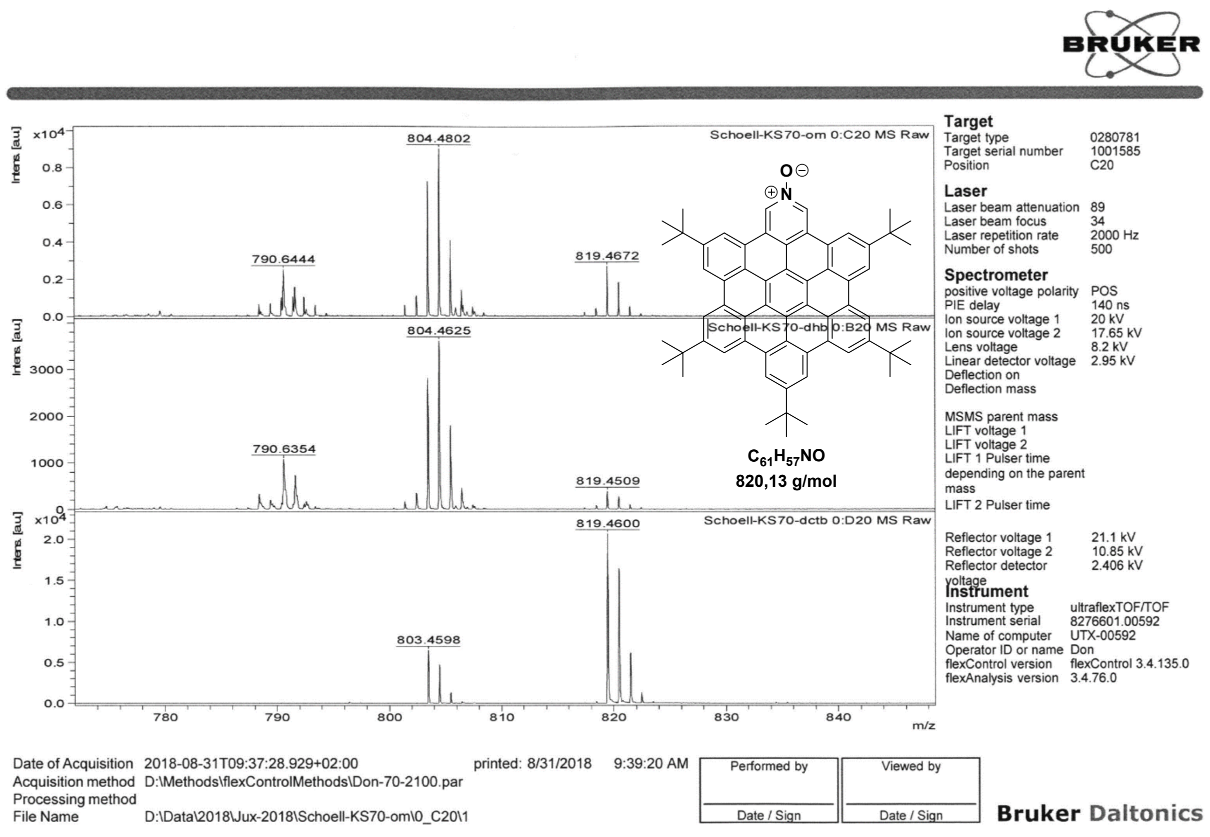

Figure 57. MS (MALDI, top: without matrix, middle: DHB, bottom: DCTB) of **13** (Cutout of the respective peak of **13**).

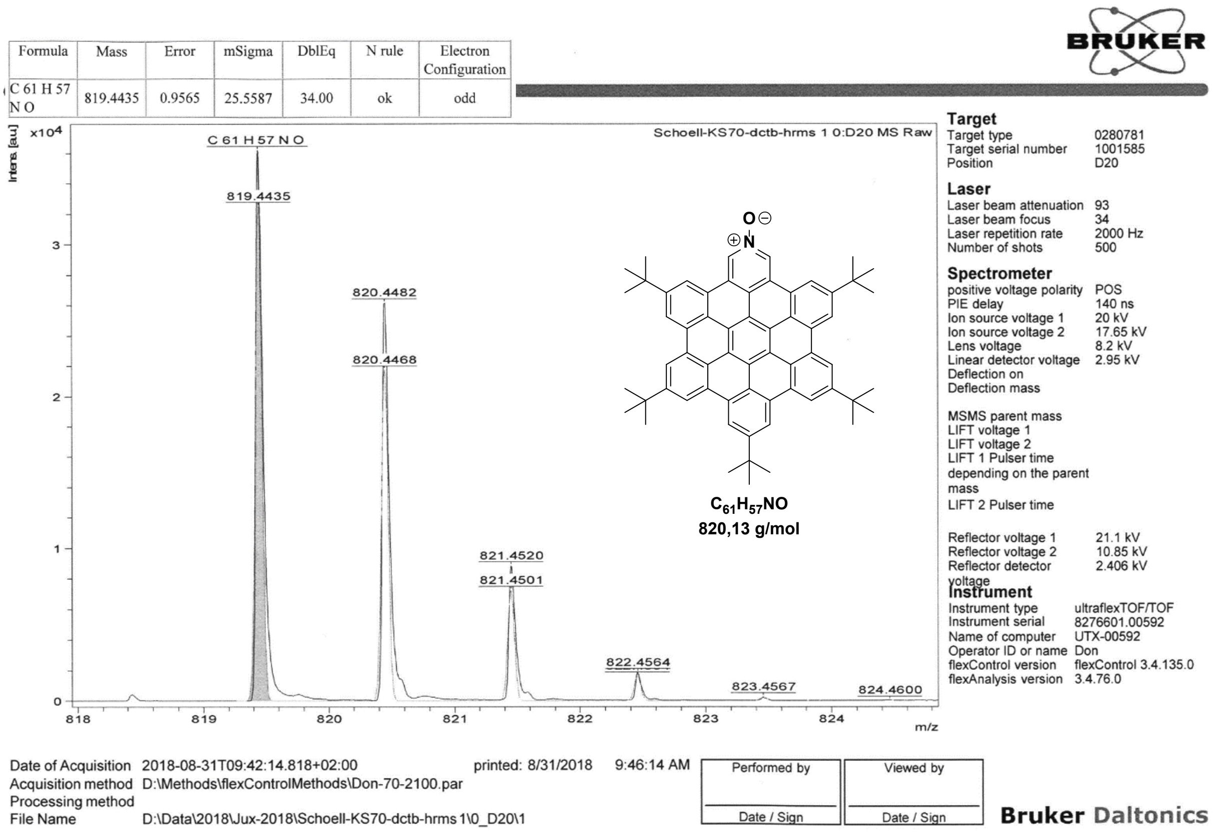

Figure 58. HRMS (MALDI, DCTB) of **13.** Measured (black) and calculated (grey) spectra overlaid.

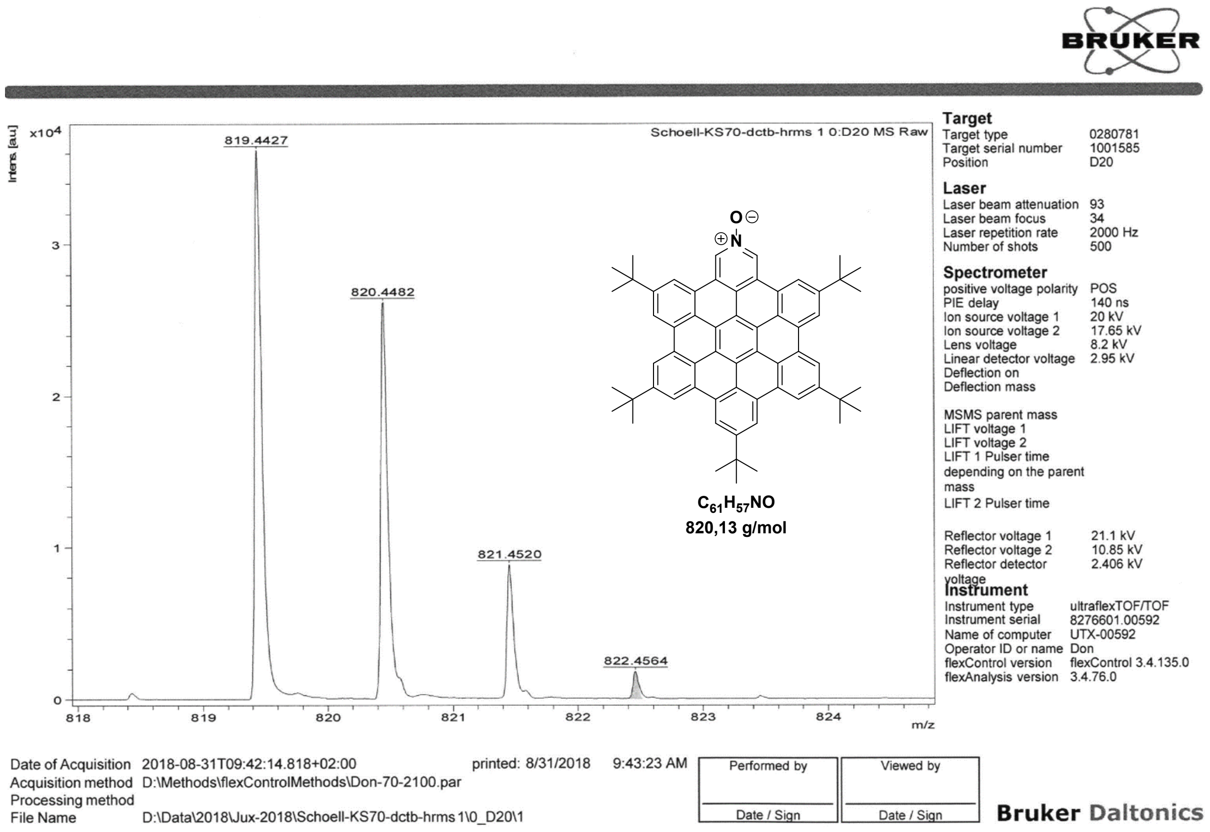

Figure 59. HRMS (MALDI, DCTB) of **13** (Measured spectrum only).

***π*-extended pyridine Zn-porphyrin complex 11**

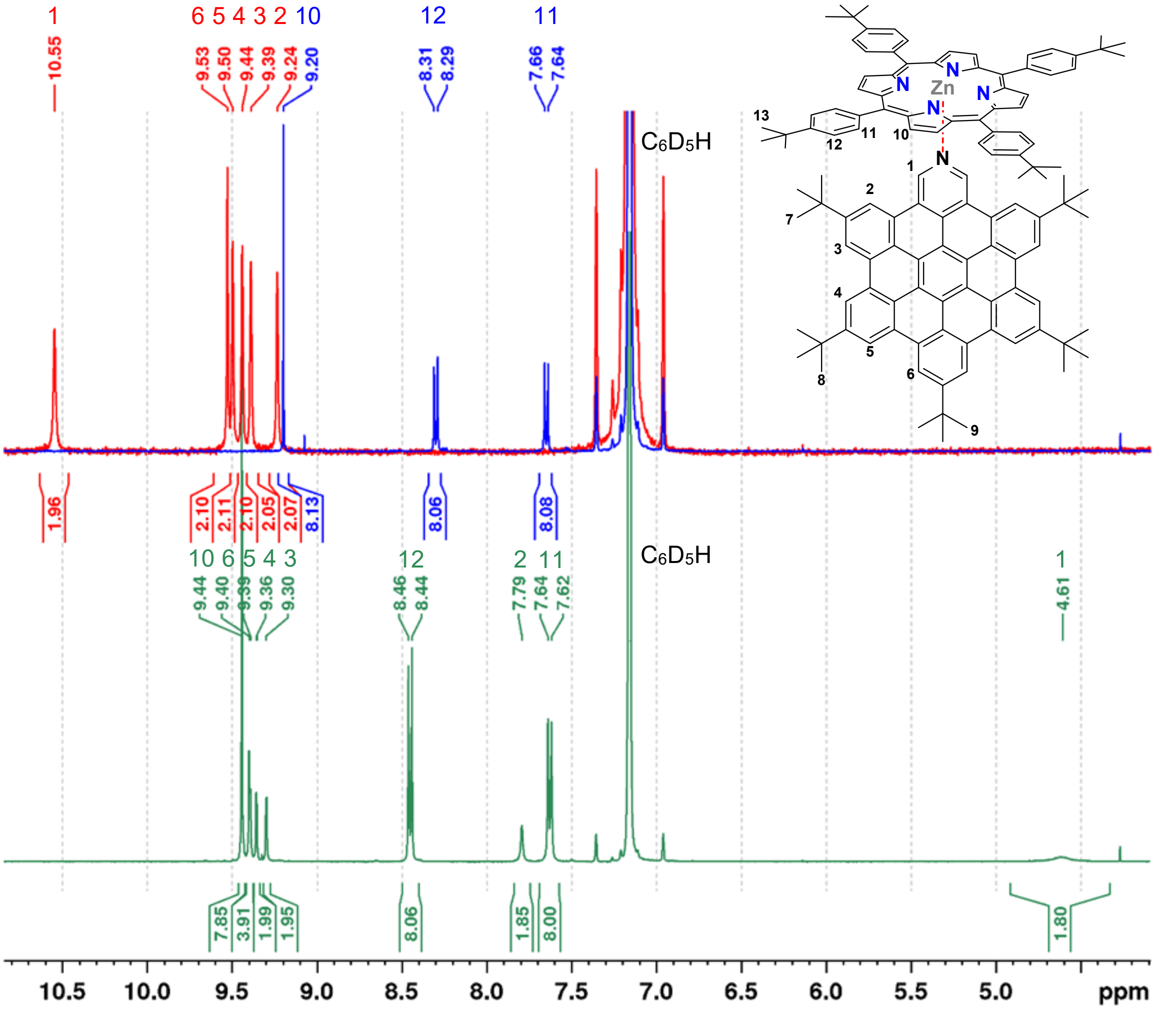

Figure 60. ^{1}H NMR characterization of the *π*-extended pyridine-Zn-porphyrin complex (400 MHz, C_6D_6, rt.). The peaks are labelled according to the complex shown (top right). For clarity only the aromatic region of the spectrum is displayed as the signals of the *tert*-butyl substituents are less influenced by the complex formation. Blue: Zn-porphyrin; red: *π*-extended pyridine; green: *π*-extended pyridine-Zn-porphyrin complex **11**.

Table 10. List of ^1H NMR peaks (400 MHz, C_6D_6, rt.) for *π*-extended pyridine **10b**, Zn-porphyrin and the respective complex **11**. Numbers of peaks according to figure 60.

Peak	Shift for **10b** [ppm]	Shift for Zn-porphyrin [ppm]	Shift for complex [ppm]
1	10.55	-	4.61
2	9.24	-	7.79
3	9.39	-	9.30
4	9.44	-	9.36
5	9.50	-	9.39
6	9.53	-	9.40
7	1.68	-	1.63
8	1.78	-	1.71
9	1.81	-	1.63
10	-	9.20	9.44
11	-	7.65	7.63
12	-	8.30	8.45
13	-	1.50	1.46

6 Assignment of signals for 10b

6.1 ^{1}H NMR assignment

All ^{1}H NMR signals of **10b** could be assigned to the corresponding atoms via utilization of various NMR techniques namely ^{1}H, NOE and COSY.

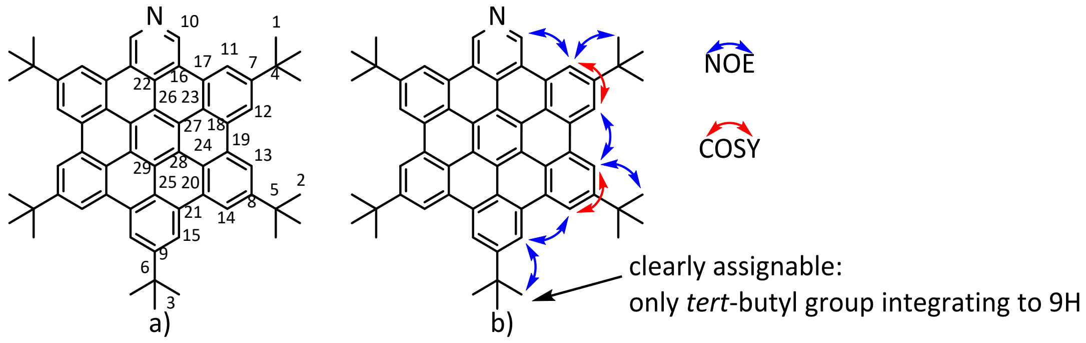

Figure 61. a) Numbering of the atoms in **10b**; b) Schematic strategy for the assignment of the H-atoms in **10b**.

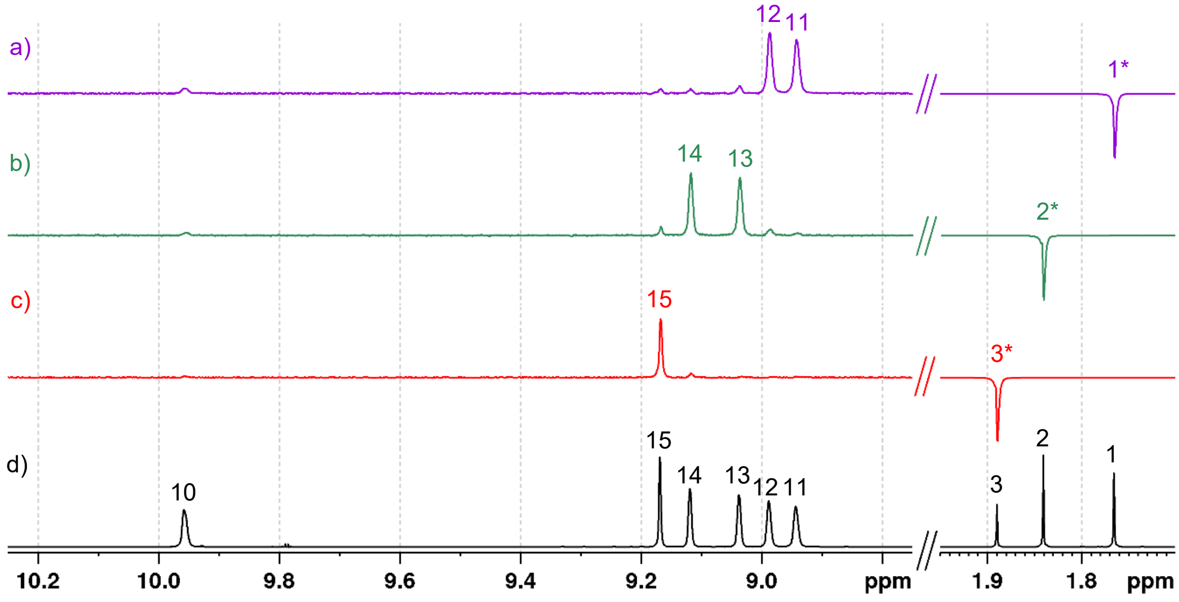

Figure 62. NOE measurements of **10b**. a) (purple) signal for H1 selected; b) (green) signal for H2 selected; c) (red) signal for H3 selected; d) (black) ^{1}H NMR spectrum (600 MHz, $CDCl_3$/NEt_3, rt.) of **10b** for comparison. The selected signals are marked with an asterisk.

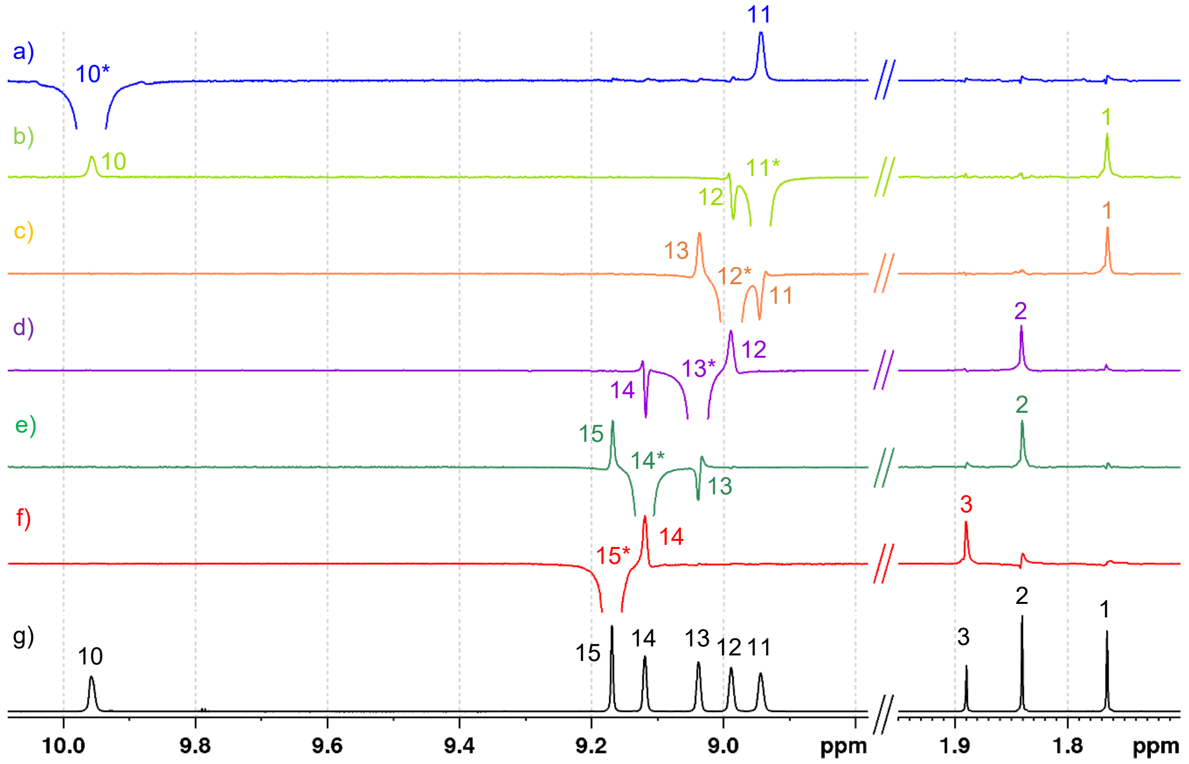

Figure 63. NOE measurements of **10b**. a) (blue) signal for H10 selected; b) (lime) signal for H11 selected; c) (orange) signal for H12 selected; d) (purple) signal for H13 selected; e) (dark green) signal for H14 selected; f) (red) signal for H15 selected; g) (black) ^{1}H NMR spectrum (600 MHz, $CDCl_3$/NEt_3, rt.) of **10b** for comparison. The selected signals are marked with an asterisk.

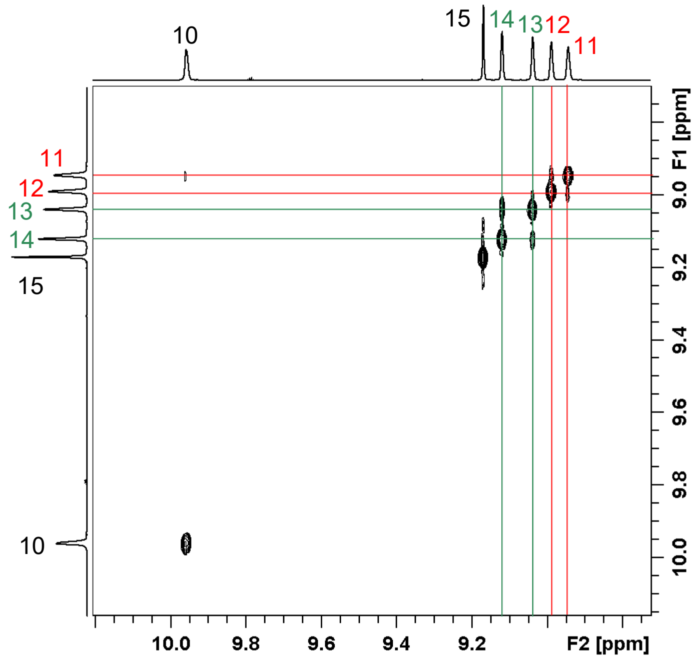

Figure 64. COSY of **10b** with visible ^{4}J couplings.

6.2 ^{13}C NMR assignment

All ^{13}C NMR signals of **10b** could be assigned to the corresponding atoms via utilization of various NMR techniques namely DEPTq-135, HSQC, HMBC and HOESY.

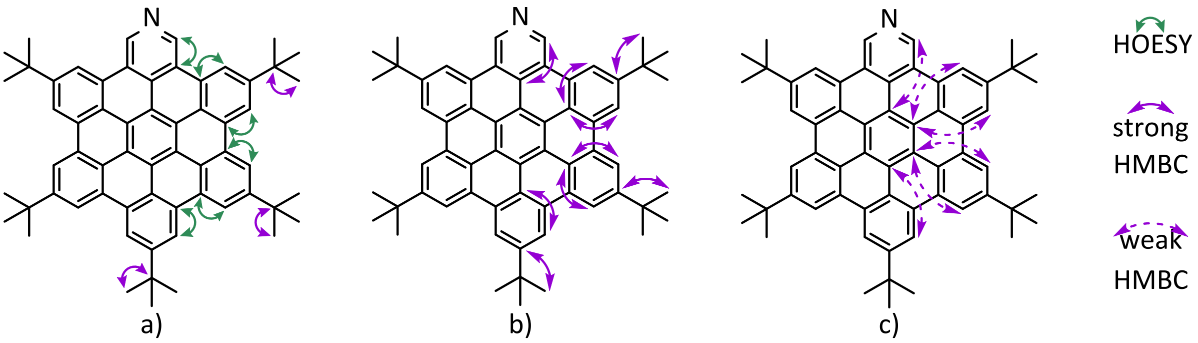

Figure 65. Schematic strategy for the assignment of the C-atoms in **10b**.

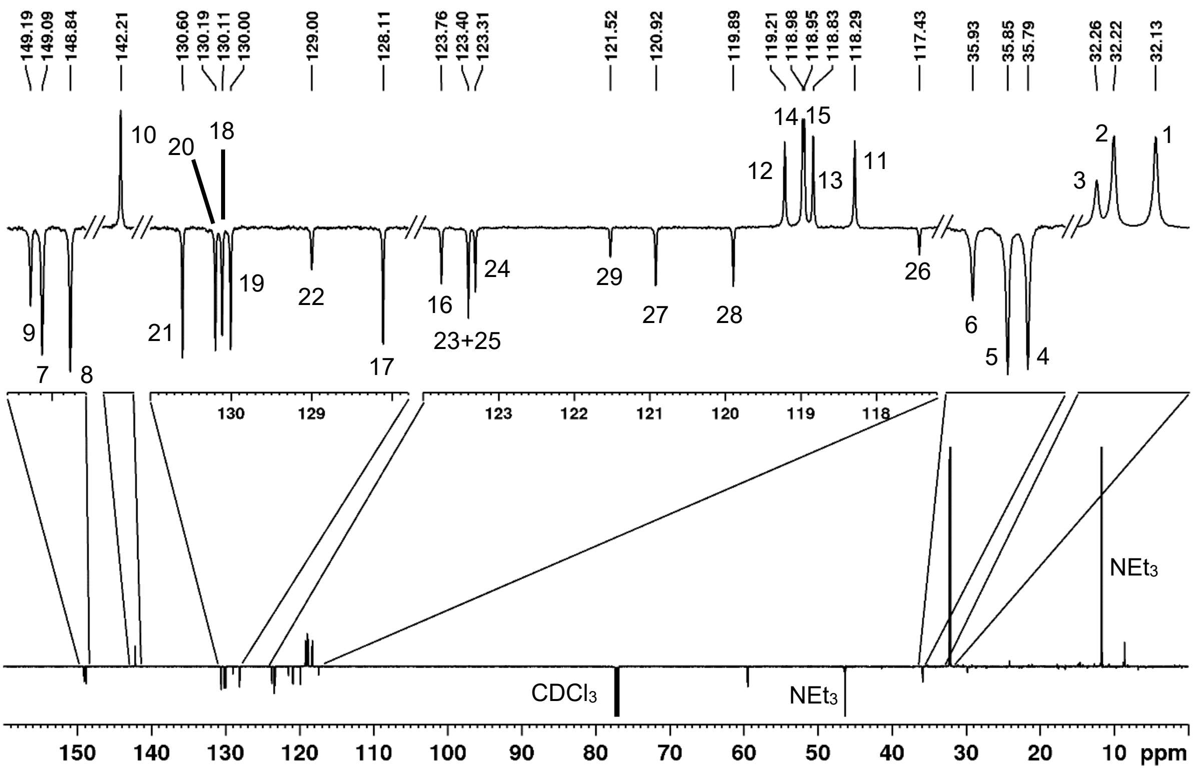

Figure 66. Assigned DEPTq-135 spectrum of **10b** (150 MHz, $CDCl_3$/Et_3N, rt).

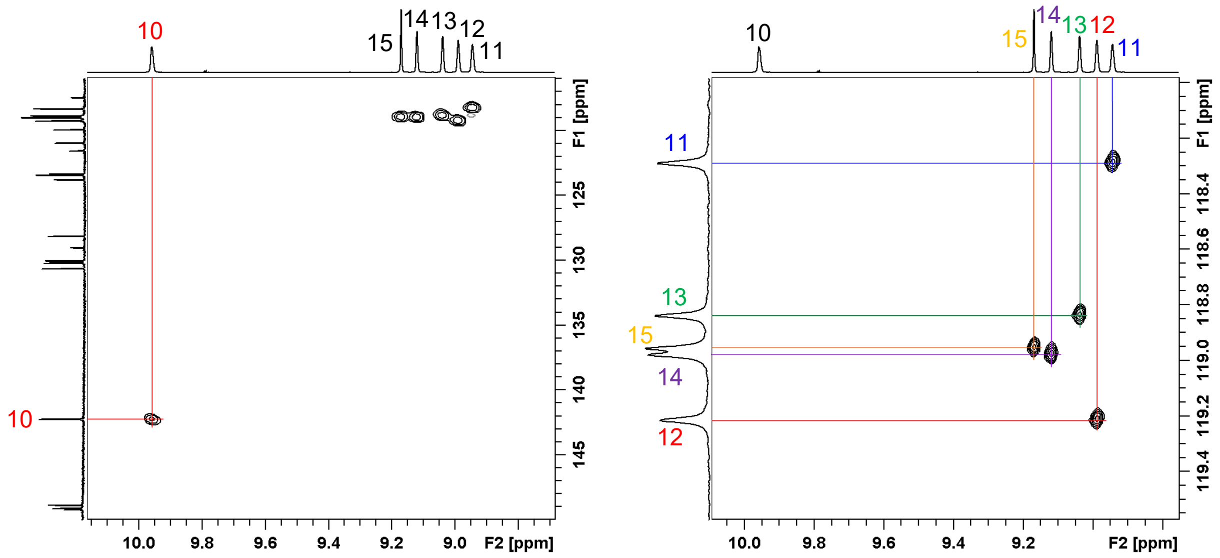

Figure 67. Left: HSQC (aromatic region) of **10b** used for the assignment of atom 10. Right: Selective HSQC of **10b** used for the assignment of atoms 11-15.

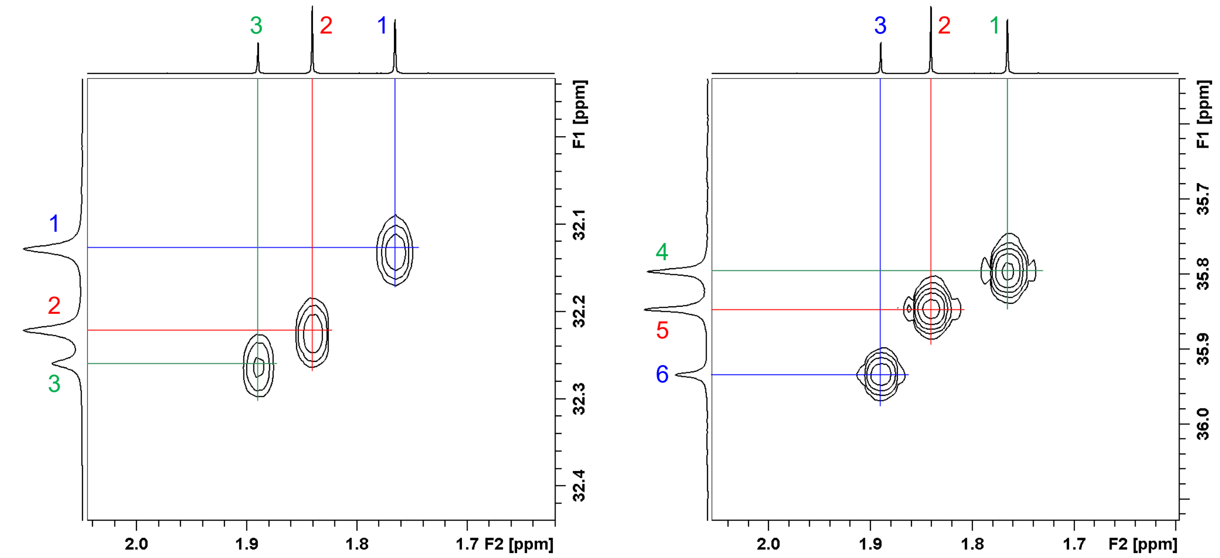

Figure 68. Left: Selective HSQC of **10b** used for the assignment of atoms 1-3. Right: Selective HMBC of **10b** used for the assignment of atoms 4-6.

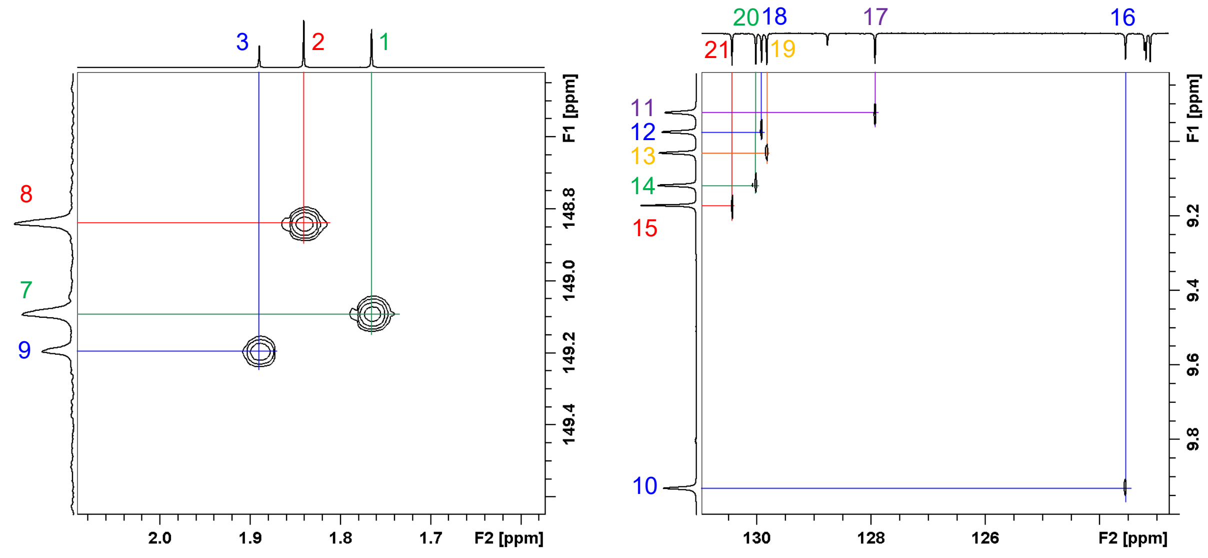

Figure 69. Left: Selective HMBC of **10b** used for the assignment of atoms 7-9. Right: HOESY of **10b** used for the assignment of atoms 1-21.

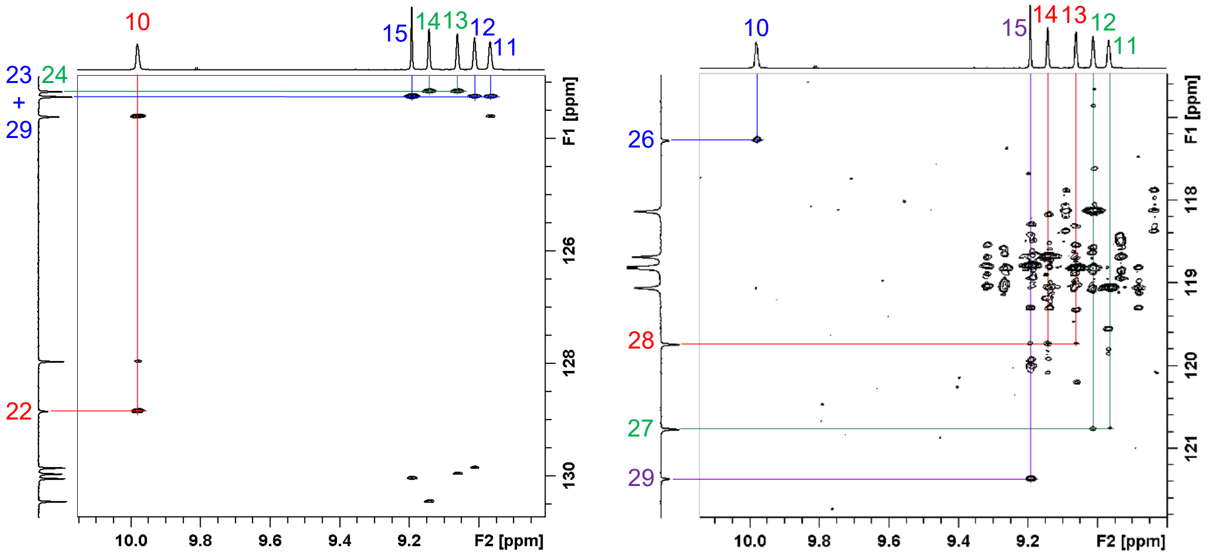

Figure 70. Left: HMBC of **10b** used for the assignment of atoms 22-25. Right: HMBC of **10b** used for the assignment of atoms 26-29.

7 NMR dilution experiments

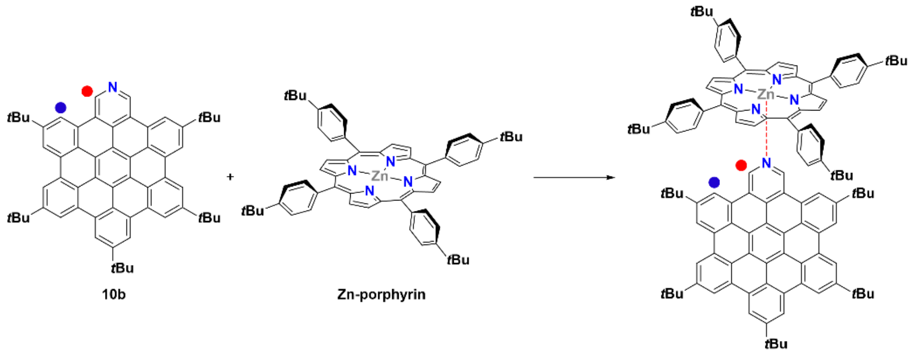

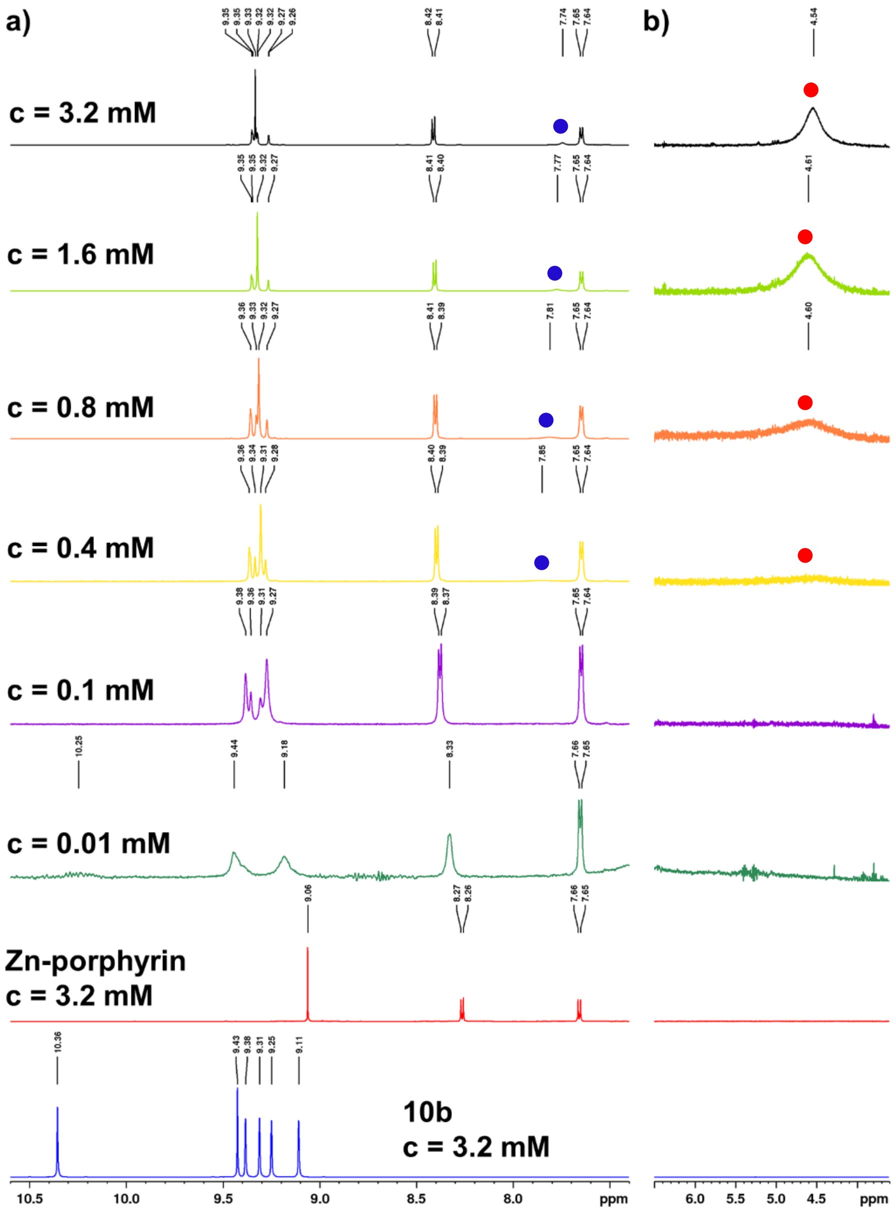

Figure 71. NMR-dilution experiments for complex **11**. Column a) Signals in the aromatic region; column b) region for pyridine-protons 2/6 (red dot) in complex **11**. Note that the two columns have a different zoom for better representation. All spectra in toluene-d8 (600 MHz, rt).

NMR dilution experiments for complex **11** are represented in figure 71. The red and the blue dot mark the proton signals of the pyridine that are most significantly influenced by the complex formation. Spectra in red (Zn-porphyrin)

and blue (**10b**) show the individual compounds at a concentration of 3.2 mM. The black spectrum represents the mixture of **10b** and the Zn-porphyrin at c = 3.2 mM. In this spectrum the complex formation to **11** is clearly indicated by the shift of the pyridine protons (compare blue and black spectra) marked with the dots. Upon lowering the concentration to 1.6 mM, 0.8 mM, and 0.4 mM respectively, the signals for those protons significantly broaden. At even higher dilutions (0.1 mM) the signals completely disappear in the baseline. Close to concentrations usually used for UV/Vis measurements (0.01 mM) there might be a reappearance of the signal for the 2/6 pyridine protons at around 10.25 ppm (which is close to its shift for the individual compound with 10.36 ppm). However, it is unclear if it is really a broad, reappearing signal or a measurement artifact at very low concentrations. Nevertheless, the dilution experiments show that the complex **11** is favoured at higher concentrations (> ~0.5 mM). At lower concentration (~ <0.5 mM) the equilibrium does not favour the complex anymore.

8 Electrochemical characterization

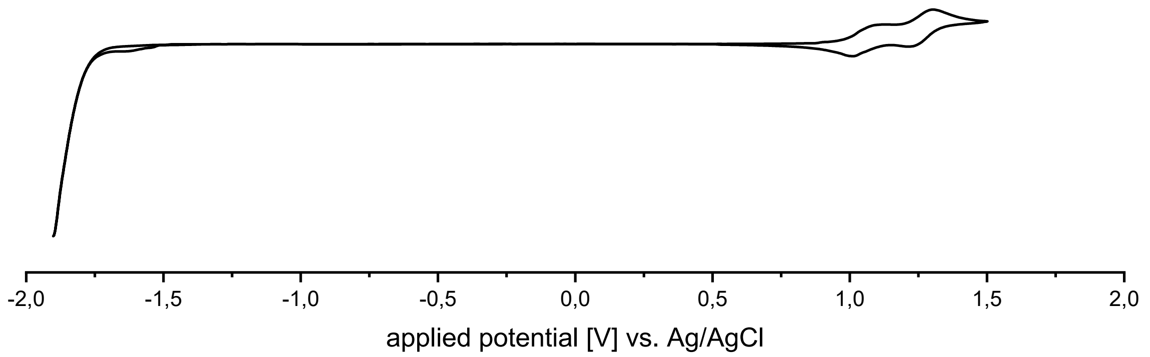

Figure 72. Cyclic voltammogram of **14** in CH_2Cl_2 containing 0.1 M TBA(PF_6) with a scan rate of 50 mV/s.

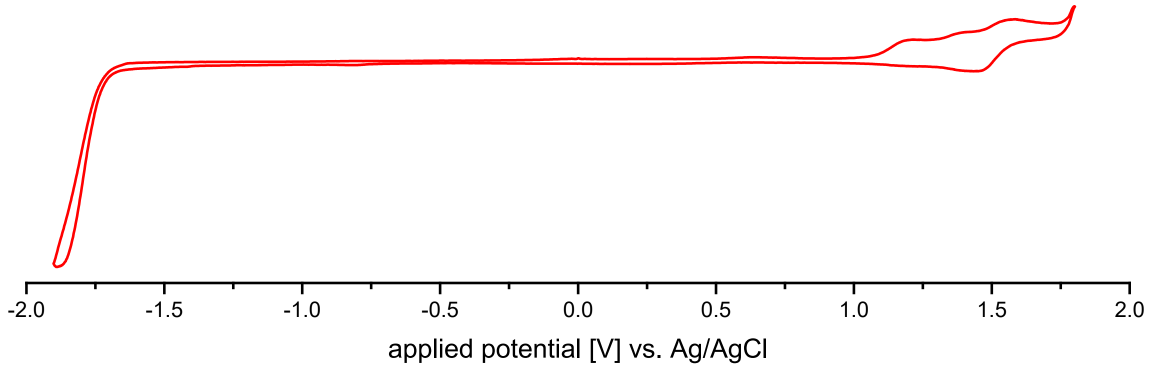

Figure 73. Cyclic voltammogram of **10b** in CH_2Cl_2 containing 0.1 M TBA(PF_6) with a scan rate of 50 mV/s.

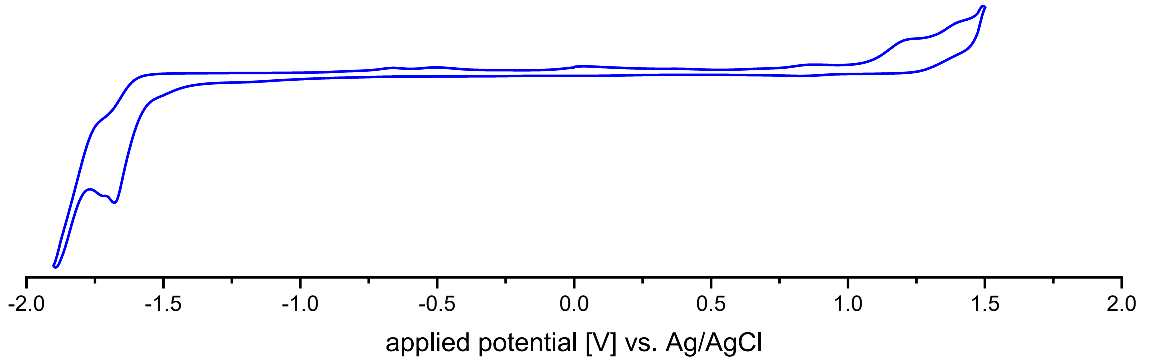

Figure 74. Cyclic voltammogram of **13** in CH_2Cl_2 containing 0.1 M TBA(PF_6) with a scan rate of 50 mV/s.

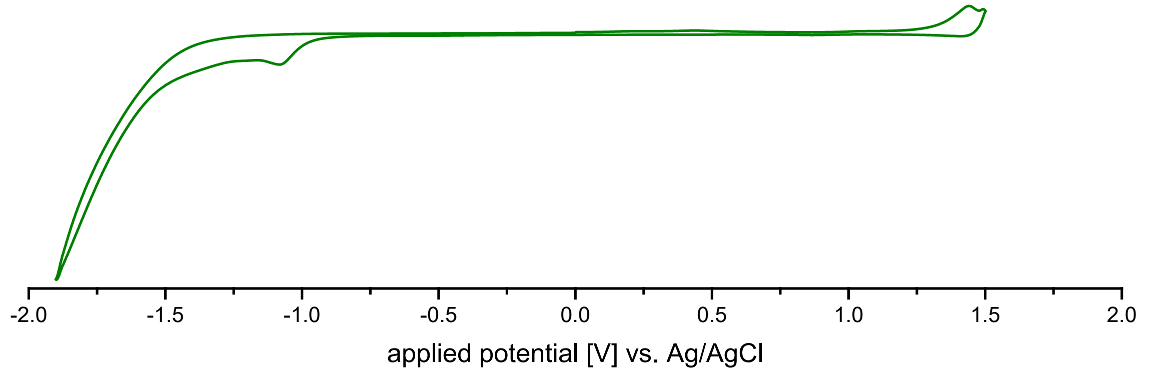

Figure 75. Cyclic voltammogram of **12** in CH_2Cl_2 containing 0.1 M TBA(PF_6) with a scan rate of 50 mV/s.

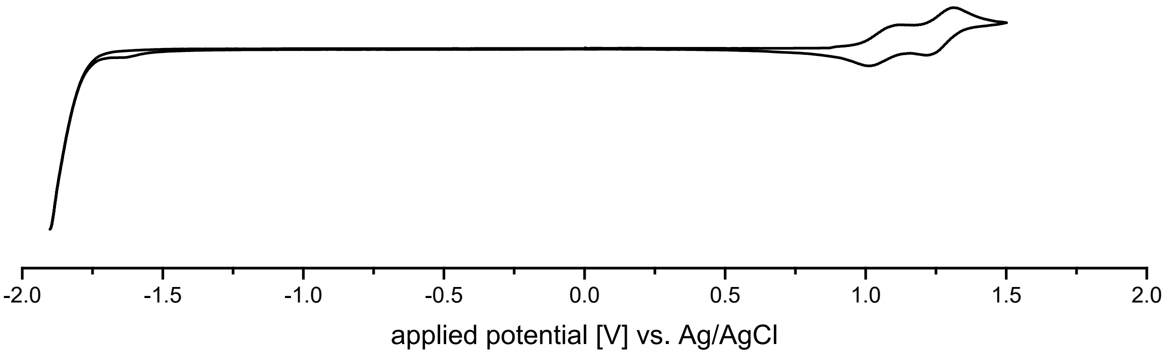

Figure 76. Cyclic voltammogram of **14** in CH_2Cl_2 containing 0.1 M TBA(PF_6) with a scan rate of 100 mV/s.

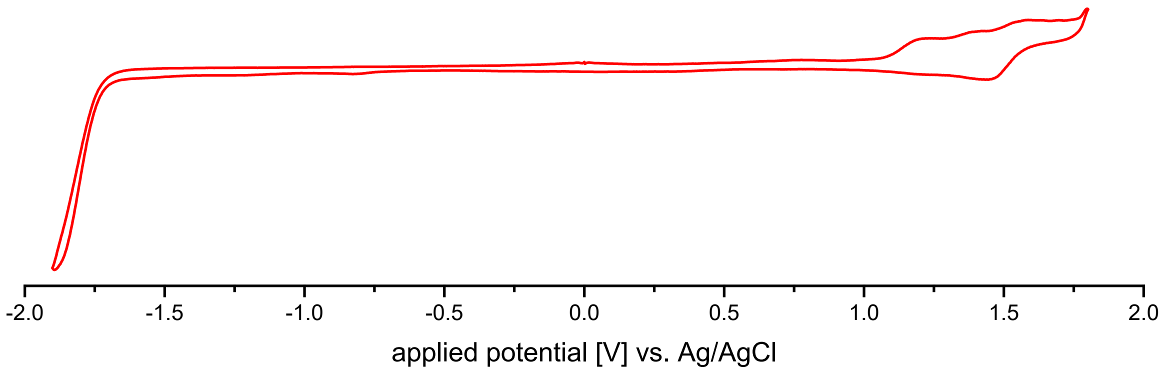

Figure 77. Cyclic voltammogram of **10b** in CH_2Cl_2 containing 0.1 M TBA(PF_6) with a scan rate of 100 mV/s.

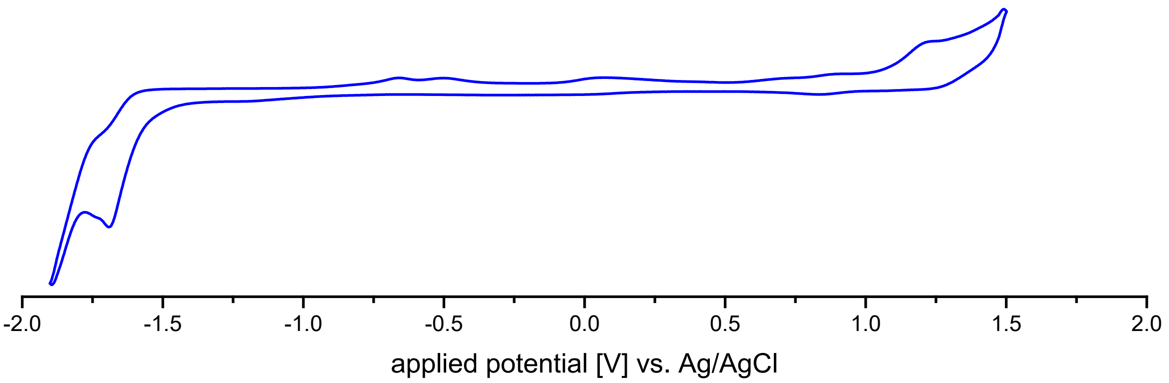

Figure 78. Cyclic voltammogram of **13** in CH_2Cl_2 containing 0.1 M TBA(PF_6) with a scan rate of 100 mV/s.

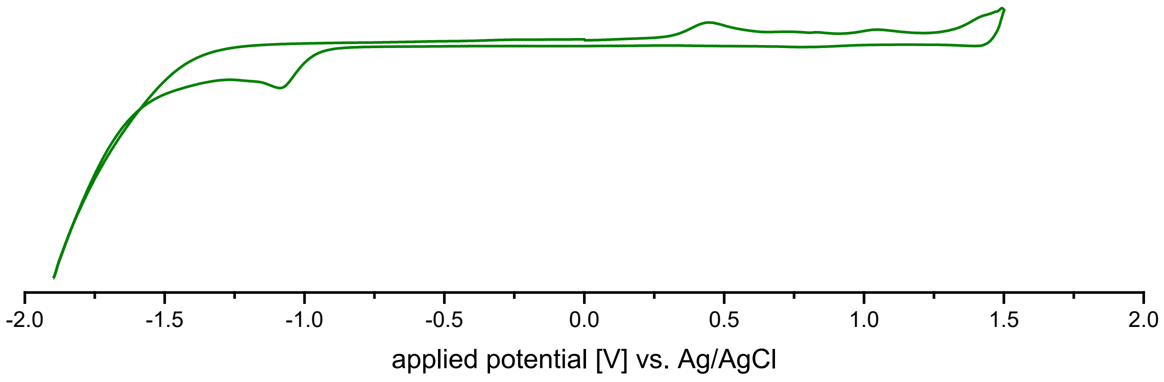

Figure 79. Cyclic voltammogram of **12** in CH_2Cl_2 containing 0.1 M TBA(PF_6) with a scan rate of 100 mV/s.

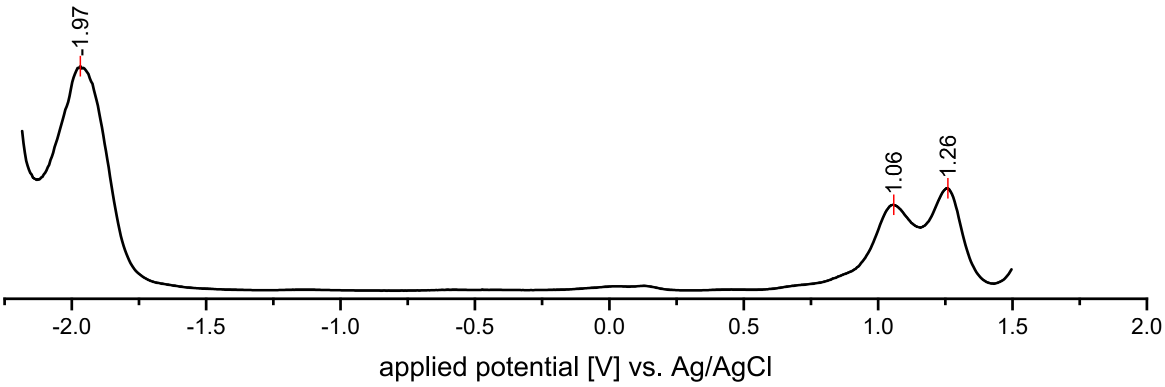

Figure 80. Differential pulse voltammogram of **14** in CH_2Cl_2 containing 0.1 M TBA(PF_6) with a scan rate of 10 mV/s.

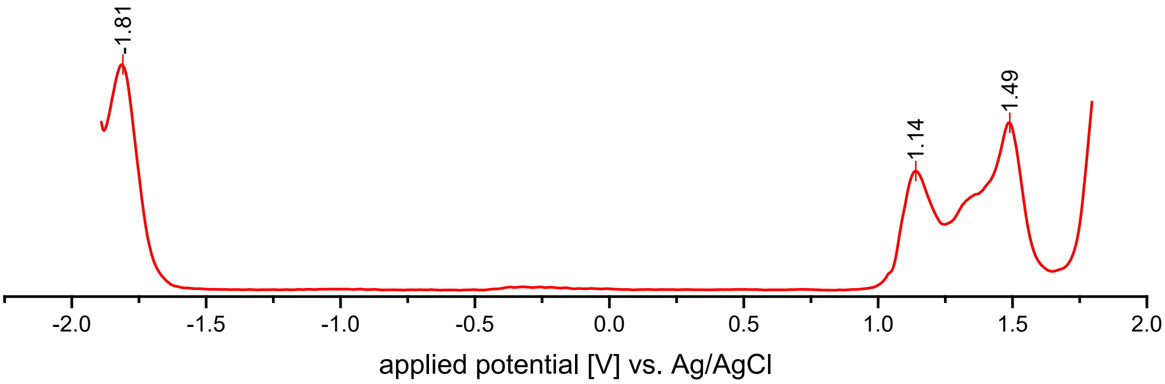

Figure 81. Differential pulse voltammogram of **10b** in CH_2Cl_2 containing 0.1 M TBA(PF_6) with a scan rate of 10 mV/s.

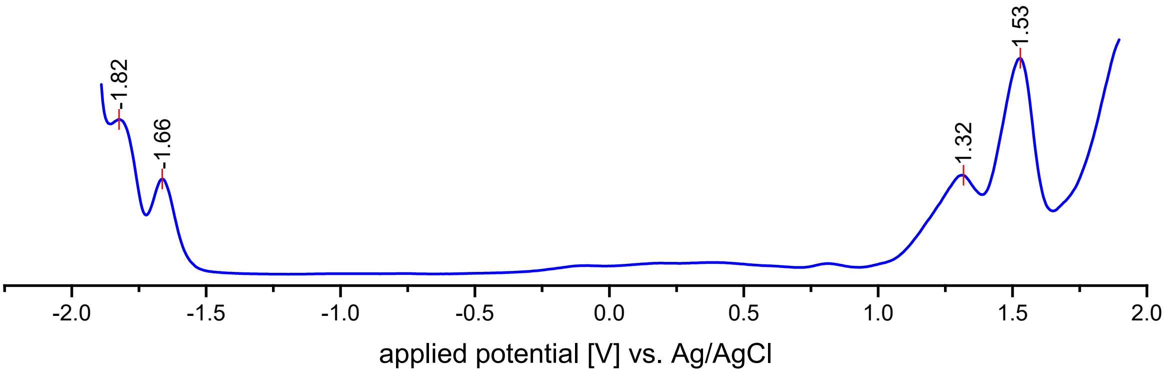

Figure 82. Differential pulse voltammogram of **13** in CH_2Cl_2 containing 0.1 M TBA(PF_6) with a scan rate of 10 mV/s.

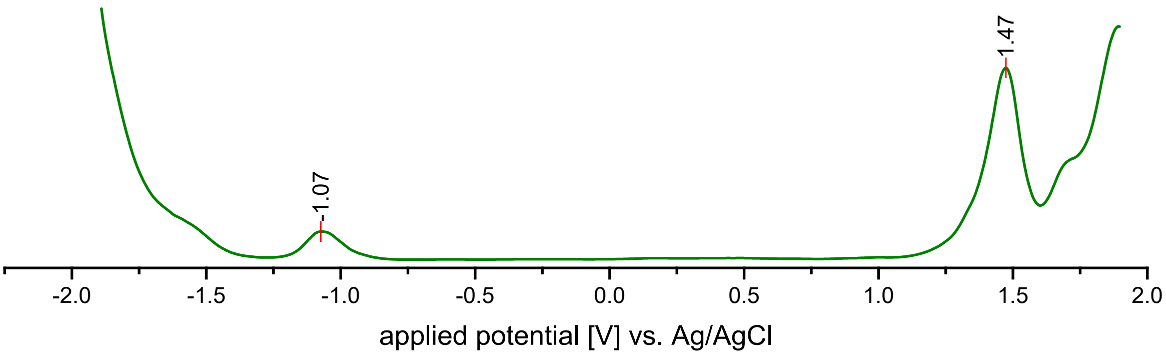

Figure 83. Differential pulse voltammogram of **12** in CH_2Cl_2 containing 0.1 M TBA(PF_6) with a scan rate of 10 mV/s.

9 Literature

[S1]: Lungerich, D., Reger, D., Hölzel, D., Riedel, R., Martin, M. M. J. C., Hampel, F. & Jux, N. *Angew. Chem. Int. Ed.* **55**, 5602-5605 (2016).

[S2]: H. Nöth, B. Wrackenmeyer, NMR — Basic Principles and Progress, Vol. 14 (Eds.: P. Diehl, E. Fluck, R. Kosfeld), Springer, Berlin, **1978**.

[S3]: S. Boldt, S. Parpart, A. Villinger, P. Ehlers, P. Langer, *Angew. Chem. Int. Ed.* 2017, *56*, 4575-4578.

[S4]: S. K. Sadhukhan, C. Viala, A. Gourdon, *Synthesis*, **2003**, *10*, 1521-1525.

[S5]: G. M. Sheldrick, *Acta Cryst. A*, **2015**, *71*, 3-8.

[S6]: O. V. Dolomanov, L. J. Bourhis, R. J. Gildea, J. A. K. Howard, H. Puschmann, *J. Appl. Cryst.* **2009**, *42*, 339-341.

[S7]: G. M. Sheldrick, *Acta Cryst. C*, **2015**, *71*, 3-8.
